# A Brief Dermatology Curriculum in Skin Cancer Detection and Prevention to Improve Medical Student Knowledge and Confidence

**DOI:** 10.15766/mep_2374-8265.11049

**Published:** 2020-12-29

**Authors:** Elsy Compres, Kassandra Holzem, Erin Ibler, Jennifer A. Bierman, Alfred W. Rademaker, Roopal V. Kundu

**Affiliations:** 1 Fourth-Year Medical Student, Northwestern University Feinberg School of Medicine; 2 Resident Physician, Department of Dermatology, Northwestern University Feinberg School of Medicine; 3 Resident Physician, Department of Dermatology, Northwestern University Feinberg School of Medicine; 4 Associate Professor, Departments of Medicine and Medical Education, Northwestern University Feinberg School of Medicine; 5 Professor Emeritus, Department of Preventive Medicine, Northwestern University Feinberg School of Medicine; 6 Associate Professor, Departments of Dermatology and Medical Education, Northwestern University Feinberg School of Medicine

**Keywords:** Sun-Protective Behaviors, Skin Cancer, Knowledge, Attitudes, Behaviors, Patient Counseling, Dermatology, Oncology, Preventive Medicine, Primary Care

## Abstract

**Introduction:**

While the incidence of skin cancers continues to rise, there remains a disproportionate lack of introductory training on skin cancer screening and identification of modifiable behaviors in medical curricula. Trainees and students have cited low confidence in their ability to counsel patients and lack of instruction as barriers.

**Methods:**

To address this need, we created a 1-hour didactic lecture based on a cognitive teaching framework for third-year medical students during their core primary care clerkship. The session highlighted visual identification of different skin cancers, factors increasing individual risk, and photoprotective behaviors. Session content was based on American Academy of Dermatology recommendations for skin cancer prevention. An assessment of knowledge, behaviors, and attitudes given before, immediately following, and at 6 months after the session was used to determine efficacy.

**Results:**

One hundred eight students before and immediately after the session demonstrated significantly improved knowledge (mean correct: 71% presession vs. 99% postintervention, *p* < .0001). Based on 39 participants completing 6-month follow-up, knowledge remained improved (mean answered correctly: 80%, *p* < .0001). Confidence in patient counseling on preventive behaviors, risk assessment, and reported likelihood of counseling significantly increased across the three time points (*p* < .0001 for all attitude questions). Specific topics included appropriate referral to a dermatologist, sunscreen application, and dangers of indoor tanning bed usage.

**Discussion:**

Our session on skin cancer screening and prevention demonstrated improvements in medical student knowledge, confidence, and patient counseling likelihood. This introductory curriculum could be adapted for multiple core clerkships or specialties.

## Educational Objectives

By the end of this activity, learners will be able to:

1. Identify the risks of ultraviolet radiation for the development of skin cancers.
2. Identify behaviors that promote protection from development of skin cancers.
3. Correctly define sun-protective factor and proper use of sunscreen for maximum ultraviolet radiation protection.
4. Employ screening methods for early detection of skin cancers.

## Introduction

Skin cancer is the most common cancer in the United States; more cases are diagnosed annually than with all other cancers combined.^1,2^ Twenty percent of all non-Hispanic White Americans will be diagnosed with a form of skin cancer during their lifetime, and the incidence continues to rise.^1^ Additionally, there are significant cost and morbidity, with an estimated $8.1 billion annually devoted to treating skin cancer in the United States and nearly 10,000 deaths annually due to melanoma.^2^ All forms of skin cancer are associated with less morbidity and, in the case of melanoma, mortality when detected and treated early. The 5-year relative survival rate of localized melanoma versus melanoma that has progressed to include a regional lymph node is 99% versus 66%.^3^ Furthermore, significant differences in overall mean melanoma survival rates have been found when comparing minority populations to Caucasians as reported in the Surveillance, Epidemiology, and End Results database (72%–81% for minorities vs. 90% for Caucasians).^4^

The major modifiable risk factor for the development of skin cancer is reduction of ultraviolet radiation exposure. This has been well established and includes both sun exposure and indoor tanning. Sun exposure has been implicated in approximately 90% of nonmelanoma skin cancers, and sunburns have been shown to increase the risk of melanoma.^2,5^ Given the association, indoor tanning has been deemed a carcinogen by the United States Food and Drug Administration and the World Health Organization International Agency for Research on Cancer.^5^ Furthermore, in the case of melanoma, studies have demonstrated association of advanced disease presentation with low socioeconomic status and lack of access to health care services, among other factors.^6,7^ These findings serve to support an overall multidisciplinary approach to increasing general patient awareness and provider training for early skin cancer detection as it stands to make a significant public health impact.

Certain photoprotective behaviors can reduce an individual's risk of developing skin cancer.^2^ Yet several barriers to achieving adequate sun protection in the general population exist. Lack of awareness as well as differing attitudes and behaviors may be the most prevailing limitations.^8^ Health care providers of all specialties often bridge this knowledge gap, and risk-reducing behaviors are more frequently utilized by patients who receive counseling from their health care providers.^9^ However, formal education on sun safety and patient counseling on skin cancer prevention is often lacking in medical school curricula.^10–14^ Across the 4 years of undergraduate medical education, an average of only 16.3 hours (median: 10 hours) is dedicated to the field of dermatology. Furthermore, 85% of educators say that time devoted to teaching dermatology topics has either decreased or remained the same over a 5-year period.^11^ While the majority of practicing primary care providers indicate that melanoma and nonmelanoma skin cancers, as well as sun damage, are within the top three most important dermatology topics relevant to their practices, less than 40% of residents in primary care fields report feeling adequately prepared by their undergraduate medical curriculum to diagnose and treat common dermatologic conditions.^12^ Others have reported that among graduating medical students, self-practices of sun-protective behaviors are poorly executed, suggesting a paucity of education in skin cancer prevention.^15^

These findings demonstrate a need for curricular development in medical education that not only addresses the public health implications of skin cancer screening but also improves provider confidence in patient counseling for broader population reach. Flexibility in the manner of implementation given existing time constraints is also a demonstrated need. In addition, when directed education on sun safety is implemented, durable change in medical students’ knowledge and behaviors can be positively affected with regard to personal sun-protective behaviors.^16,17^ Our session has been developed with the aim of addressing the demonstrated need for this curriculum and adds to previous curricular developments by focusing on components of counseling and screening directly relevant to patient care rather than solely on changing individual medical student behavior.

## Methods

We developed a 60-minute session highlighting the American Academy of Dermatology recommendations for skin cancer prevention and detection (Appendix A).^18^ It was presented to third-year medical students enrolled in our institution who were participating in the core primary care clinical clerkship as part of their didactic lecture series over the course of 1 academic year. Each session was attended by groups of 10–15 students. The session included interactive cues encouraging student participation, with open-ended questions as well as multiple-choice prompts to reinforce concepts. This was consistent with a cognitive teaching framework allowing for real-time discussion and application of the concepts presented. Identification of different skin cancers and risk factors was achieved by patient case presentations using clinical history and images. Similarly, sun-protective behaviors were elicited by the facilitator using multiple-choice prompts and verbal cues, allowing for teach-back opportunities.

Implementation of the session was overseen by a resident physician, and development of the materials was guided by a faculty member in the department of dermatology. Anonymous pre- and immediate postintervention questionnaires were administered prior to and immediately following the educational session to evaluate the effectiveness of the session in improving student knowledge, attitudes towards patient counseling, and individual behaviors regarding skin cancer prevention (Appendices B & C). A 6-month follow-up questionnaire was subsequently used to assess the durability of students’ knowledge and change in attitude or behavior (Appendix D).

The utilized questionnaires consisted of 30 items—15 multiple-choice knowledge questions, each with a correct answer from among five options; nine attitude questions, each measured on a 5-point scale (1 = *not confident* or *not likely,* 5 = *extremely confident* or *extremely likely*); and six categorical behavior questions. The assessment of the session involved a demonstration of knowledge as well as confidence and likelihood of patient counseling. We compared total percentage of knowledge questions answered correctly across time using repeated measures analysis of variance (ANOVA) with Tukey-adjusted pairwise comparison. Answers to the 15 multiple-choice knowledge questions are found in Appendix E. Each attitude question was analyzed similarly. Behavioral questions were compared across time points using McNemar's test. The assessment tool can be adapted to include only relevant sections at the discretion of future implementers.

## Results

A total of 108 students responded to the pre- and immediate postsession questionnaires. Thirty-nine students (36%) completed the 6-month questionnaire. Evaluations from students before and immediately after the session demonstrated that their knowledge significantly improved following this educational session (mean percentage answered correctly: 71% preintervention vs. 99% postintervention, *p* < .0001). The 6-month follow-up data demonstrated a statistically significant retention of knowledge as compared to the preintervention (80% 6-month follow-up vs. 71% preintervention, ANOVA *p* < .0001). Students correctly identified the “ABCDEs of melanoma detection” mnemonic readily during the preintervention questionnaire, though questions pertaining to appropriate use of sunscreens were frequently answered incorrectly prior to the educational session.

The students’ confidence with counseling patients on sun-protective behaviors increased (mean value: 3.3 preintervention vs. 4.2 postintervention on a 5-point scale, *p* < .0001). This was further reflected in student confidence in appropriately answering patient questions on risk factors leading to skin cancer as well as the proper use of sunscreen (mean value: 2.6 for risk factors, 2.9 for sunscreen preintervention, vs. 3.8 for risk factors, 4.4 for sunscreen postintervention, *p* < .0001). Students also expressed increased confidence in counseling patients using the recommended practices they were exposed to at the session (mean value: 2.5 preintervention vs. 3.6 postintervention, *p* < .0001). Students stated that they would be more likely to voluntarily counsel their patients on skin cancer prevention as a result of this session (mean value: 2.5 preintervention vs. 3.6 postintervention, *p* < .0001).

The intervention also effected student attitude changes as seen in an increased likelihood of including sun-protective practices and indoor tanning bed usage as part of the routine social history (1.9 for sun-protective practices, 1.4 for indoor tanning bed usage preintervention, vs. 3.0 for sun-protective practices, 2.9 for indoor tanning bed usage postintervention, on a 5-point scale, *p* < .0001). The session further influenced students’ confidence levels in appropriately referring their patients to skin cancer screening (3.1 preintervention vs. 4.2 postintervention, *p* < .0001). Moreover, the short intervention resulted in an increased confidence level in recognizing skin lesions concerning for skin cancer during routine physical exams (2.8 preintervention vs. 3.7 postintervention, *p* <.0001). Overall, student attitude scores remained significantly increased as compared to preintervention values at the time of the 6-month follow-up across all nine questions.

Finally, assessment focused on students’ personal usage of sun-protective behaviors and skin self-examinations. The percentage of students reporting that they personally used sunscreen when going outdoors increased between the preintervention and 6-month follow-up (70% preintervention vs. 80% 6-month follow-up, *p* = .30). Students also demonstrated an increase in conducting personal skin checks (57% preintervention vs. 64% 6-month follow-up, *p* = .47). A greater improvement was noted in students reporting attempts to avoid excessive sun exposure (66% preintervention vs. 77% 6-month follow-up, *p* = .20). While these personal sun-protective behaviors did improve over the follow-up period, this improvement was not statistically significant.

Questions quantifying behavior occurrences also demonstrated increases in the 6-month time frame. Students showed an increase in the number of patients to whom they recommended a skin cancer screening after visual examination, with 32% reporting one to five encounters in the preintervention and 47% reporting one to five encounters in the 6-month follow-up (*p* = .23). Student counseling experiences involving the risks and benefits of skin cancer and prevention demonstrated modest improvement following the session, with 40% reporting one to five encounters in the preintervention versus 42% in the 6-month follow-up (*p* = .72). Tables 1 and 2 provide an overall summary of the assessment results.

**Table 1.**
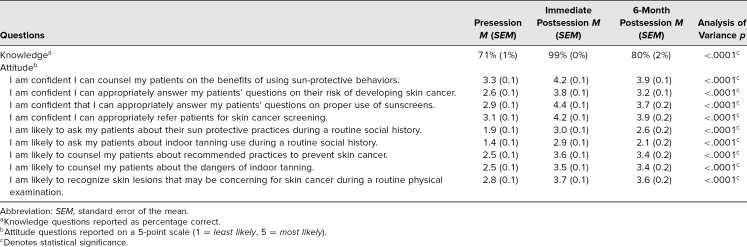
Assessment Results for Knowledge and Attitude Questions Following Didactic Session on Sun Protection

**Table 2.**
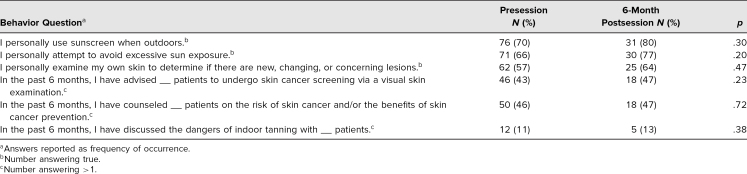
Assessment Results for Behavior Questions Following Didactic Session on Sun Protection

## Discussion

Our assessment indicated a statistically significant increase in both knowledge and student confidence in patient counseling following a brief interactive didactic session. Both of these needs have been identified as current barriers in general patient care.^13,14,19^ Previously, innovations to fill the need for enhanced general dermatologic curricula in medical education have been proposed and have similarly shown improvements in knowledge and behavior.^16,17,20,21^ Our session adds to these efforts and also focuses specifically on addressing the current gap in skin cancer screening and preventive counseling in practice. Furthermore, we include a more in-depth and tailored knowledge assessment. We have also used our assessment to validate patient counseling behaviors and confidence levels, specifically demonstrating improvement on previous literature stating that 52% of fourth-year medical students self-report being unskilled in skin cancer detection and counseling.^19^ While our session is of shorter duration than others we cite here, we hope that its demonstrated impact as well as its narrowed focus will allow it to serve other members of the medical community engaged in patient care who face time and resource constraints. Even though we have focused our presentation on third-year medical students, the session and assessment materials are adaptable to multiple audiences across specialties and can include residents, physician assistants, and nurses.

Due to the high and rising incidence of skin cancer, most health care providers will invariably encounter patients at risk for it. Therefore, our overall objective is to promote more patient counseling on skin cancer prevention, in order to aid in increasing patient awareness and potentially reducing the disease burden of nonmelanoma and melanoma skin cancer. As medical school curricula progress towards more integrative models of learning, sessions such as this one could provide simple and effective solutions for knowledge dissemination with a minimal time commitment and very low financial cost to achieve a lasting impact.

Our session allowed for audience participation throughout using interactive cues. The spacing of multiple-choice questions as well as questions throughout content slides aided in creating an interactive learning environment. Practical application of the knowledge being disseminated as well as critical thought patterns necessary to its application were therefore encouraged. While we ultimately reached 108 students with the session, it is important to emphasize that the session was held repeatedly over the course of 1 academic year as part of the primary care clerkship curriculum. Therefore, the session was able to be implemented repeatedly to achieve assessment outcomes. The small-group implementation strategy we utilized contributed to creating an interactive learning environment, and this likely played a role in achieving session efficacy.

Session results also demonstrated a trend towards positive behavior modifications by the students themselves regarding sun-protection practices and skin self-exams across the three time periods. One possible explanation for this finding is that behavioral changes can vary in the time that they are ultimately adopted. Another possible explanation is that the session was framed as an educational didactic session geared for patient care. Despite this, we believe that continuity of education in these practices could result in lasting change over time. The immediate outcome of our study is increasing student confidence in and likelihood of providing quality patient counseling. The 6-month follow-up data illustrated statistically significant lasting effects on medical student knowledge and attitudes regarding skin cancer prevention and patient counseling. These findings suggest that the session had an overall impact on patient care and improved overall comfort with the information and skills presented. This skill set alone is expandable across multiple medical disciplines as identifying common risk factors for skin cancer and patient usage of sun-protective practices could seamlessly make up part of the routine medical and social history.

The study is limited by the number of participants who voluntarily completed the questionnaire before and after the didactic session. Out of 108 initial participants, only 39 completed the longitudinal 6-month follow-up questionnaire, limiting our ability to make conclusions regarding long-term changes in behaviors or attitudes. Additionally, the surveyed medical student population comprised only third-year medical students at a single institution. Future studies might broaden the student population to include all levels of medical student education as well as education of midlevel providers and allied health professionals. The follow-up period could also be extended beyond 6 months to further determine the durability of similar interventions.

## Appendices

Skin Cancer Prevention Didactic.pptxPretest Survey.docxImmediate Posttest Survey.docxSix-Month Possttest Survey.docxKnowledge Assessment Answer Key.docx All appendices are peer reviewed as integral parts of the Original Publication.
