# Supplementary material for: A Brief Dermatology Curriculum in Skin Cancer Detection and Prevention to Improve Medical Student Knowledge and Confidence
Source: MedEdPORTAL. 2020 Dec 29;16:11049. doi: 10.15766/mep_2374-8265.11049 (PMC7780741; doi:10.15766/mep_2374-8265.11049)
Supplement: Supplementary file 1 — Skin Cancer Prevention Didactic.pptxPretest Survey.docxImmediate Posttest Survey.docxSix-Month Possttest Survey.docxKnowledge Assessment Answer Key.docx [file mep_2374-8265.11049-s001.zip › A. Skin Cancer Prevention Didactic.pptx]

## Slide 1
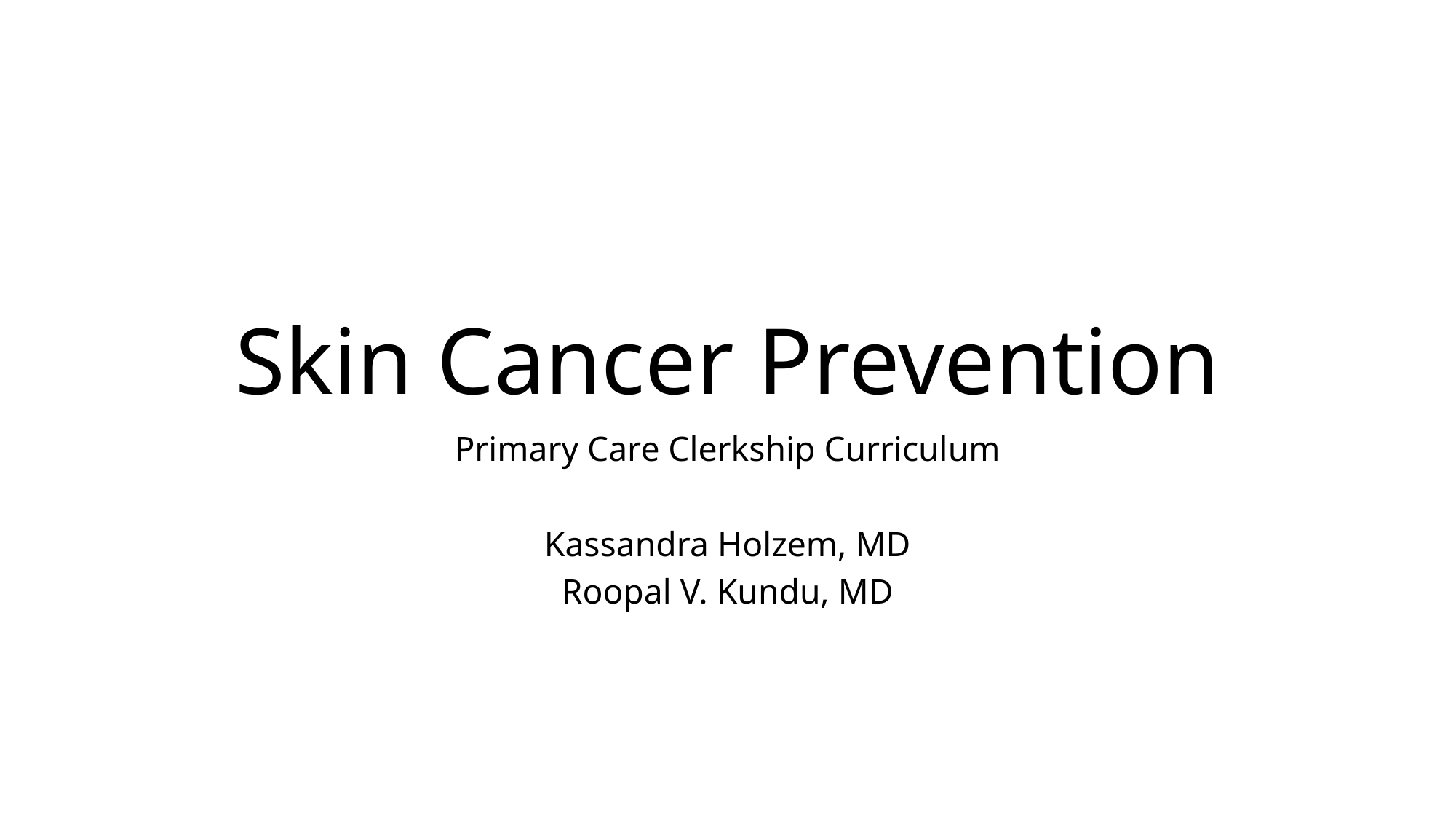

# Skin Cancer Prevention
Primary Care Clerkship Curriculum
Kassandra Holzem, MD
Roopal V. Kundu, MD

## Slide 2
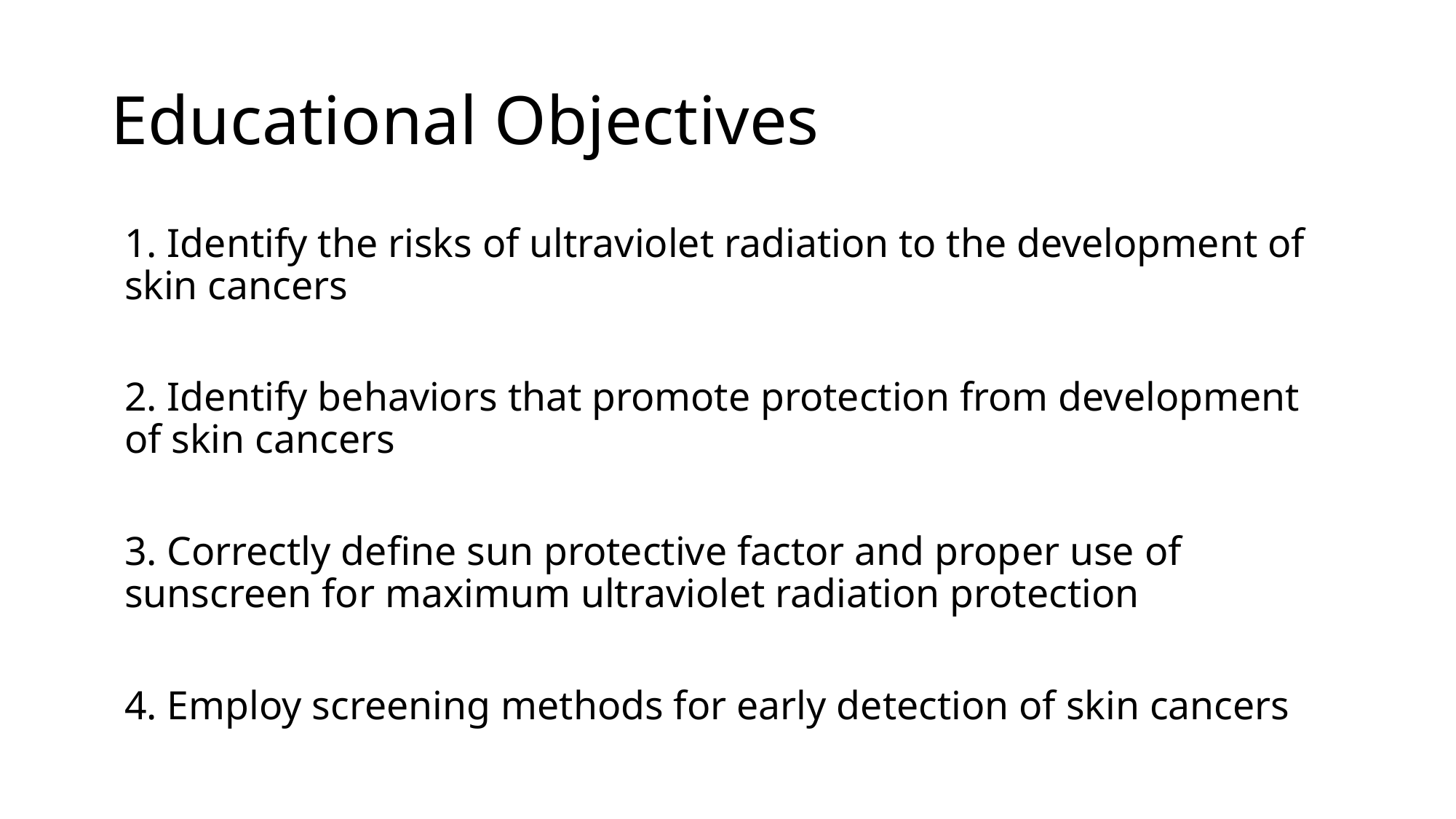

# Educational Objectives
1. Identify the risks of ultraviolet radiation to the development of skin cancers
2. Identify behaviors that promote protection from development of skin cancers
3. Correctly define sun protective factor and proper use of sunscreen for maximum ultraviolet radiation protection
4. Employ screening methods for early detection of skin cancers

## Slide 3
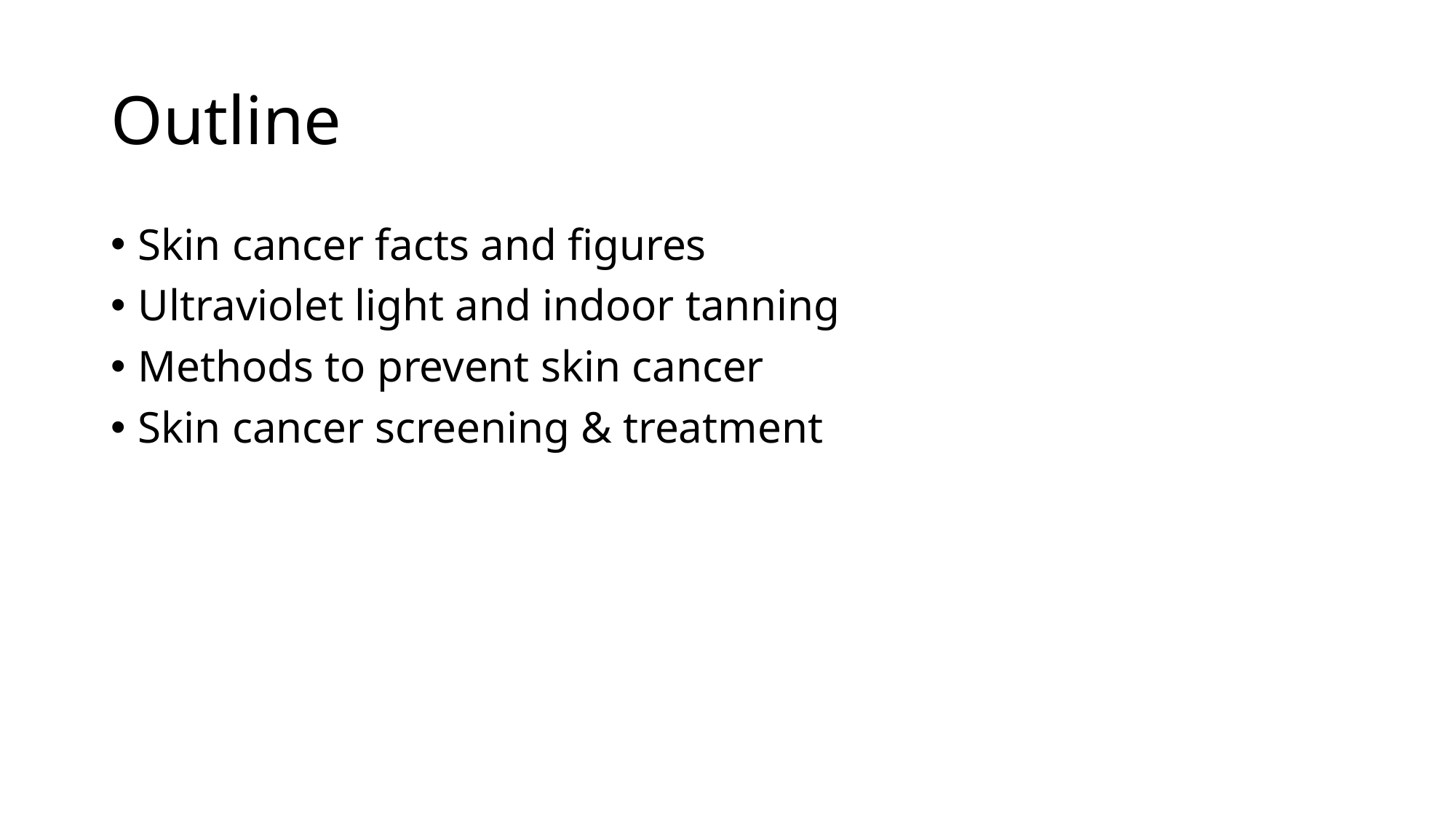

# Outline
Skin cancer facts and figures
Ultraviolet light and indoor tanning
Methods to prevent skin cancer
Skin cancer screening & treatment

## Slide 4
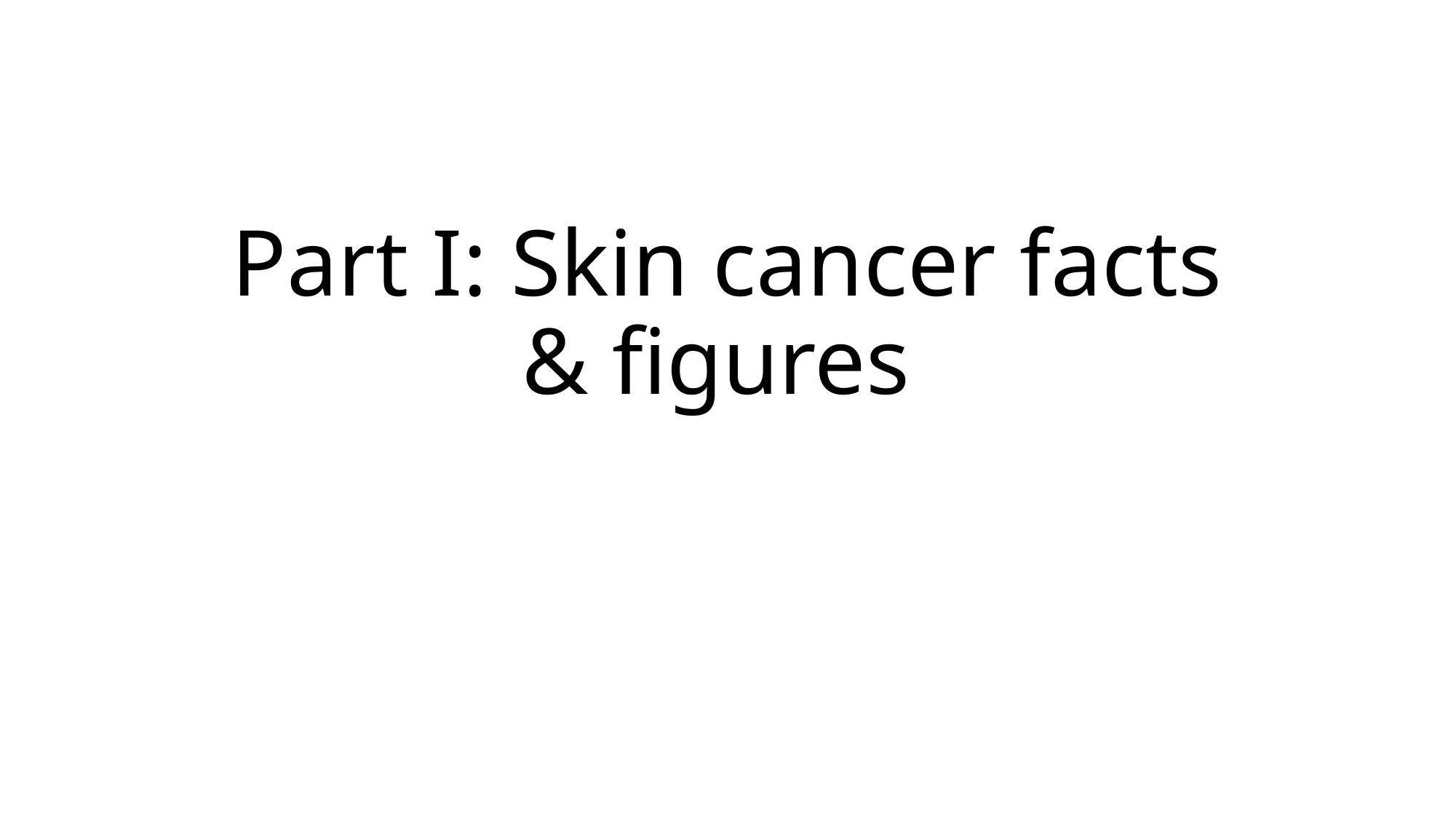

# Part I: Skin cancer facts & figures

## Slide 5
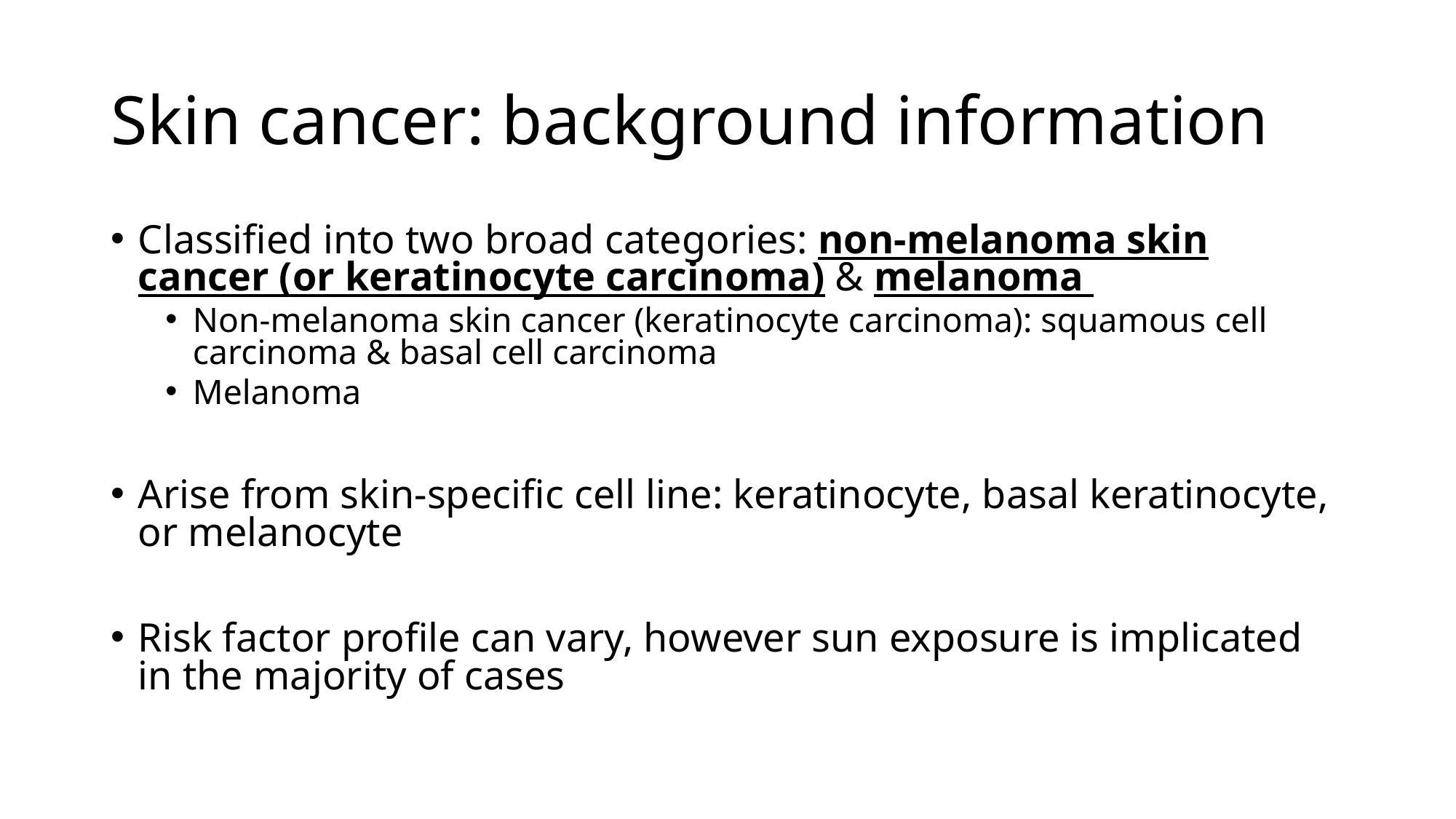

# Skin cancer: background information
Classified into two broad categories: non-melanoma skin cancer (or keratinocyte carcinoma) & melanoma
Non-melanoma skin cancer (keratinocyte carcinoma): squamous cell carcinoma & basal cell carcinoma
Melanoma
Arise from skin-specific cell line: keratinocyte, basal keratinocyte, or melanocyte
Risk factor profile can vary, however sun exposure is implicated in the majority of cases

## Slide 6
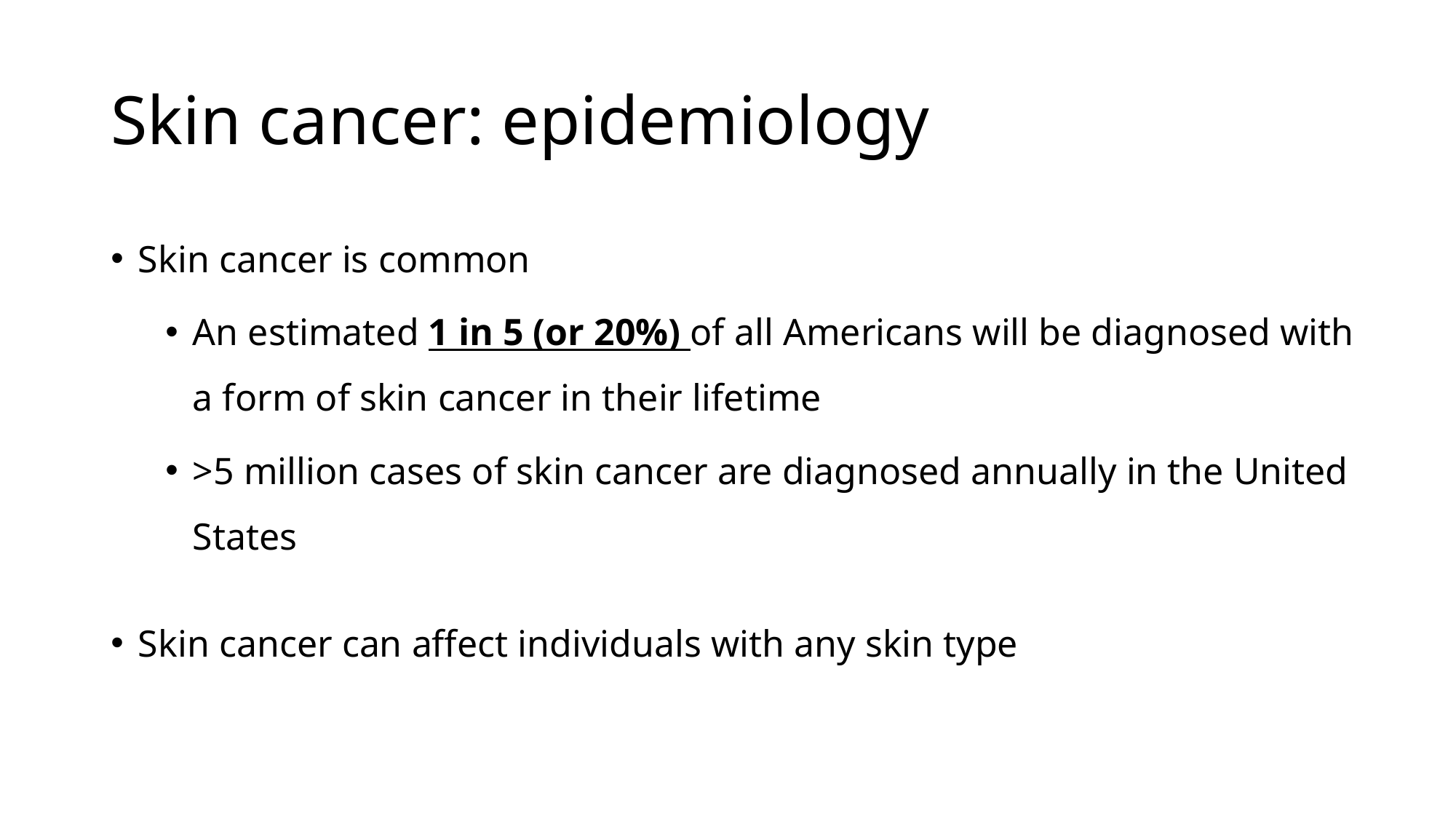

# Skin cancer: epidemiology
Skin cancer is common
An estimated 1 in 5 (or 20%) of all Americans will be diagnosed with a form of skin cancer in their lifetime
>5 million cases of skin cancer are diagnosed annually in the United States
Skin cancer can affect individuals with any skin type

## Slide 7
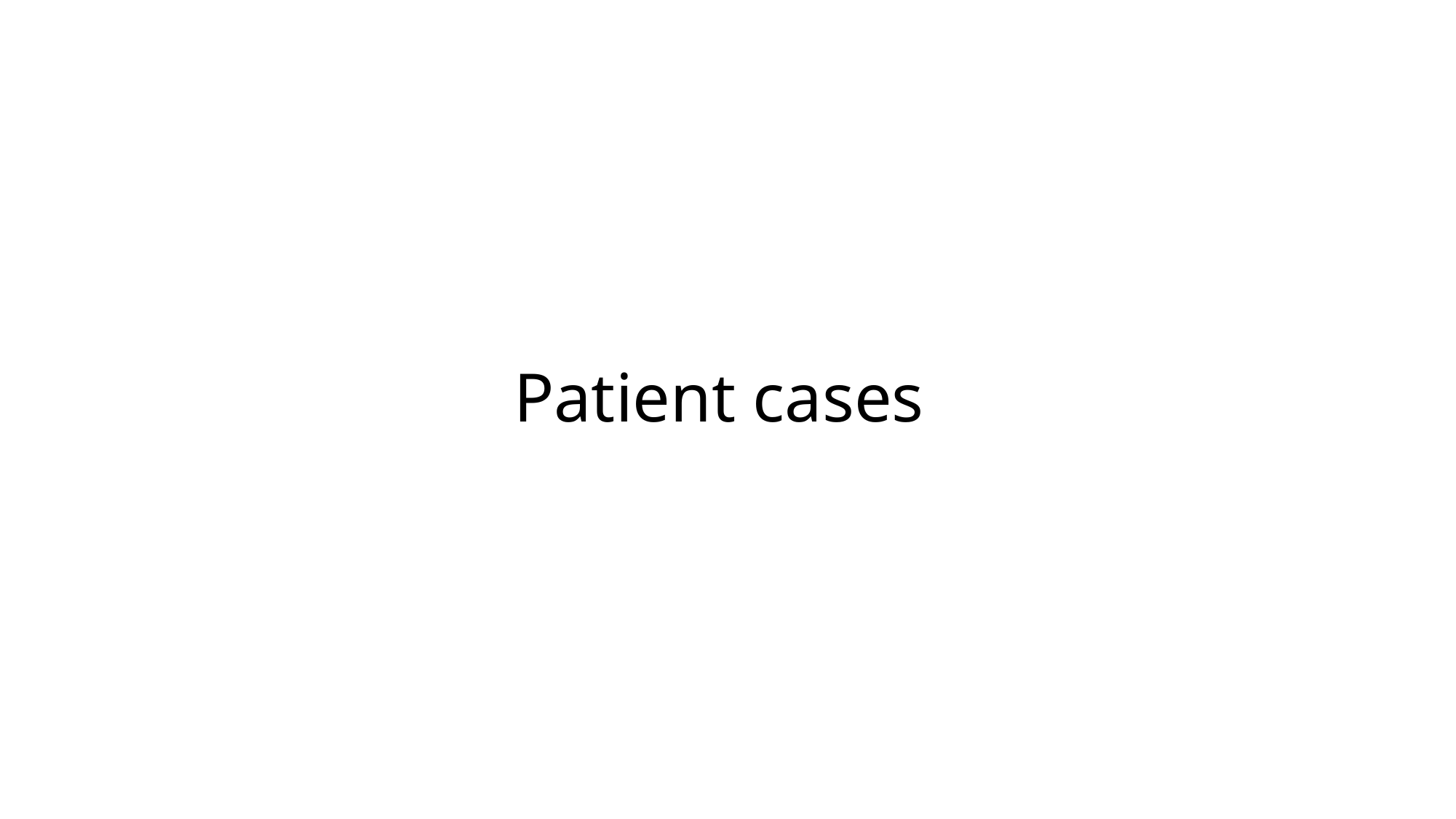

# Patient cases

## Slide 8
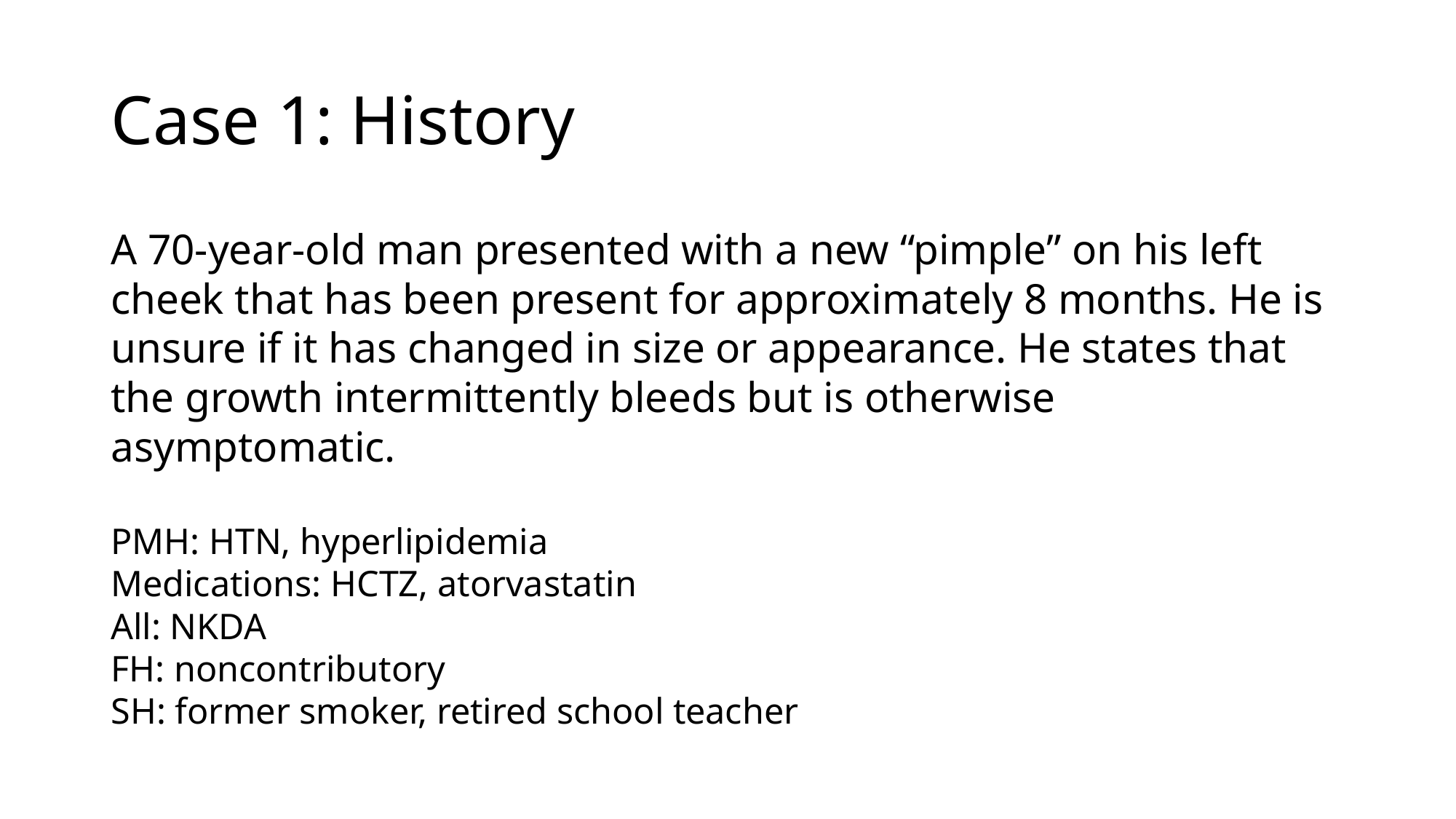

# Case 1: History
A 70-year-old man presented with a new “pimple” on his left cheek that has been present for approximately 8 months. He is unsure if it has changed in size or appearance. He states that the growth intermittently bleeds but is otherwise asymptomatic.
PMH: HTN, hyperlipidemia
Medications: HCTZ, atorvastatin
All: NKDA
FH: noncontributory
SH: former smoker, retired school teacher

## Slide 9
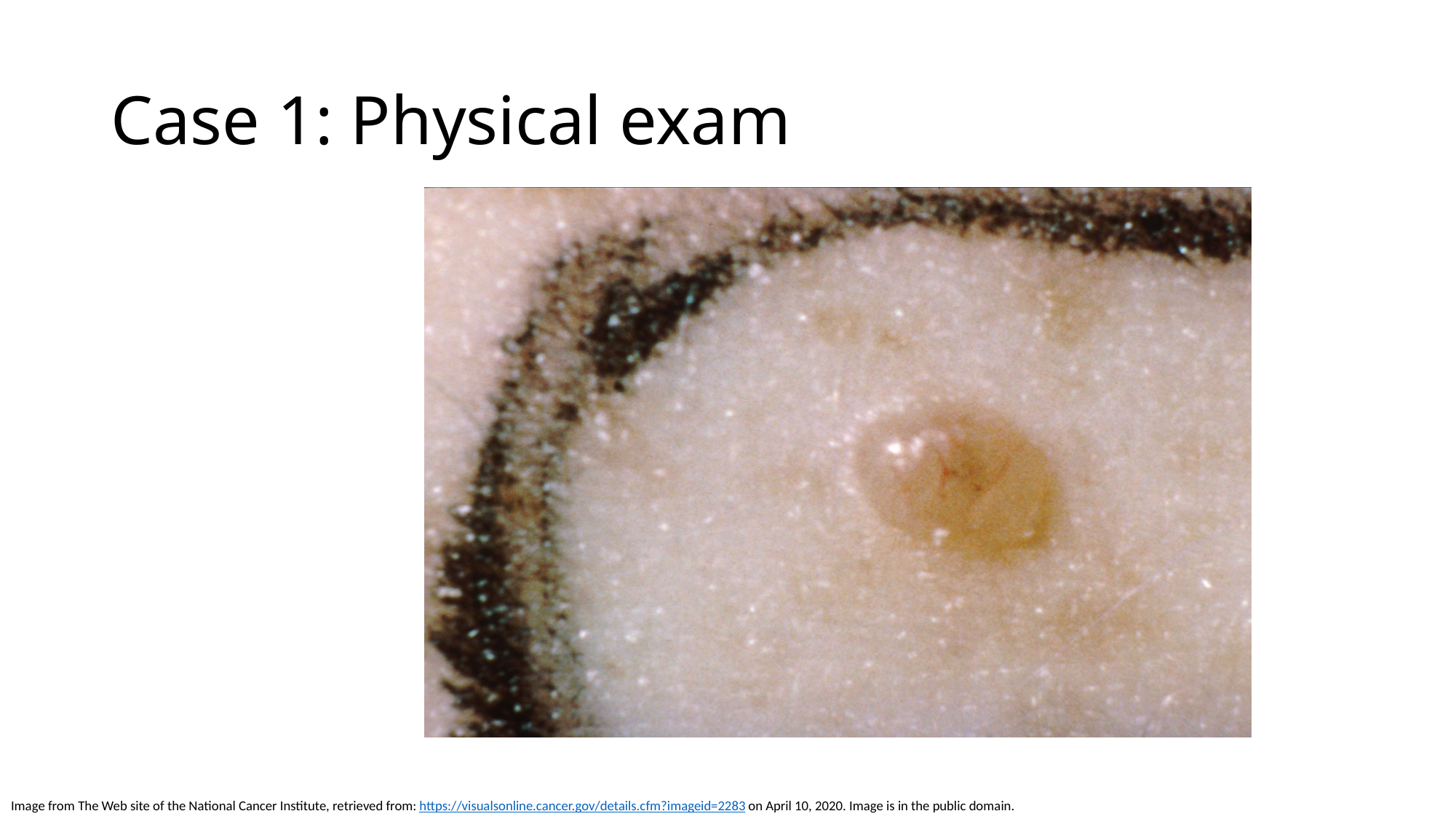

# Case 1: Physical exam
Image from The Web site of the National Cancer Institute, retrieved from: https://visualsonline.cancer.gov/details.cfm?imageid=2283 on April 10, 2020. Image is in the public domain.

## Slide 10
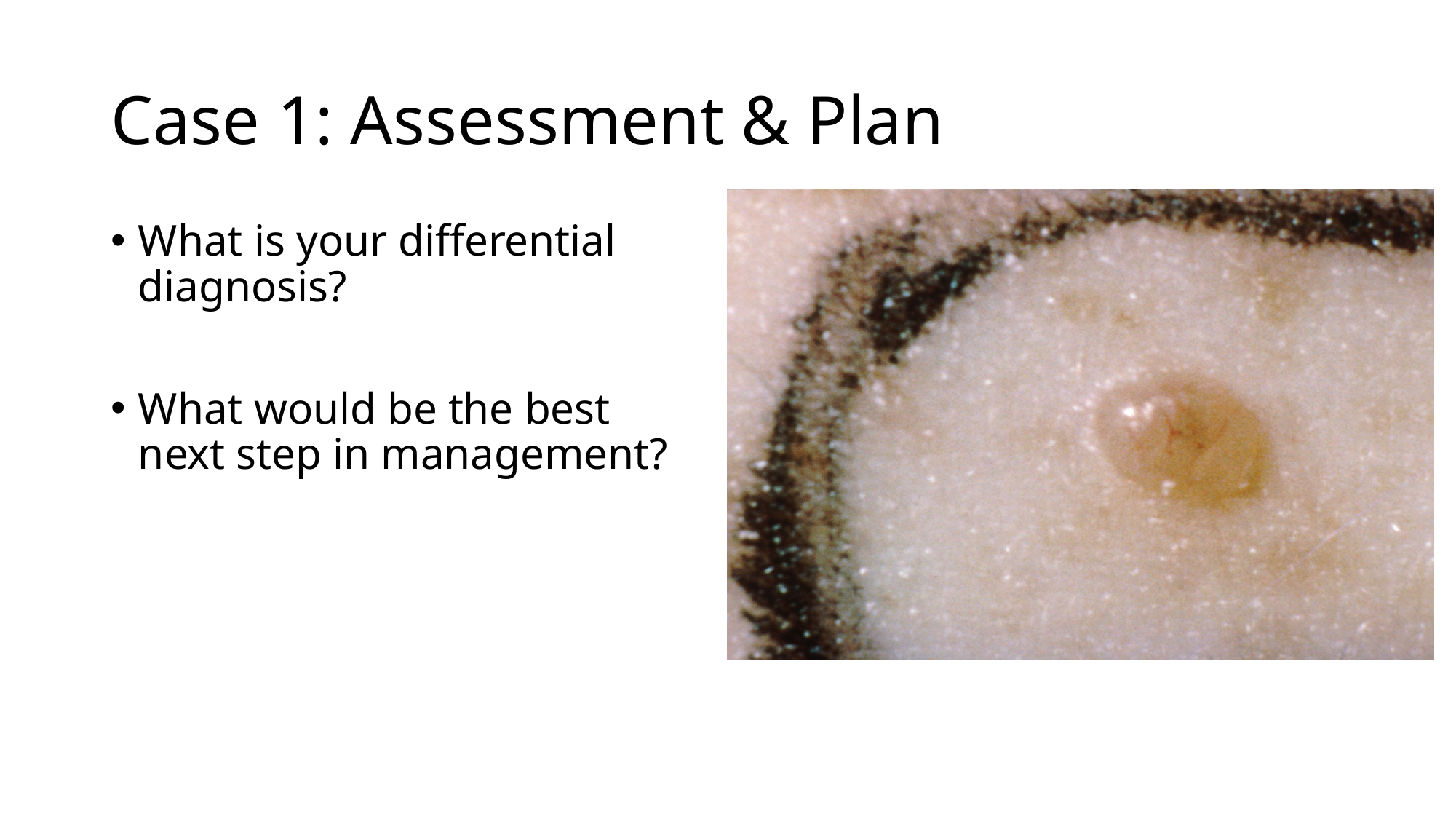

# Case 1: Assessment & Plan
What is your differential diagnosis?
What would be the best next step in management?

## Slide 11
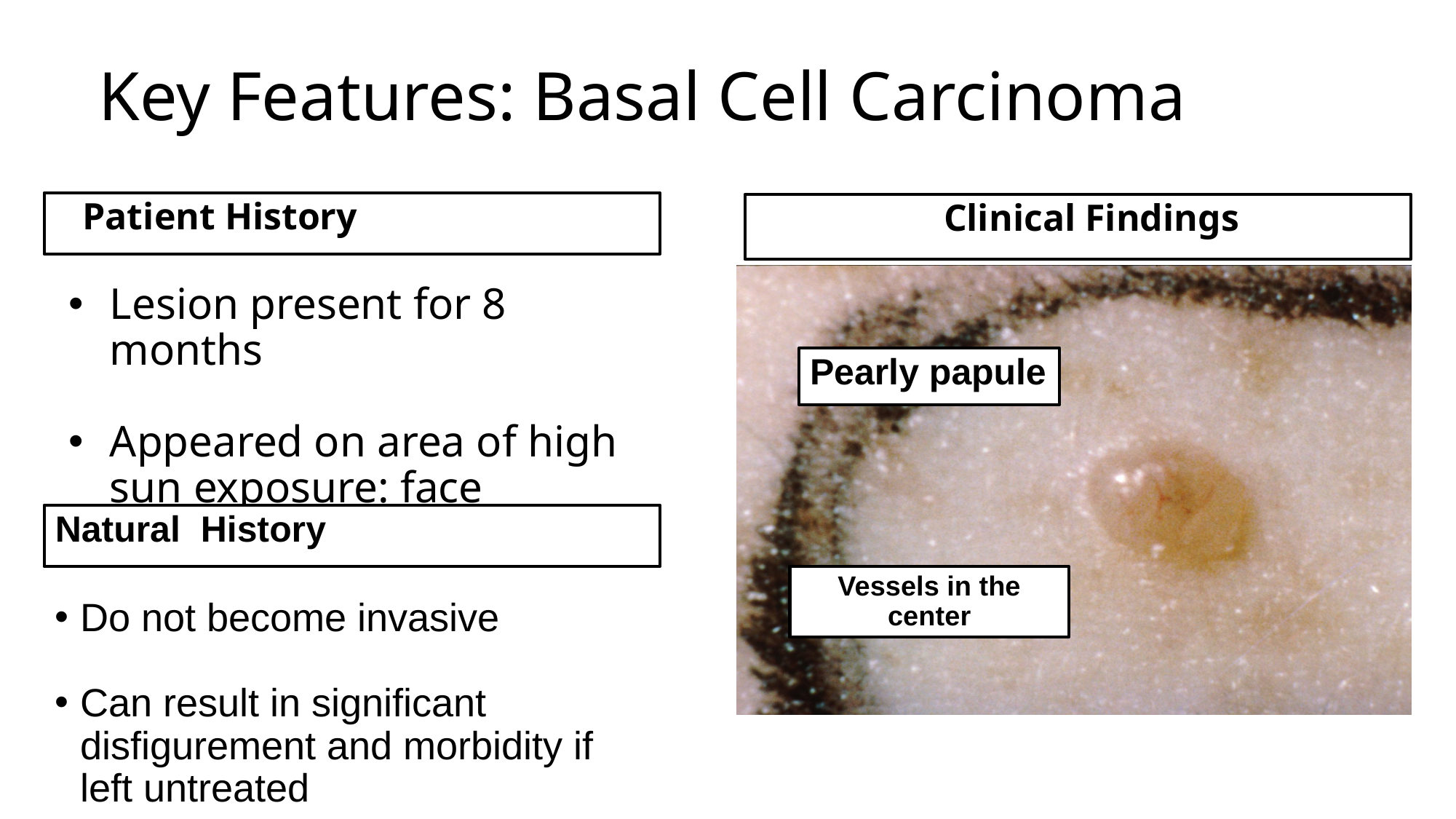

# Key Features: Basal Cell Carcinoma
Patient History
Clinical Findings
Lesion present for 8 months
Appeared on area of high sun exposure: face
Pearly papule
Natural History
Vessels in the center
Do not become invasive
Can result in significant disfigurement and morbidity if left untreated

## Slide 12
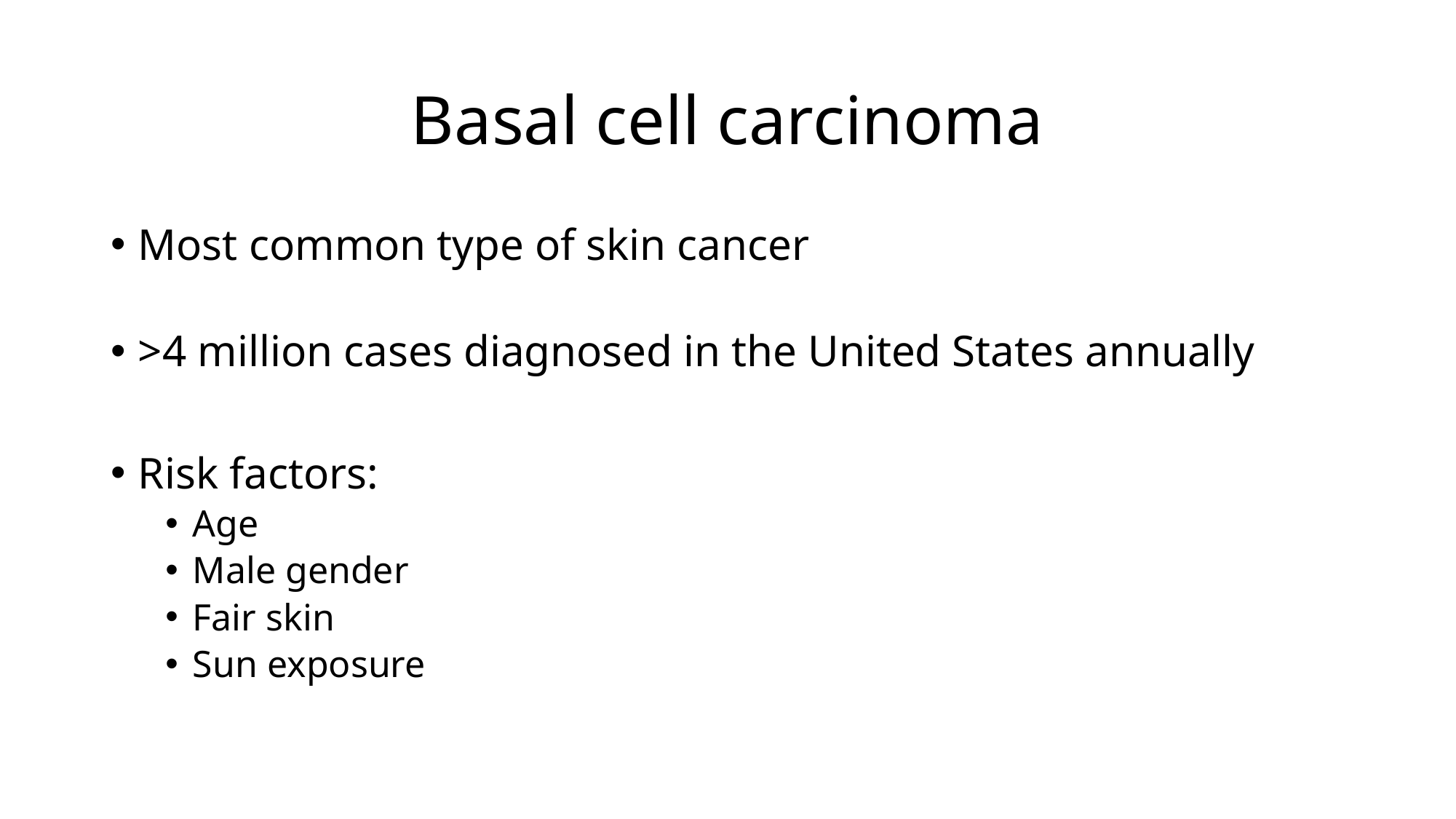

# Basal cell carcinoma
Most common type of skin cancer
>4 million cases diagnosed in the United States annually
Risk factors:
Age
Male gender
Fair skin
Sun exposure

## Slide 13
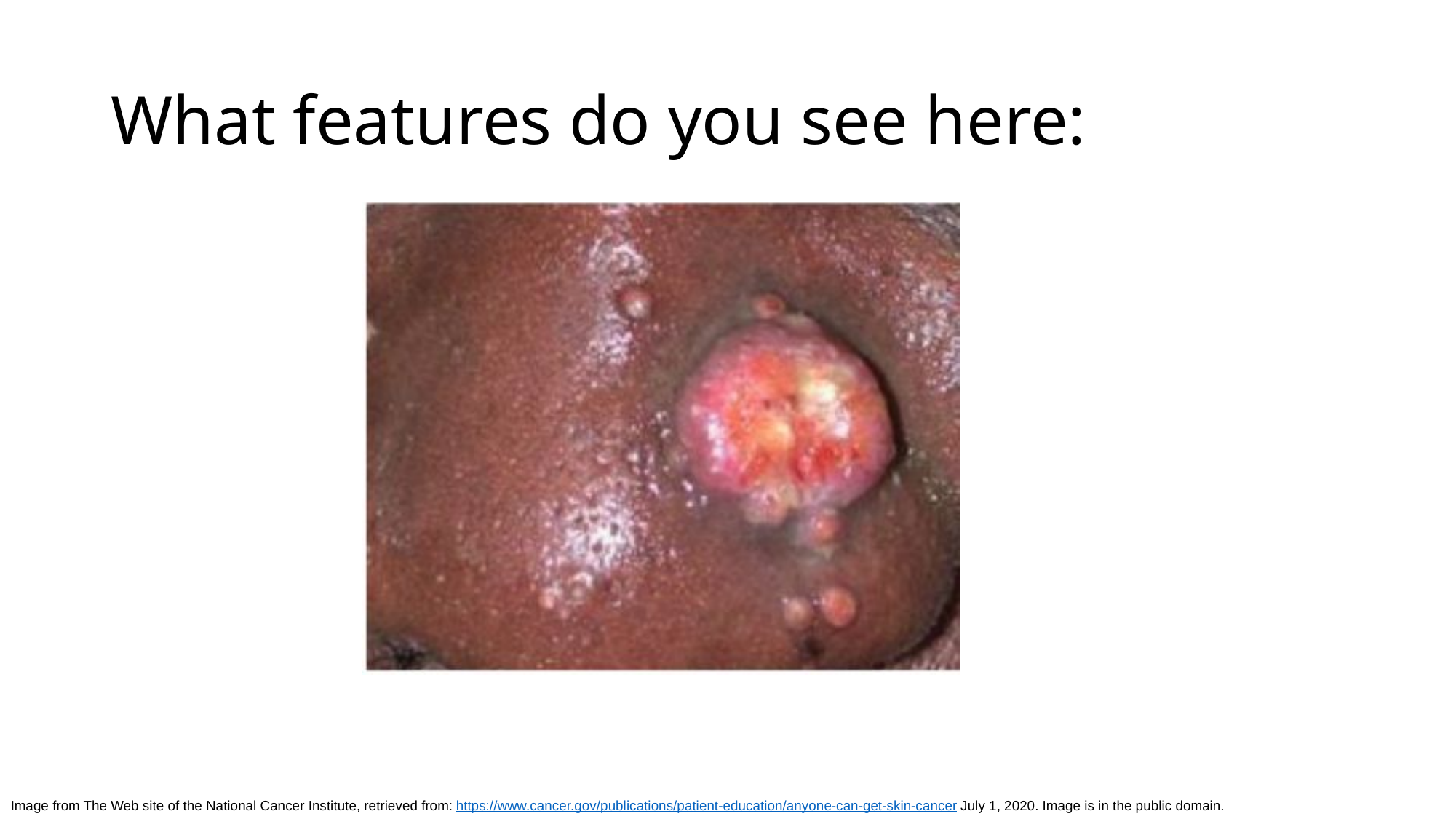

# What features do you see here:
Image from The Web site of the National Cancer Institute, retrieved from: https://www.cancer.gov/publications/patient-education/anyone-can-get-skin-cancer July 1, 2020. Image is in the public domain.

## Slide 14
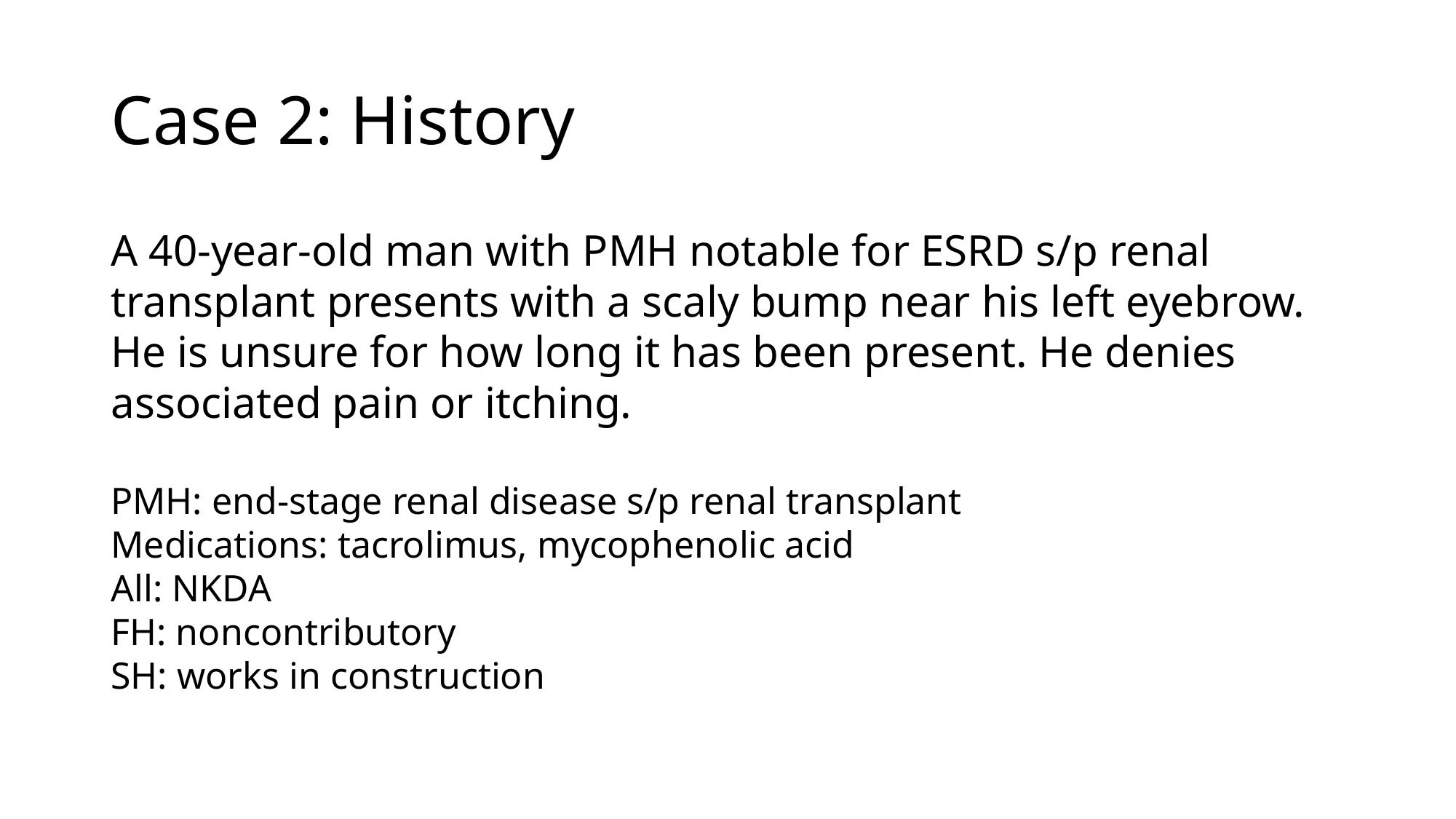

# Case 2: History
A 40-year-old man with PMH notable for ESRD s/p renal transplant presents with a scaly bump near his left eyebrow. He is unsure for how long it has been present. He denies associated pain or itching.
PMH: end-stage renal disease s/p renal transplant
Medications: tacrolimus, mycophenolic acid
All: NKDA
FH: noncontributory
SH: works in construction

## Slide 15
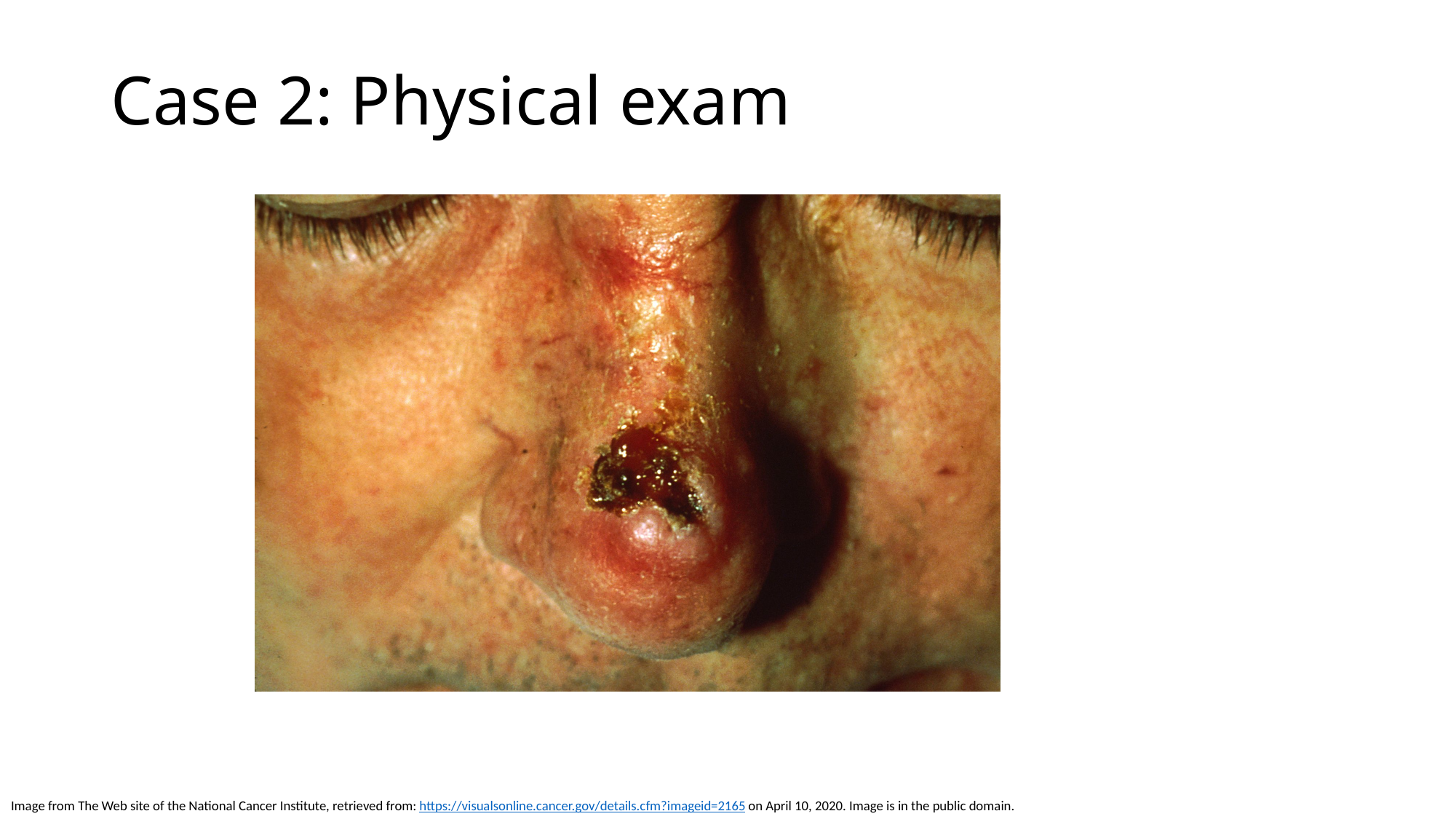

# Case 2: Physical exam
Image from The Web site of the National Cancer Institute, retrieved from: https://visualsonline.cancer.gov/details.cfm?imageid=2165 on April 10, 2020. Image is in the public domain.

## Slide 16
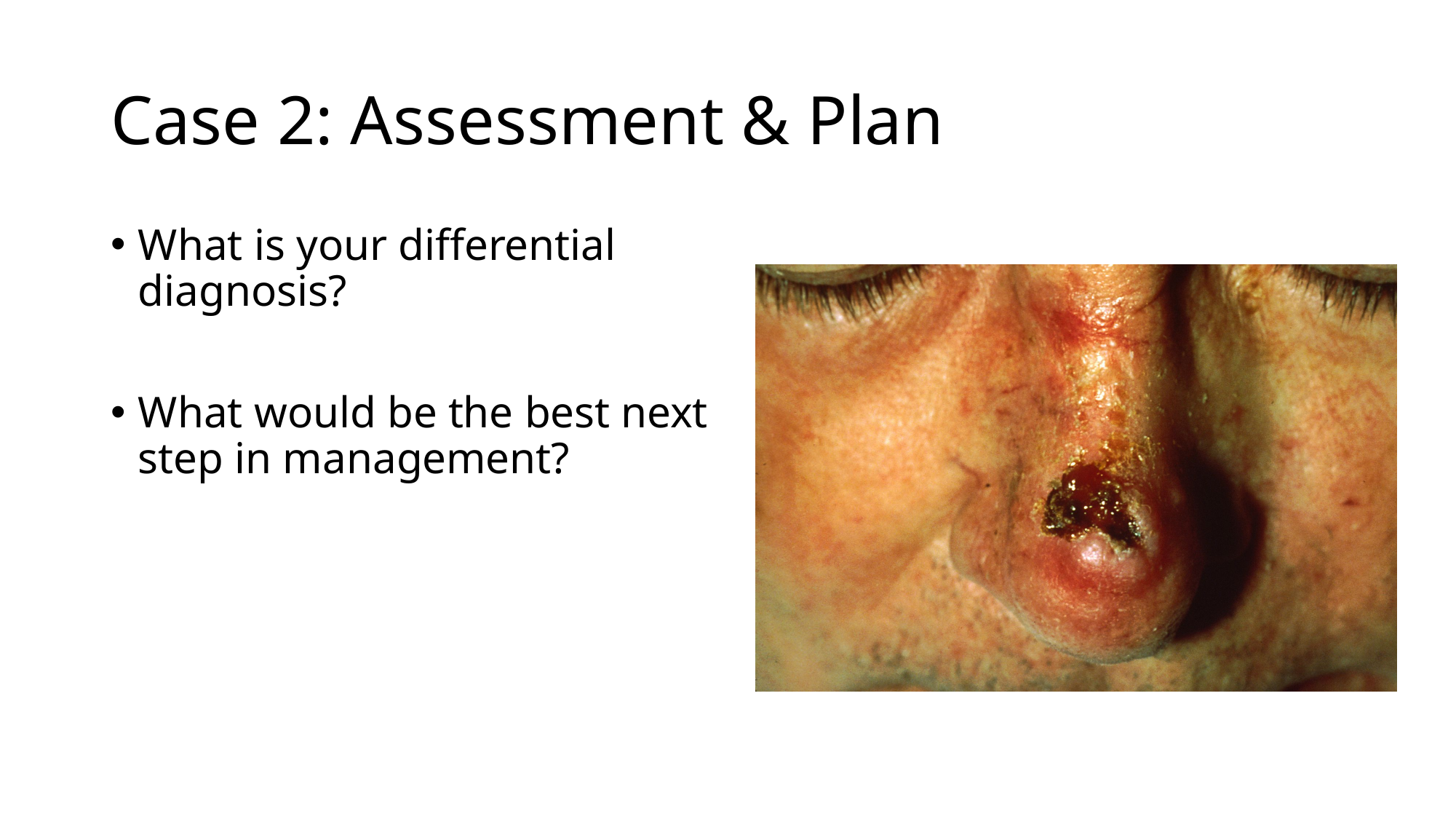

# Case 2: Assessment & Plan
What is your differential diagnosis?
What would be the best next step in management?

## Slide 17
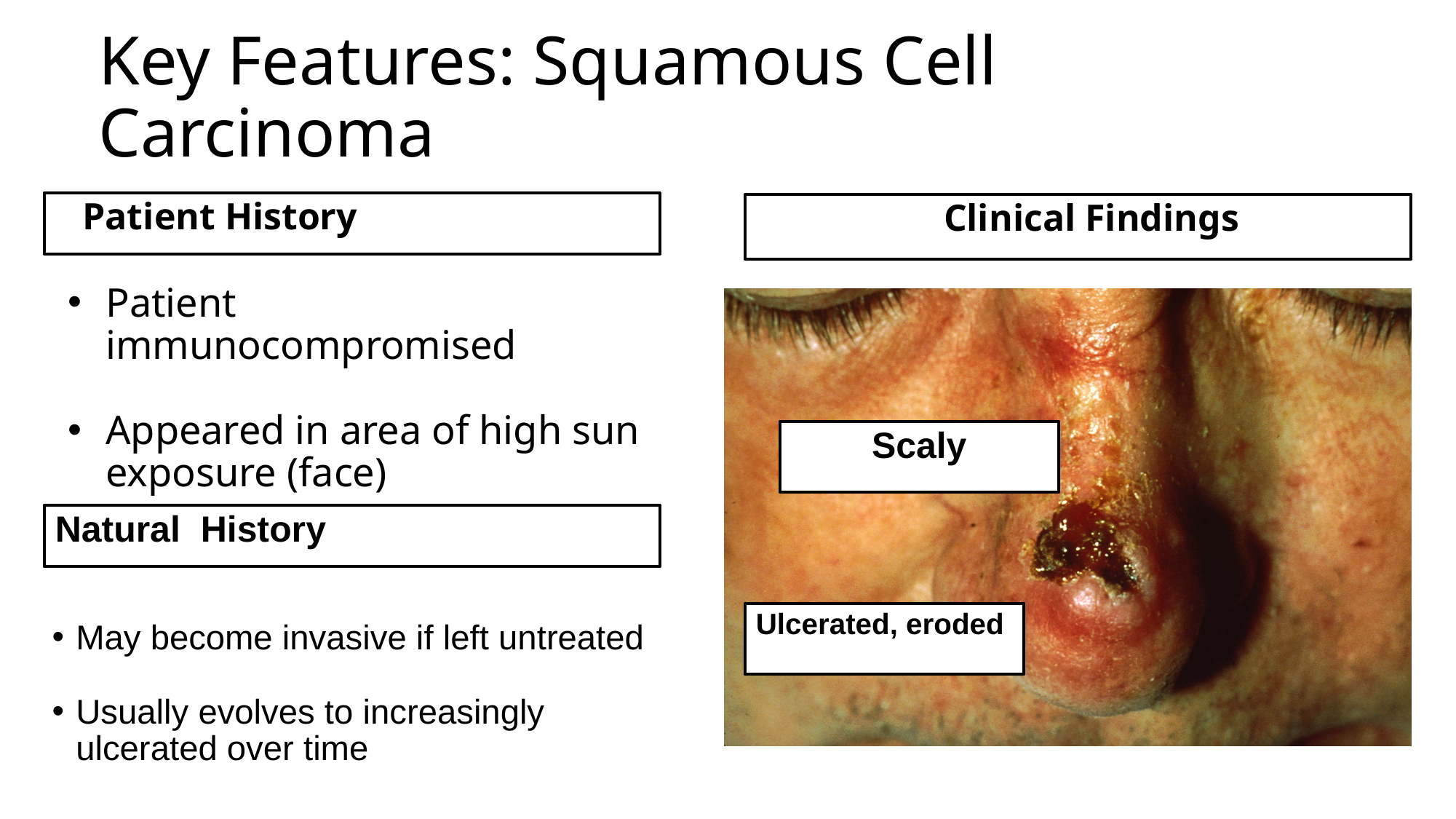

# Key Features: Squamous Cell Carcinoma
Patient History
Clinical Findings
Patient immunocompromised
Appeared in area of high sun exposure (face)
Scaly
Natural History
May become invasive if left untreated
Usually evolves to increasingly ulcerated over time
Ulcerated, eroded

## Slide 18
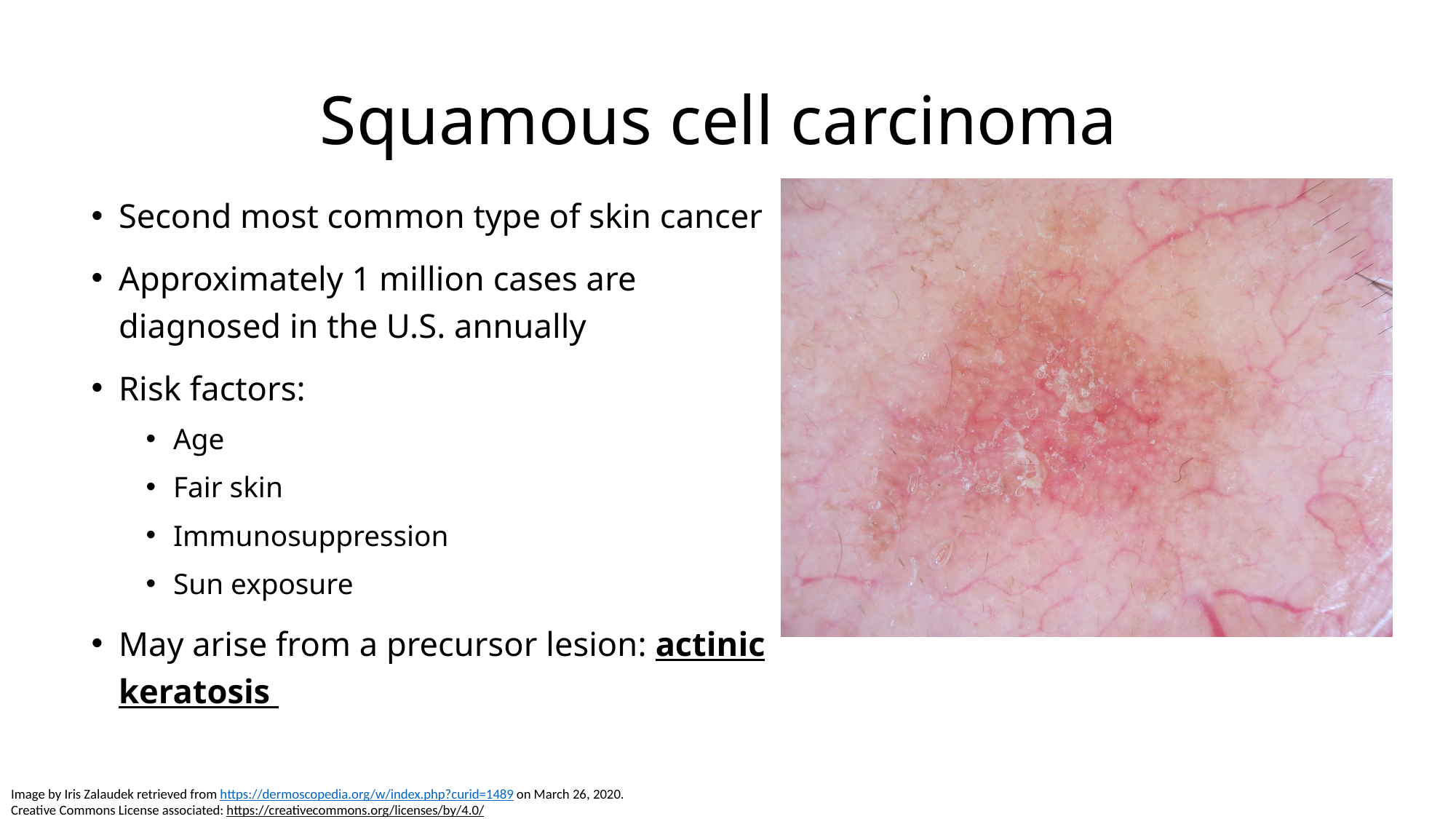

# Squamous cell carcinoma
Second most common type of skin cancer
Approximately 1 million cases are diagnosed in the U.S. annually
Risk factors:
Age
Fair skin
Immunosuppression
Sun exposure
May arise from a precursor lesion: actinic keratosis
Image by Iris Zalaudek retrieved from https://dermoscopedia.org/w/index.php?curid=1489 on March 26, 2020.
Creative Commons License associated: https://creativecommons.org/licenses/by/4.0/

## Slide 19
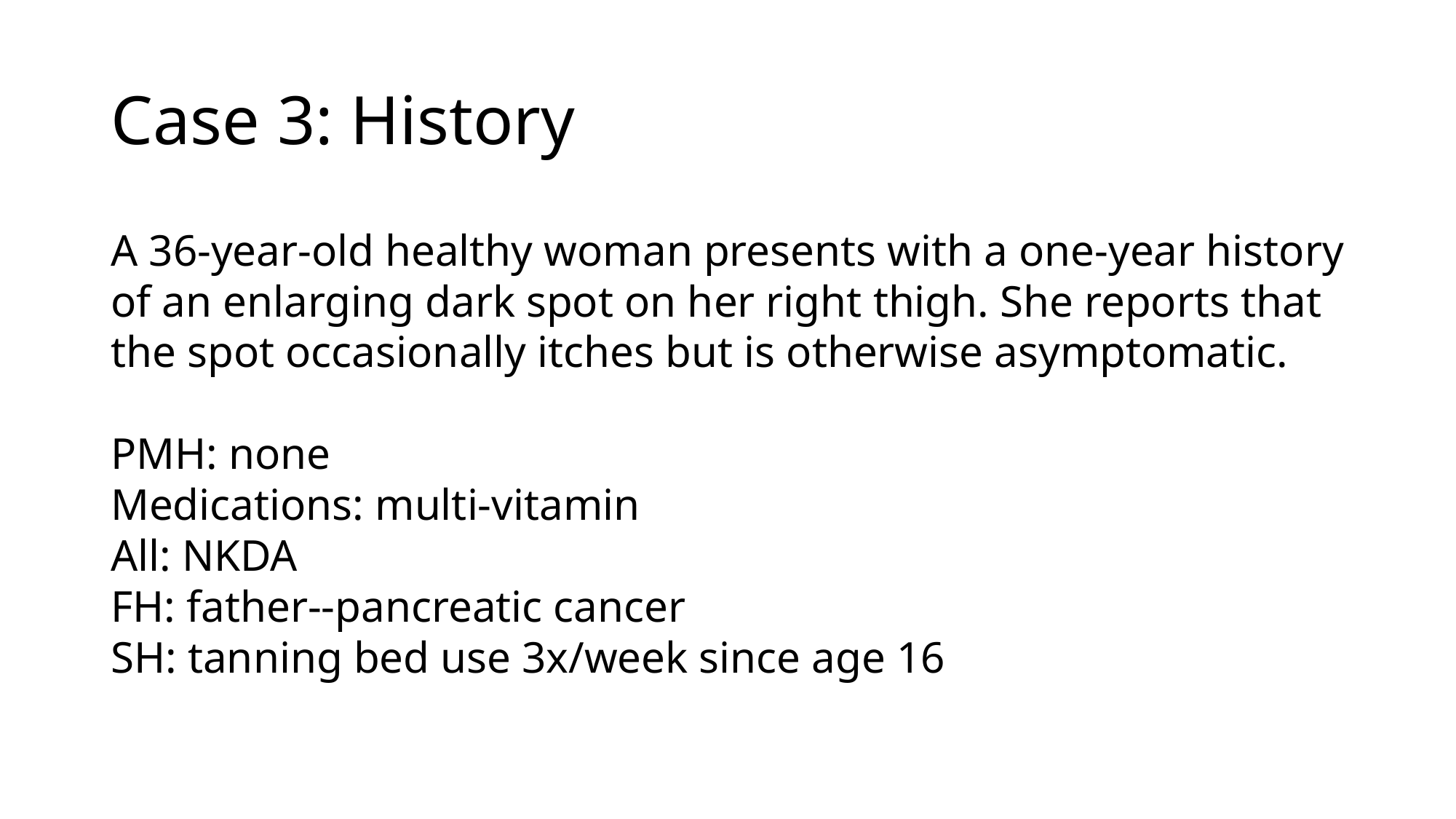

# Case 3: History
A 36-year-old healthy woman presents with a one-year history of an enlarging dark spot on her right thigh. She reports that the spot occasionally itches but is otherwise asymptomatic.
PMH: none
Medications: multi-vitamin
All: NKDA
FH: father--pancreatic cancer
SH: tanning bed use 3x/week since age 16

## Slide 20
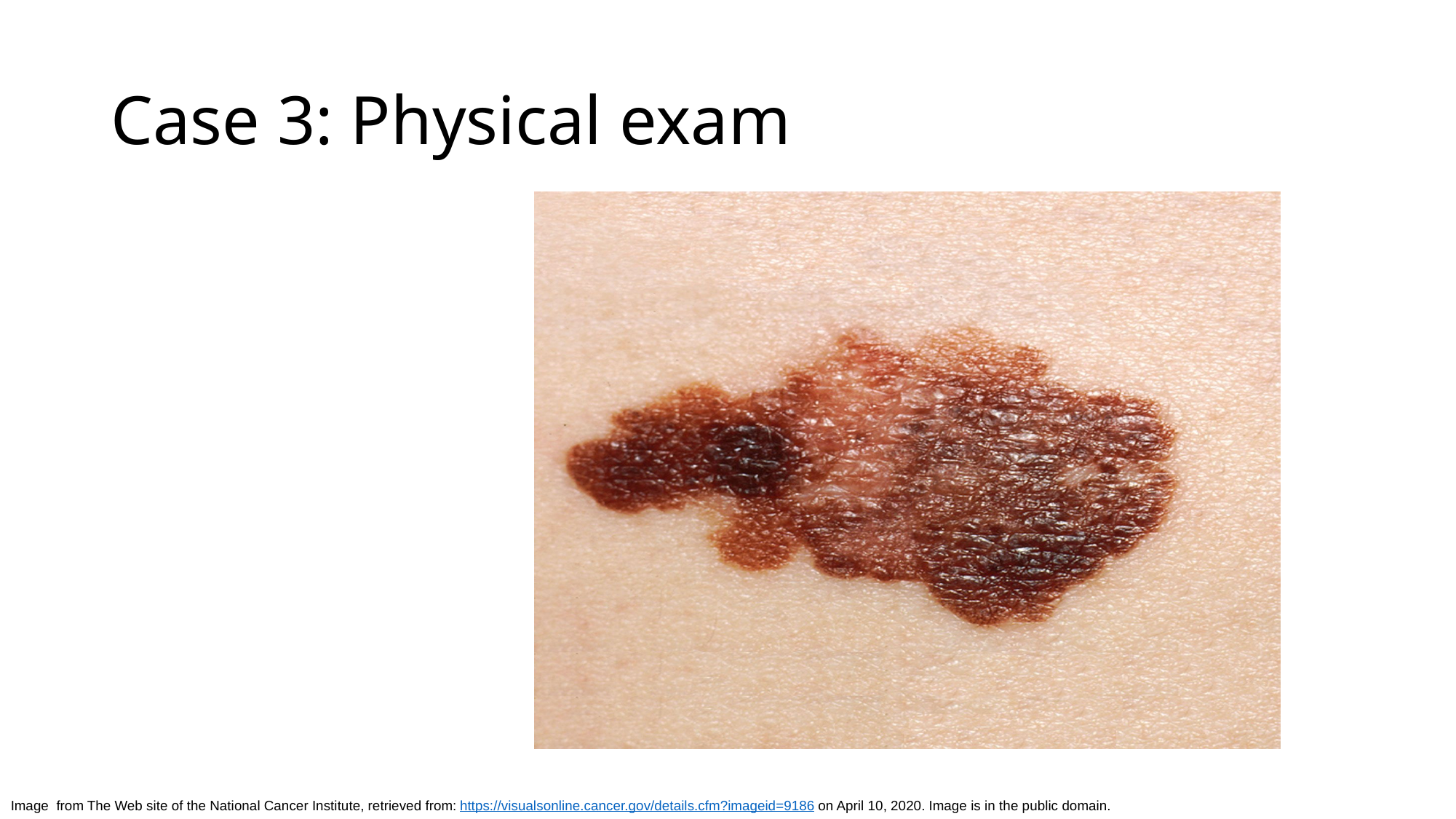

# Case 3: Physical exam
Image from The Web site of the National Cancer Institute, retrieved from: https://visualsonline.cancer.gov/details.cfm?imageid=9186 on April 10, 2020. Image is in the public domain.

## Slide 21
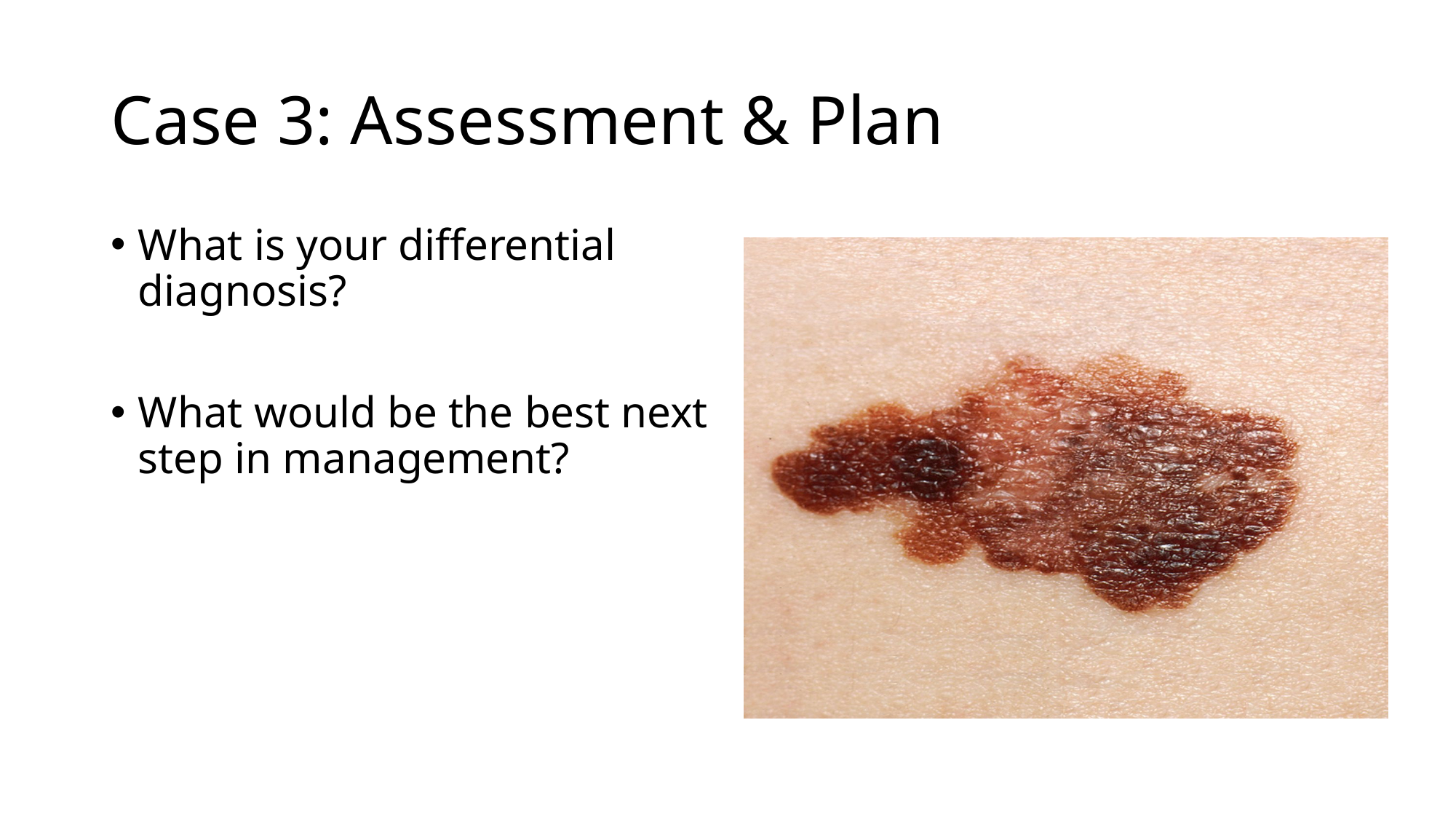

# Case 3: Assessment & Plan
What is your differential diagnosis?
What would be the best next step in management?

## Slide 22
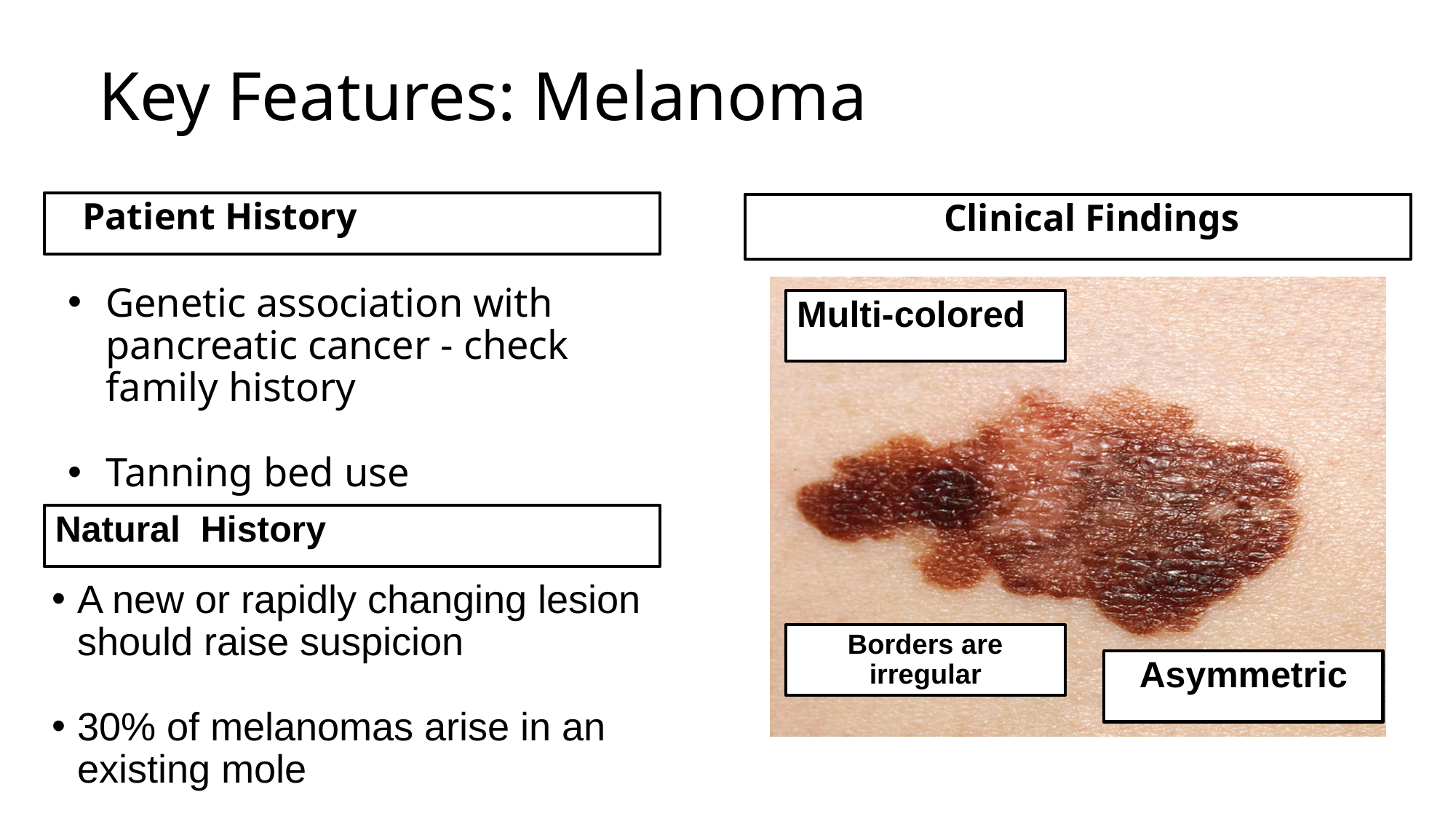

# Key Features: Melanoma
Patient History
Clinical Findings
Genetic association with pancreatic cancer - check family history
Tanning bed use
Multi-colored
Natural History
A new or rapidly changing lesion should raise suspicion
30% of melanomas arise in an existing mole
Borders are irregular
Asymmetric

## Slide 23
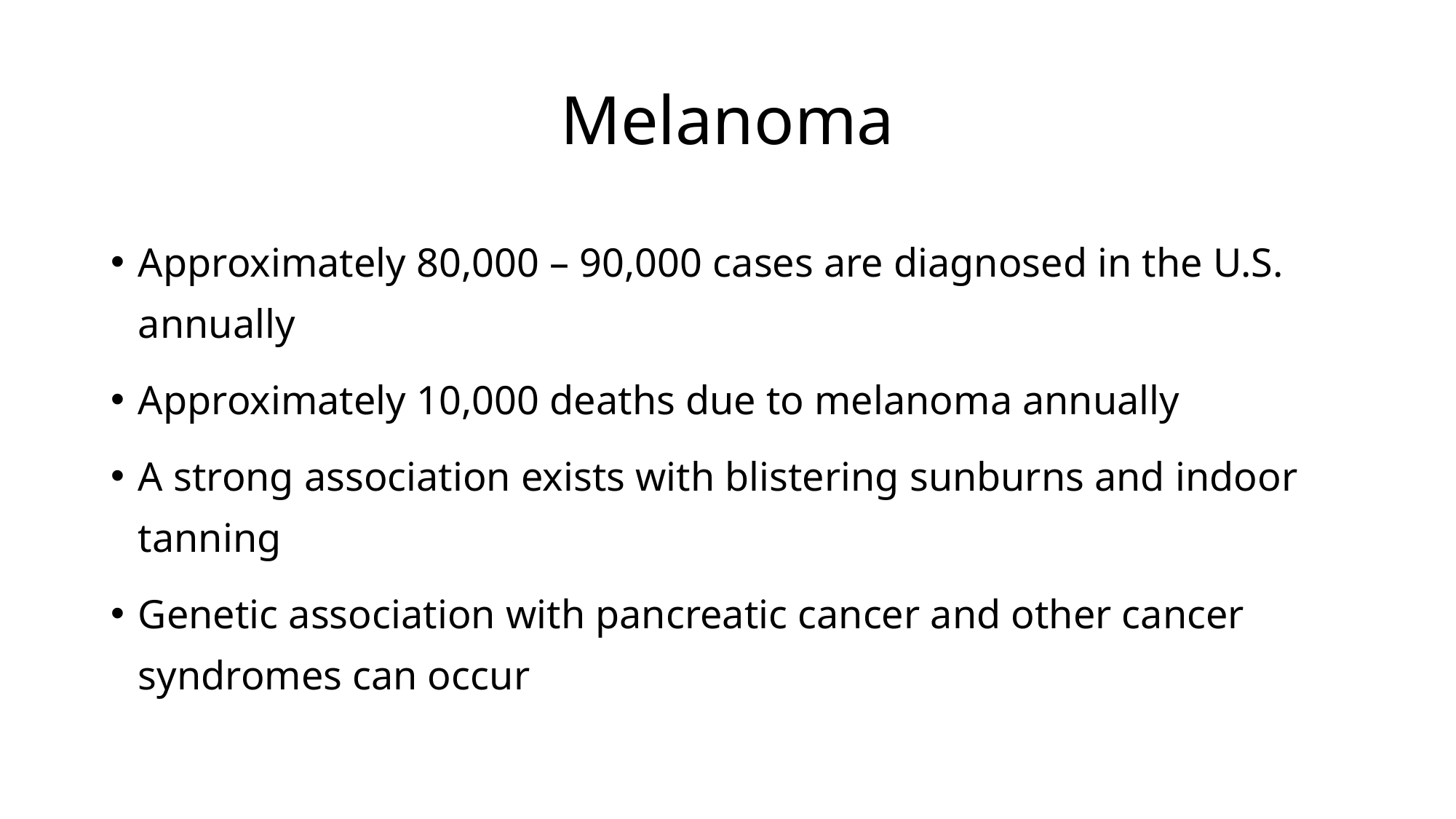

# Melanoma
Approximately 80,000 – 90,000 cases are diagnosed in the U.S. annually
Approximately 10,000 deaths due to melanoma annually
A strong association exists with blistering sunburns and indoor tanning
Genetic association with pancreatic cancer and other cancer syndromes can occur

## Slide 24
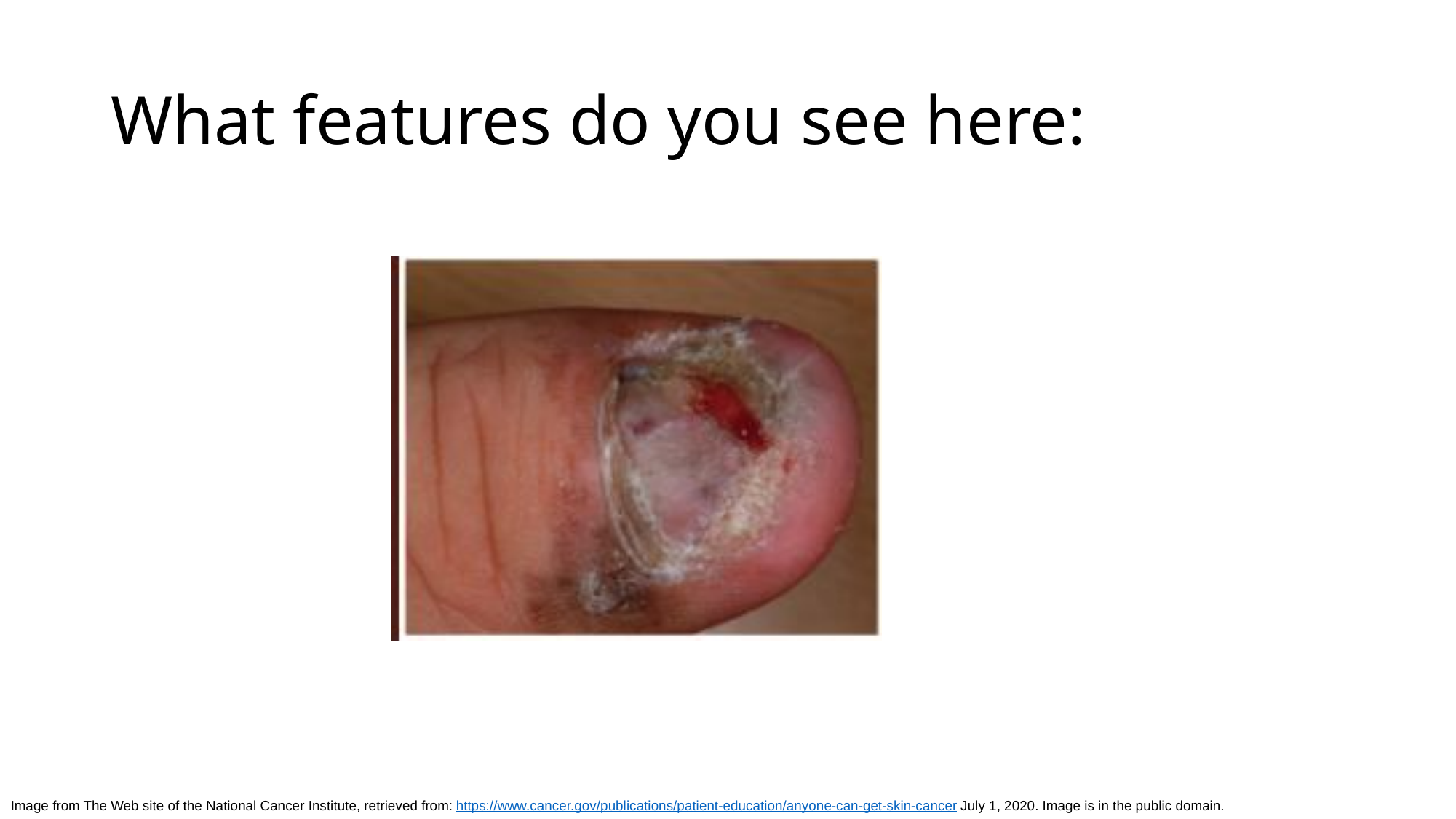

# What features do you see here:
Image from The Web site of the National Cancer Institute, retrieved from: https://www.cancer.gov/publications/patient-education/anyone-can-get-skin-cancer July 1, 2020. Image is in the public domain.

## Slide 25
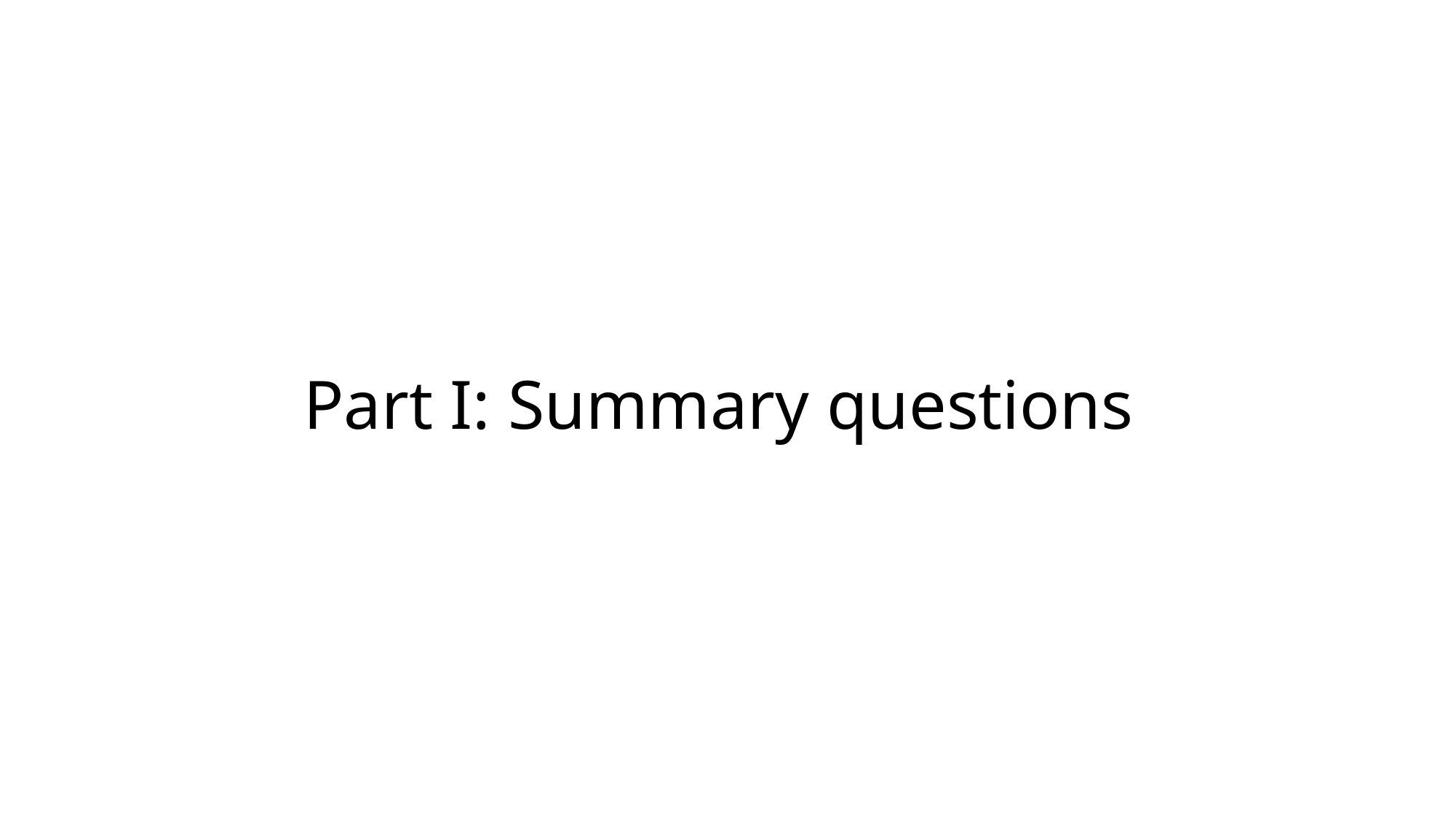

# Part I: Summary questions

## Slide 26
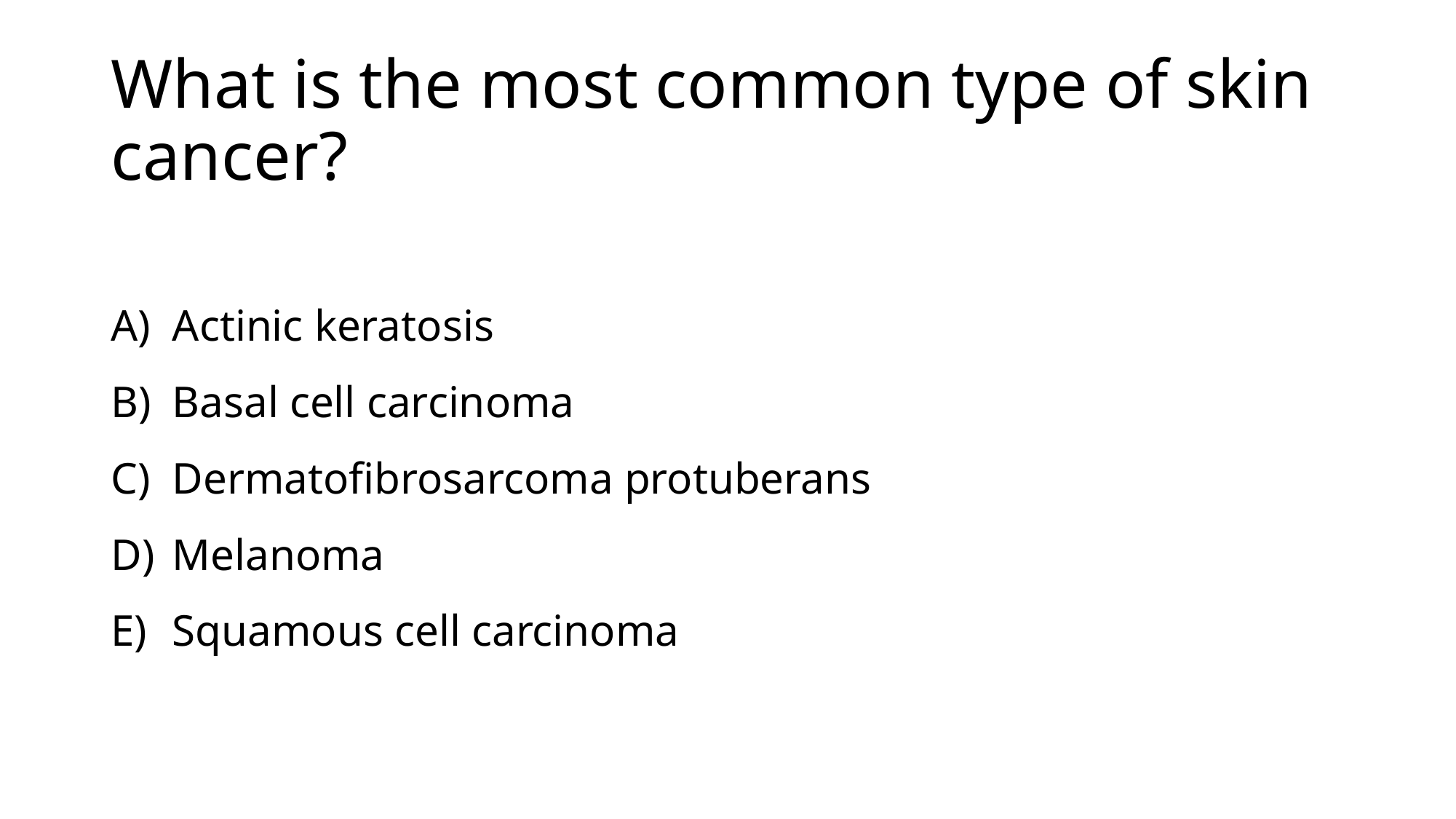

# What is the most common type of skin cancer?
Actinic keratosis
Basal cell carcinoma
Dermatofibrosarcoma protuberans
Melanoma
Squamous cell carcinoma

## Slide 27
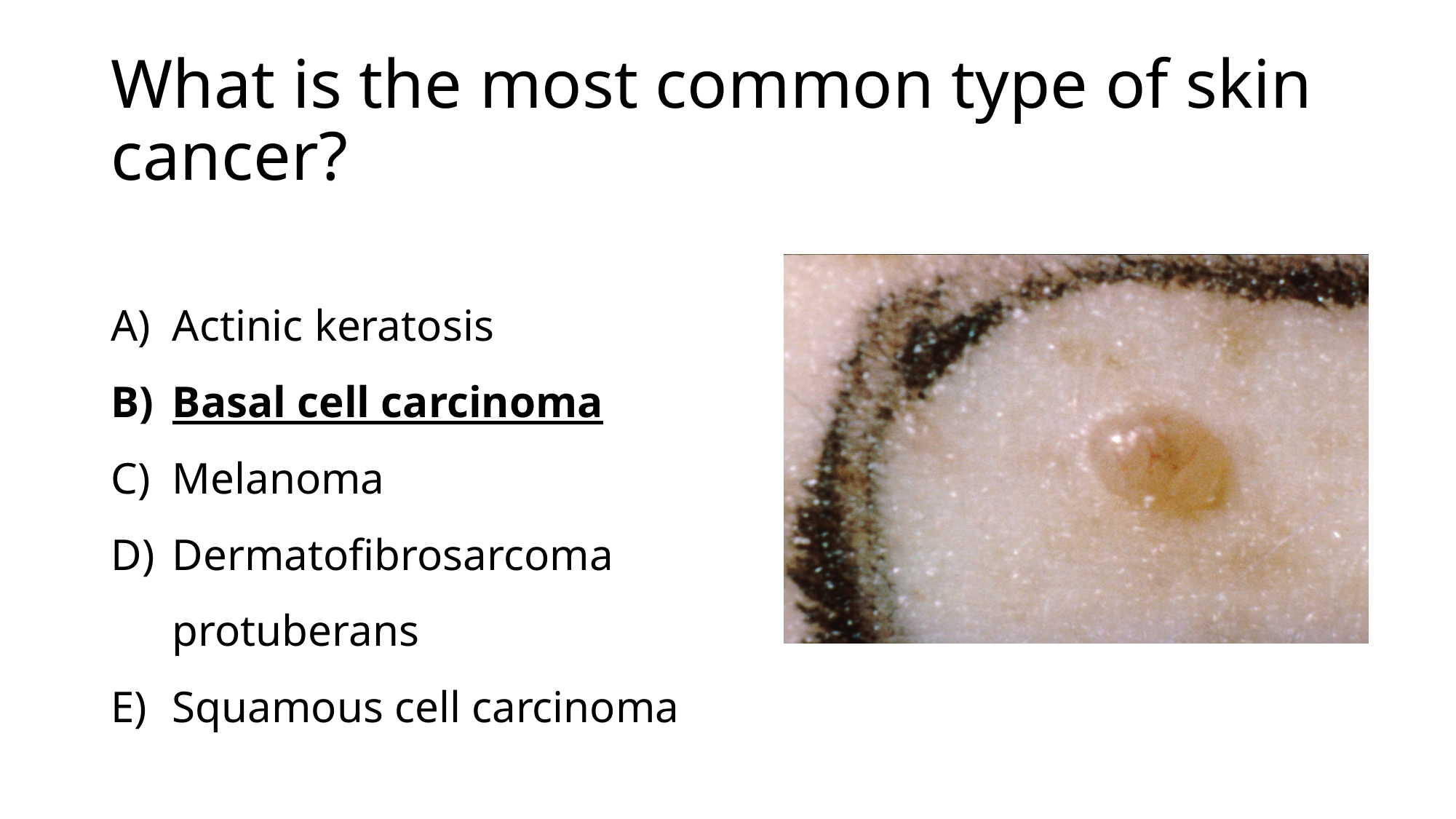

# What is the most common type of skin cancer?
Actinic keratosis
Basal cell carcinoma
Melanoma
Dermatofibrosarcoma protuberans
Squamous cell carcinoma

## Slide 28
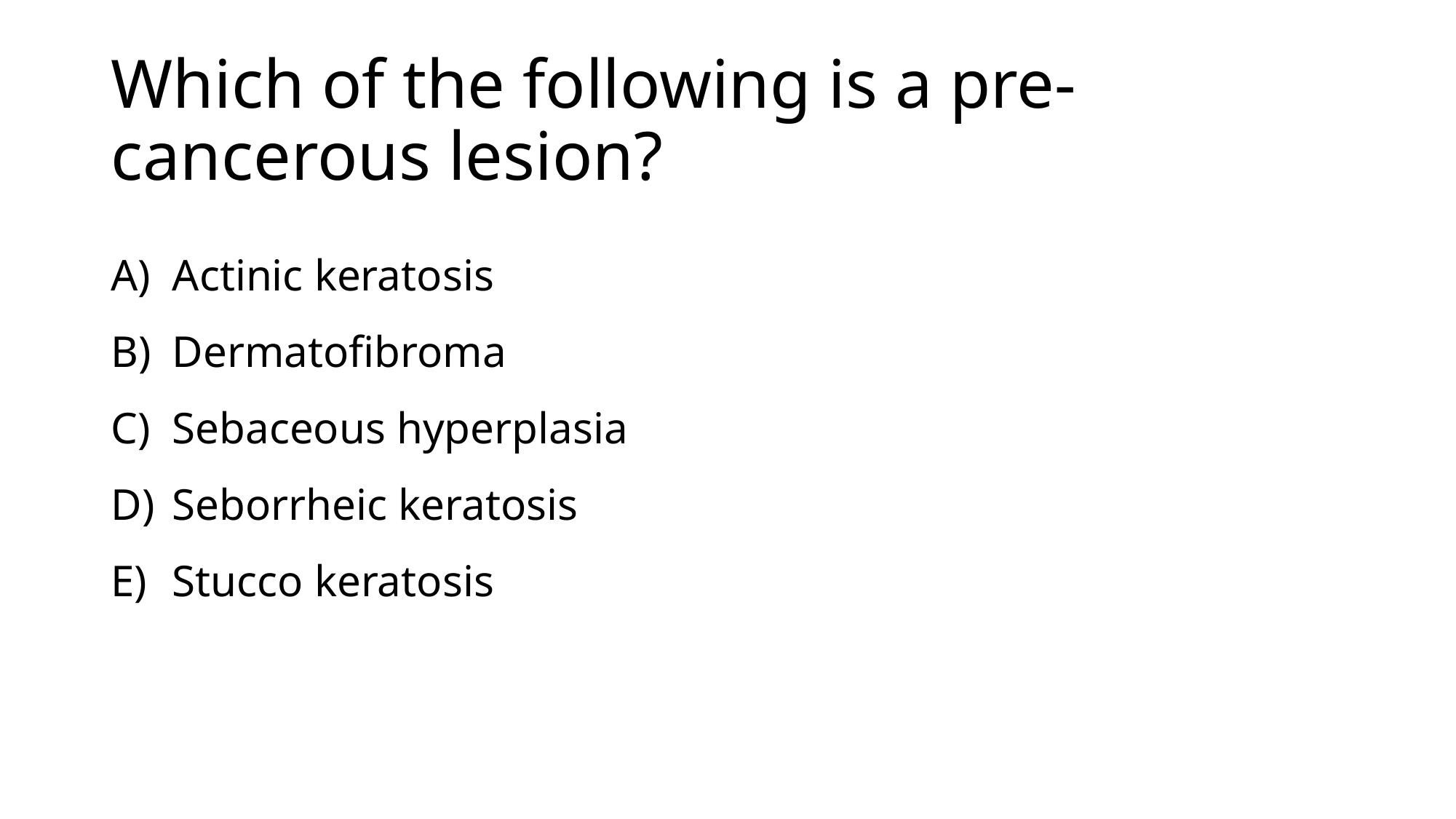

# Which of the following is a pre-cancerous lesion?
Actinic keratosis
Dermatofibroma
Sebaceous hyperplasia
Seborrheic keratosis
Stucco keratosis

## Slide 29
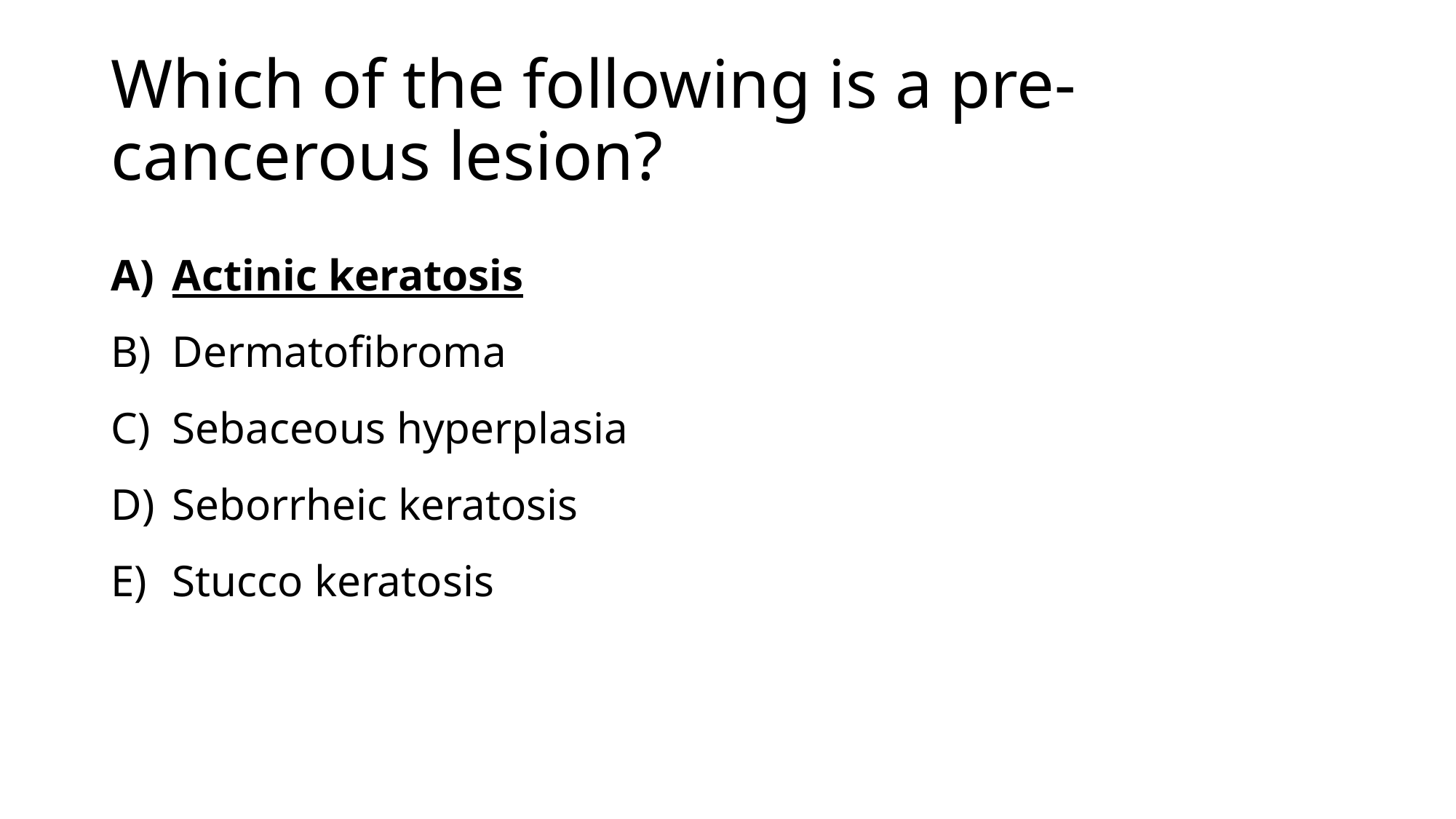

# Which of the following is a pre-cancerous lesion?
Actinic keratosis
Dermatofibroma
Sebaceous hyperplasia
Seborrheic keratosis
Stucco keratosis

## Slide 30
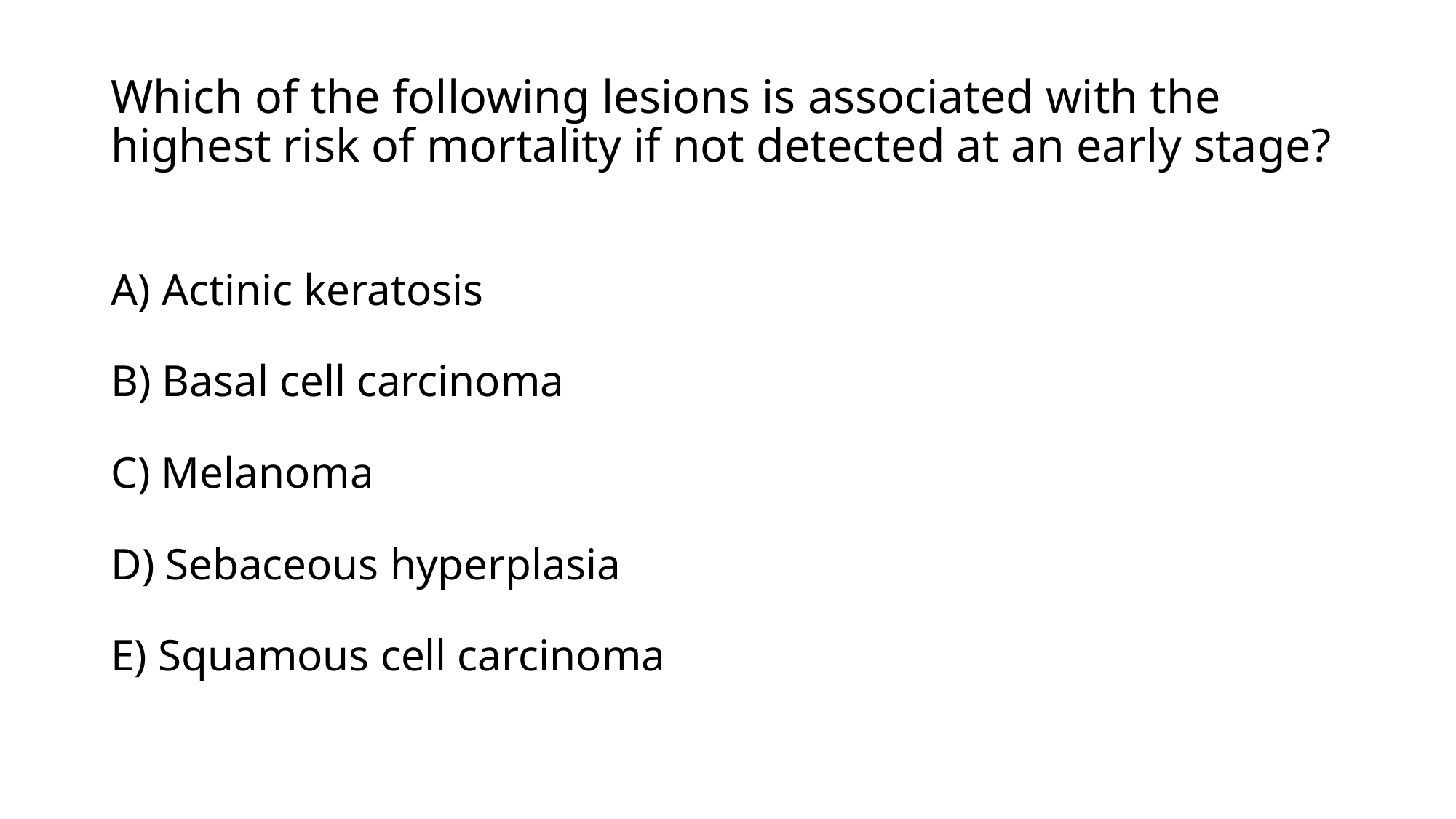

# Which of the following lesions is associated with the highest risk of mortality if not detected at an early stage?
A) Actinic keratosis
B) Basal cell carcinoma
C) Melanoma
D) Sebaceous hyperplasia
E) Squamous cell carcinoma

## Slide 31
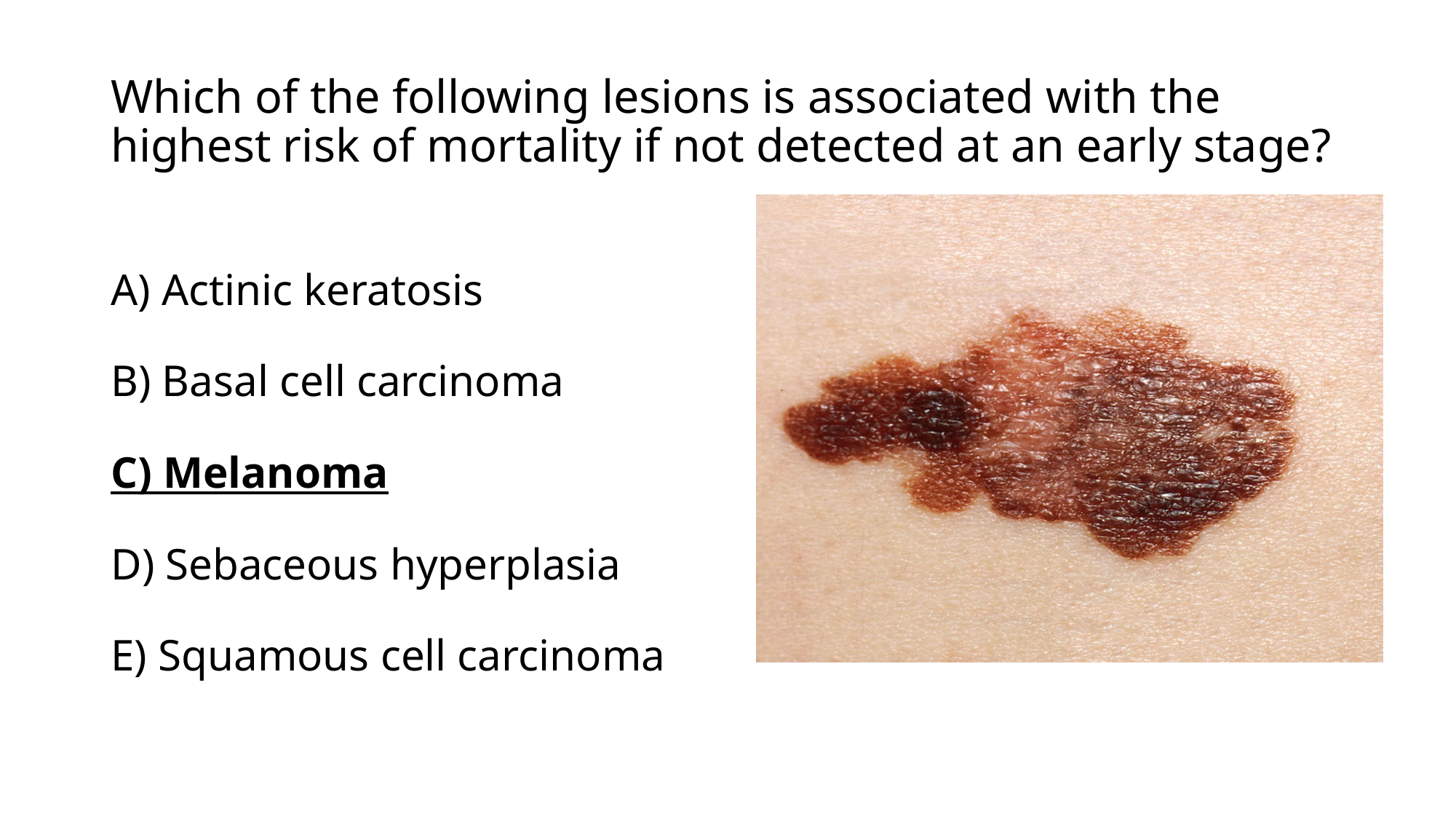

# Which of the following lesions is associated with the highest risk of mortality if not detected at an early stage?
A) Actinic keratosis
B) Basal cell carcinoma
C) Melanoma
D) Sebaceous hyperplasia
E) Squamous cell carcinoma

## Slide 32
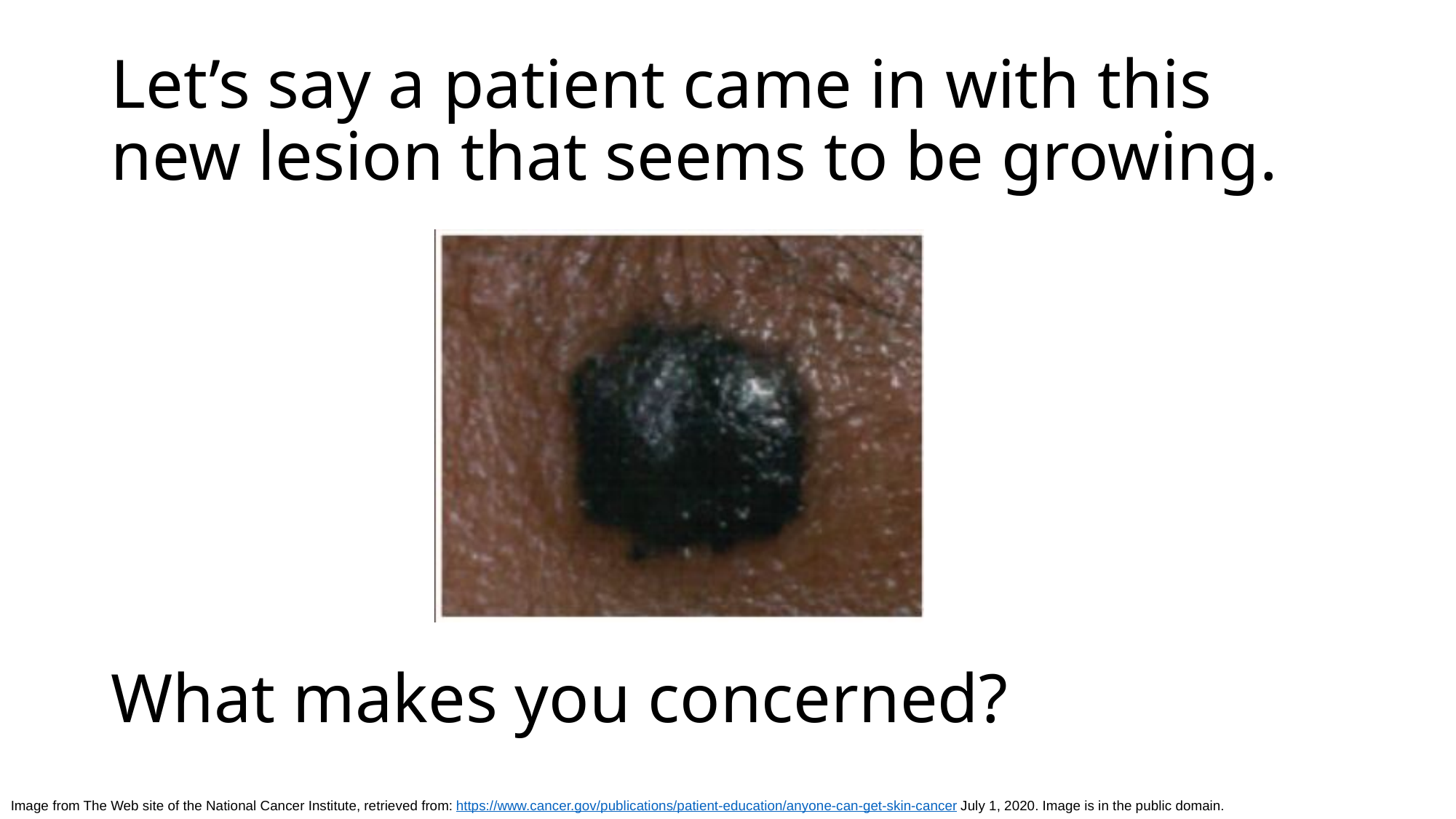

# Let’s say a patient came in with this new lesion that seems to be growing.
What makes you concerned?
Image from The Web site of the National Cancer Institute, retrieved from: https://www.cancer.gov/publications/patient-education/anyone-can-get-skin-cancer July 1, 2020. Image is in the public domain.

## Slide 33
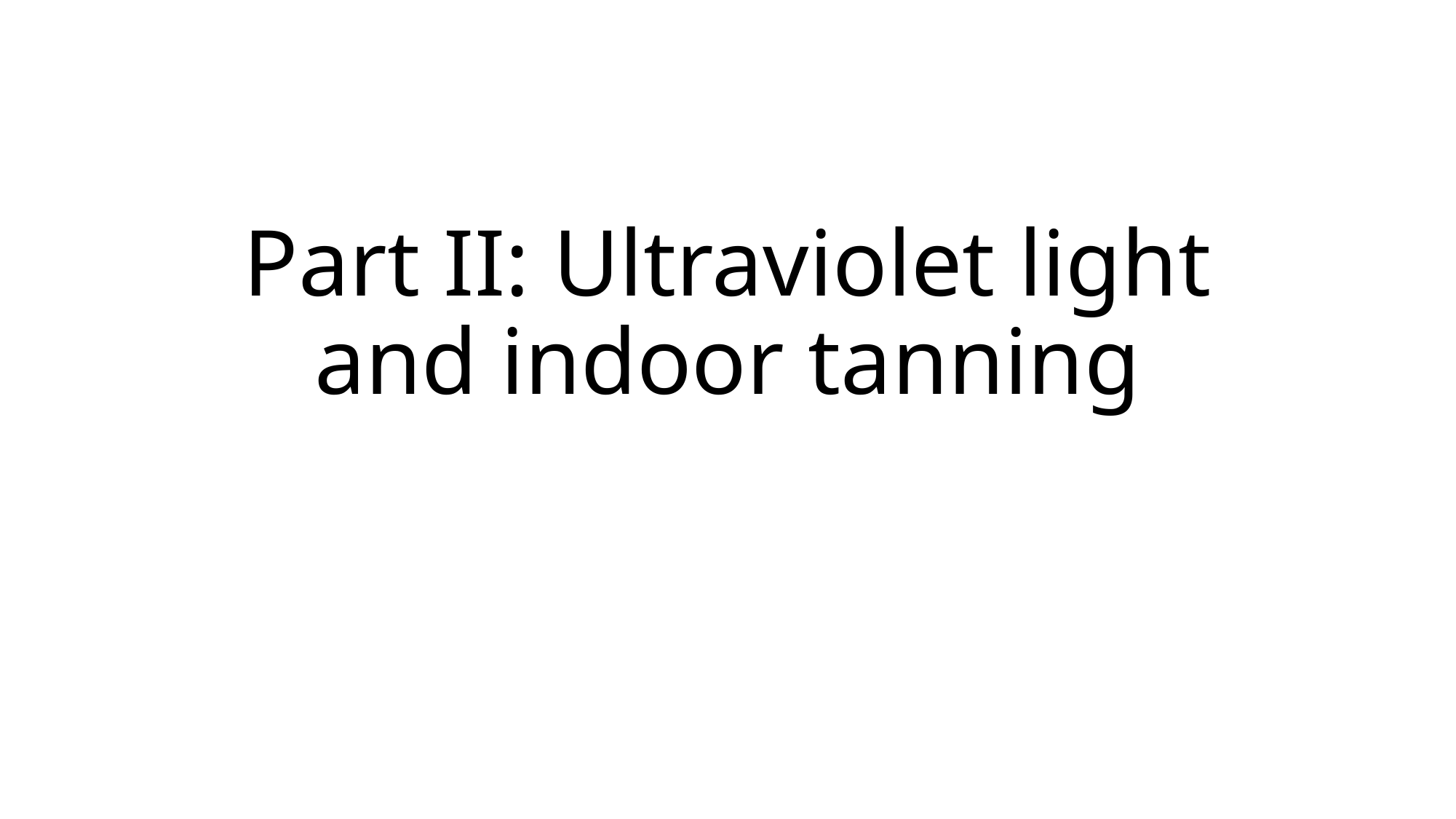

# Part II: Ultraviolet light and indoor tanning

## Slide 34
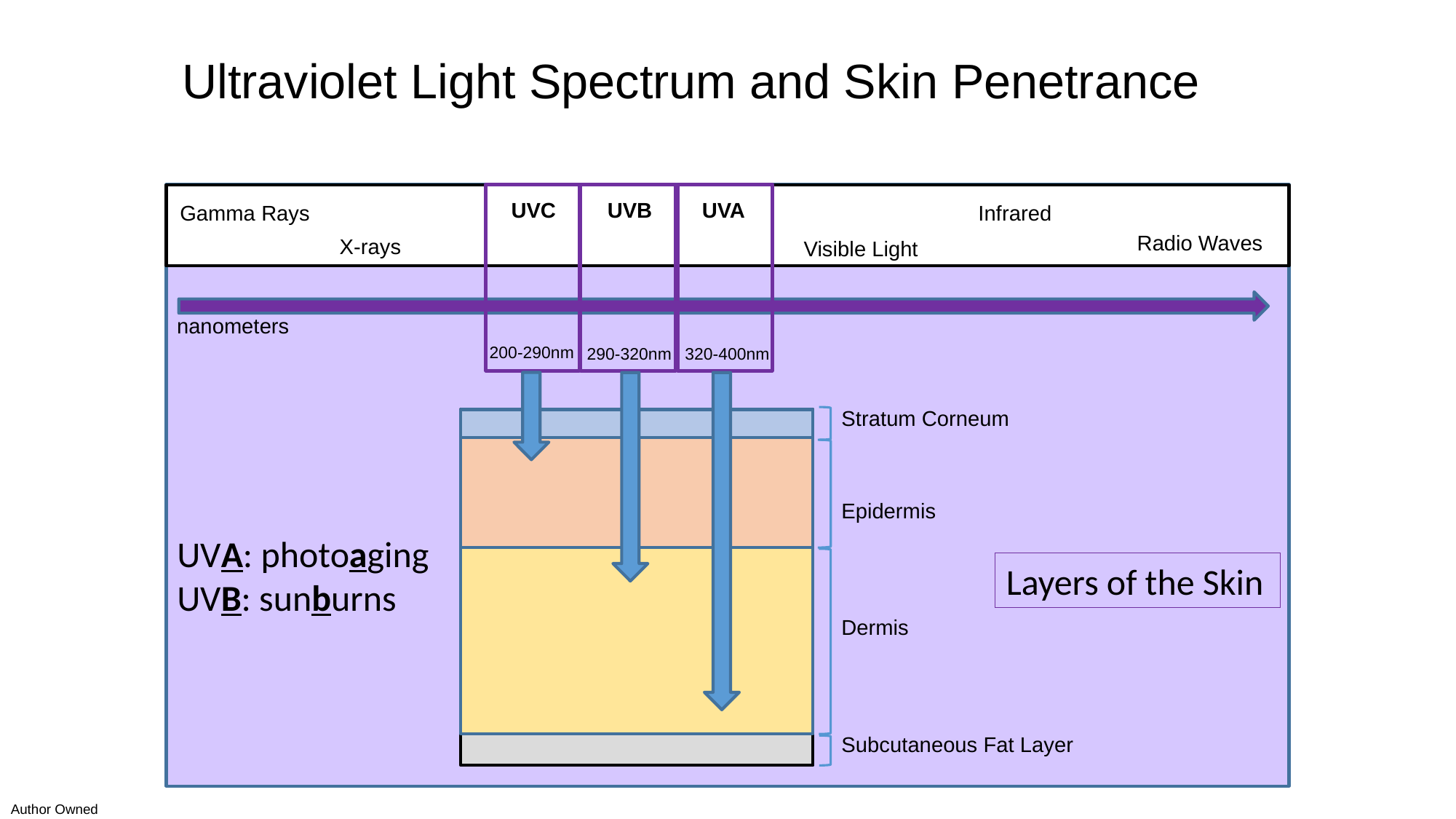

Ultraviolet Light Spectrum and Skin Penetrance
UVC
UVB
UVA
Infrared
Gamma Rays
Radio Waves
X-rays
Visible Light
nanometers
200-290nm
290-320nm
320-400nm
Stratum Corneum
Epidermis
UVA: photoaging
UVB: sunburns
Layers of the Skin
Dermis
Subcutaneous Fat Layer
Author Owned

## Slide 35
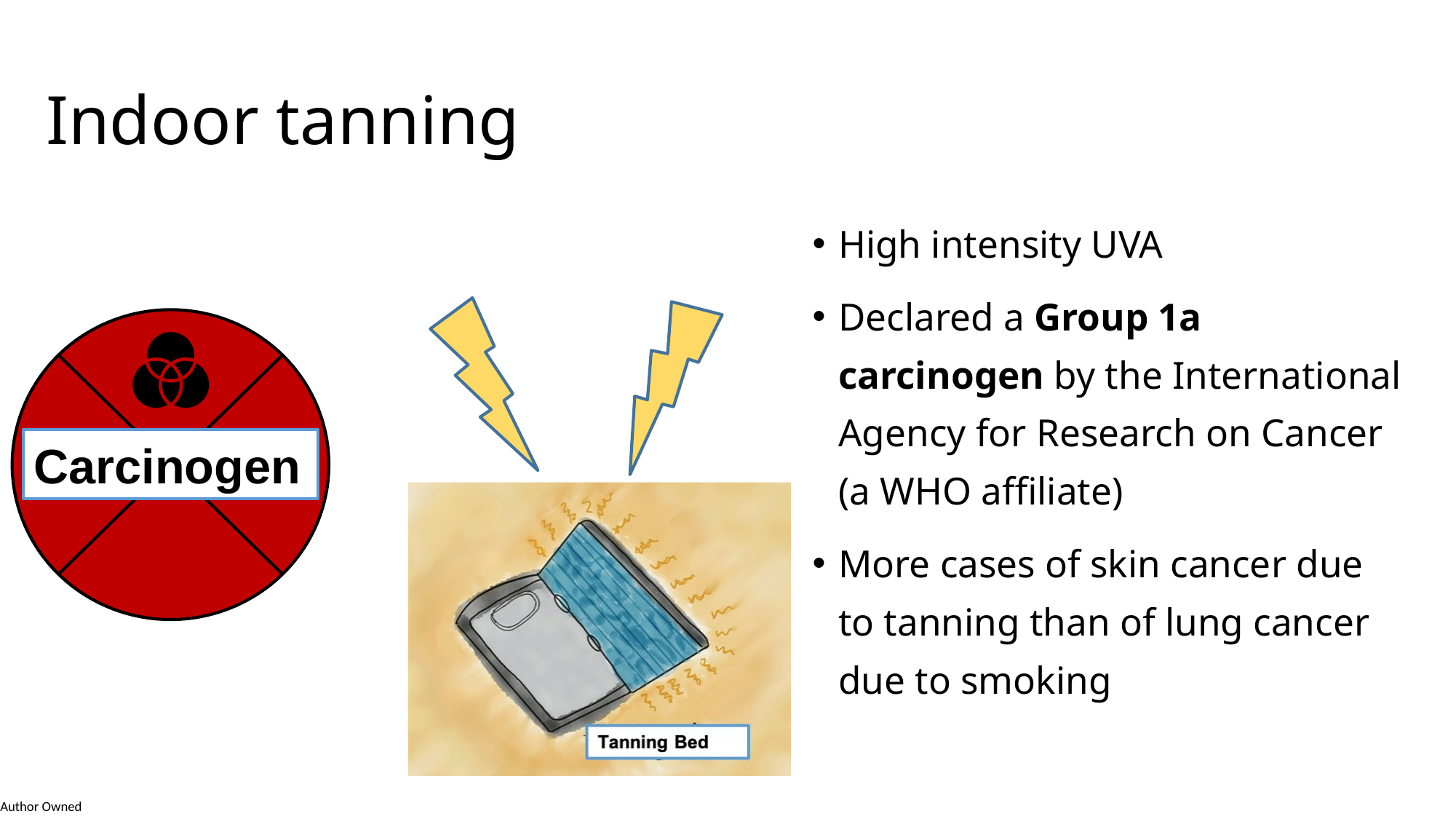

# Indoor tanning
High intensity UVA
Declared a Group 1a carcinogen by the International Agency for Research on Cancer (a WHO affiliate)
More cases of skin cancer due to tanning than of lung cancer due to smoking
Carcinogen
Author Owned

## Slide 36
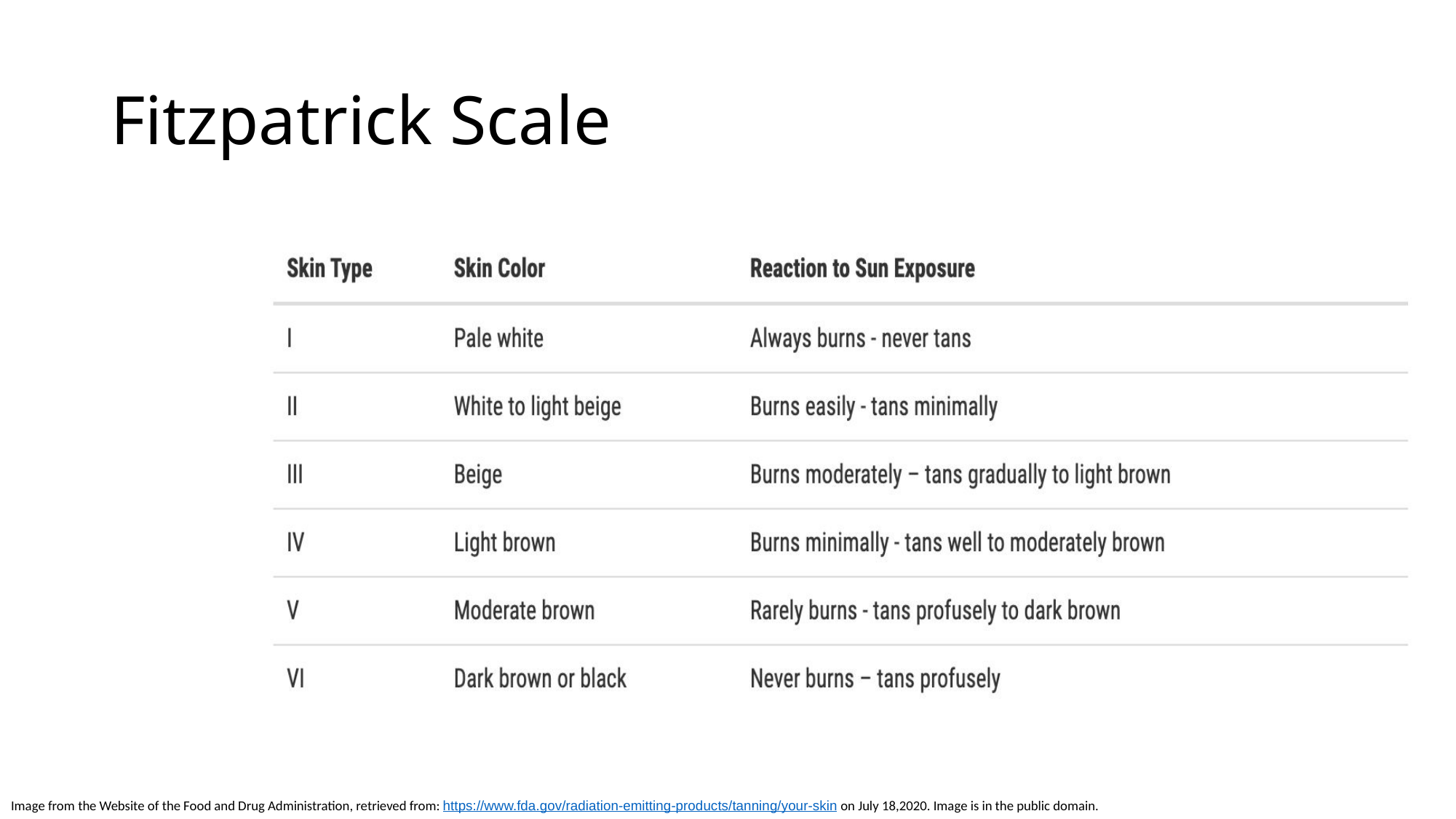

# Fitzpatrick Scale
Image from the Website of the Food and Drug Administration, retrieved from: https://www.fda.gov/radiation-emitting-products/tanning/your-skin on July 18,2020. Image is in the public domain.

## Slide 37
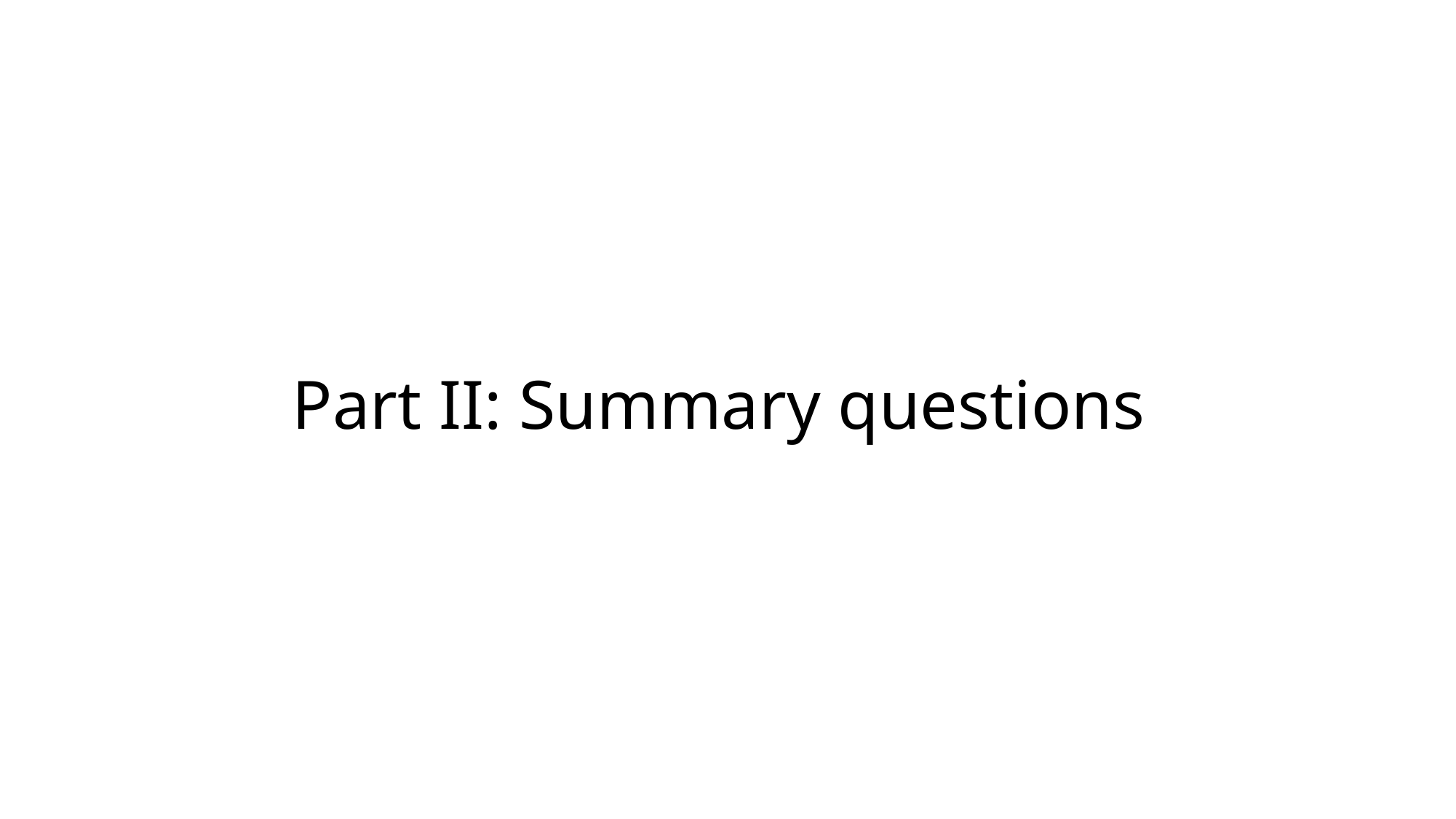

# Part II: Summary questions

## Slide 38
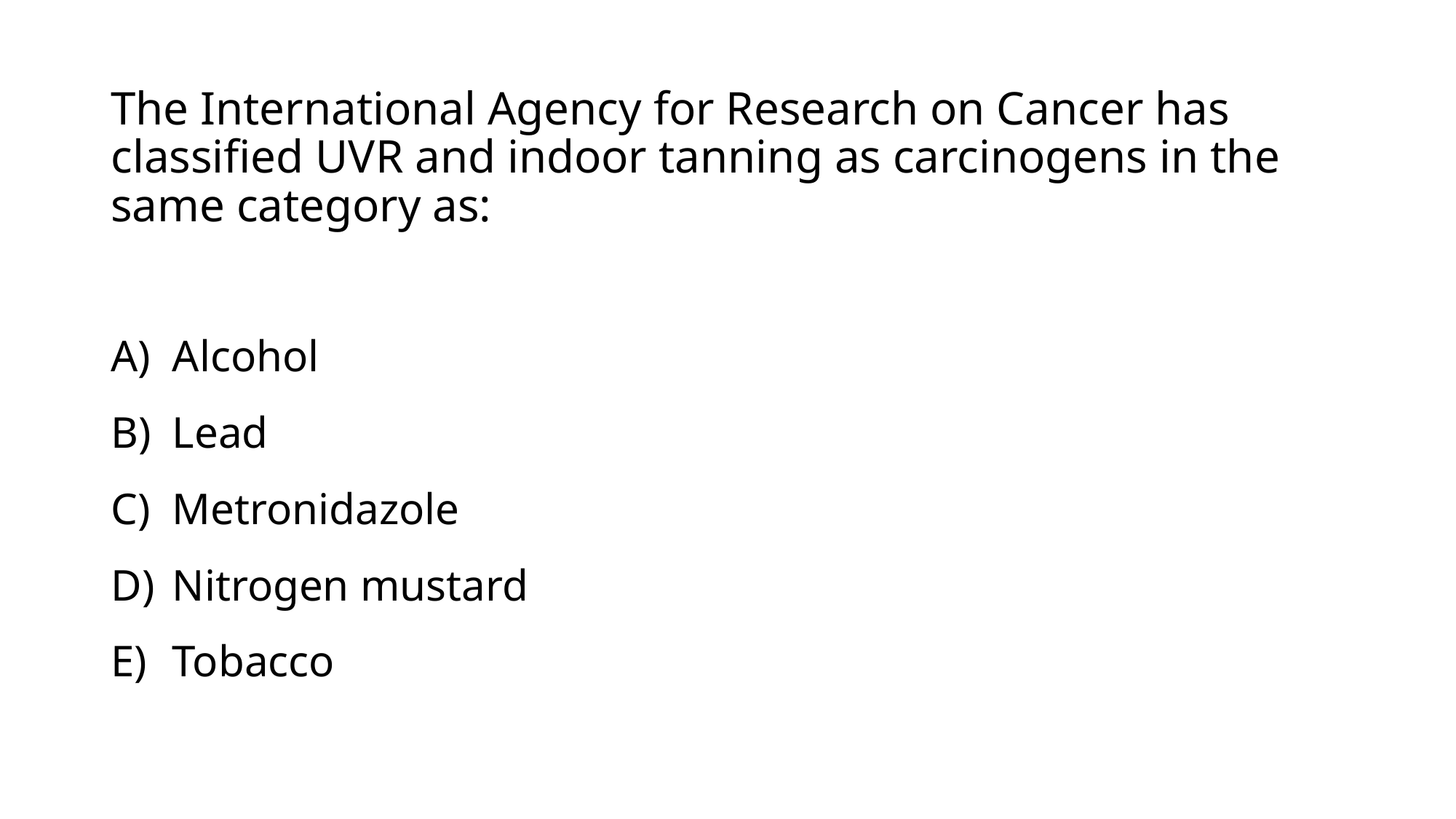

# The International Agency for Research on Cancer has classified UVR and indoor tanning as carcinogens in the same category as:
Alcohol
Lead
Metronidazole
Nitrogen mustard
Tobacco

## Slide 39
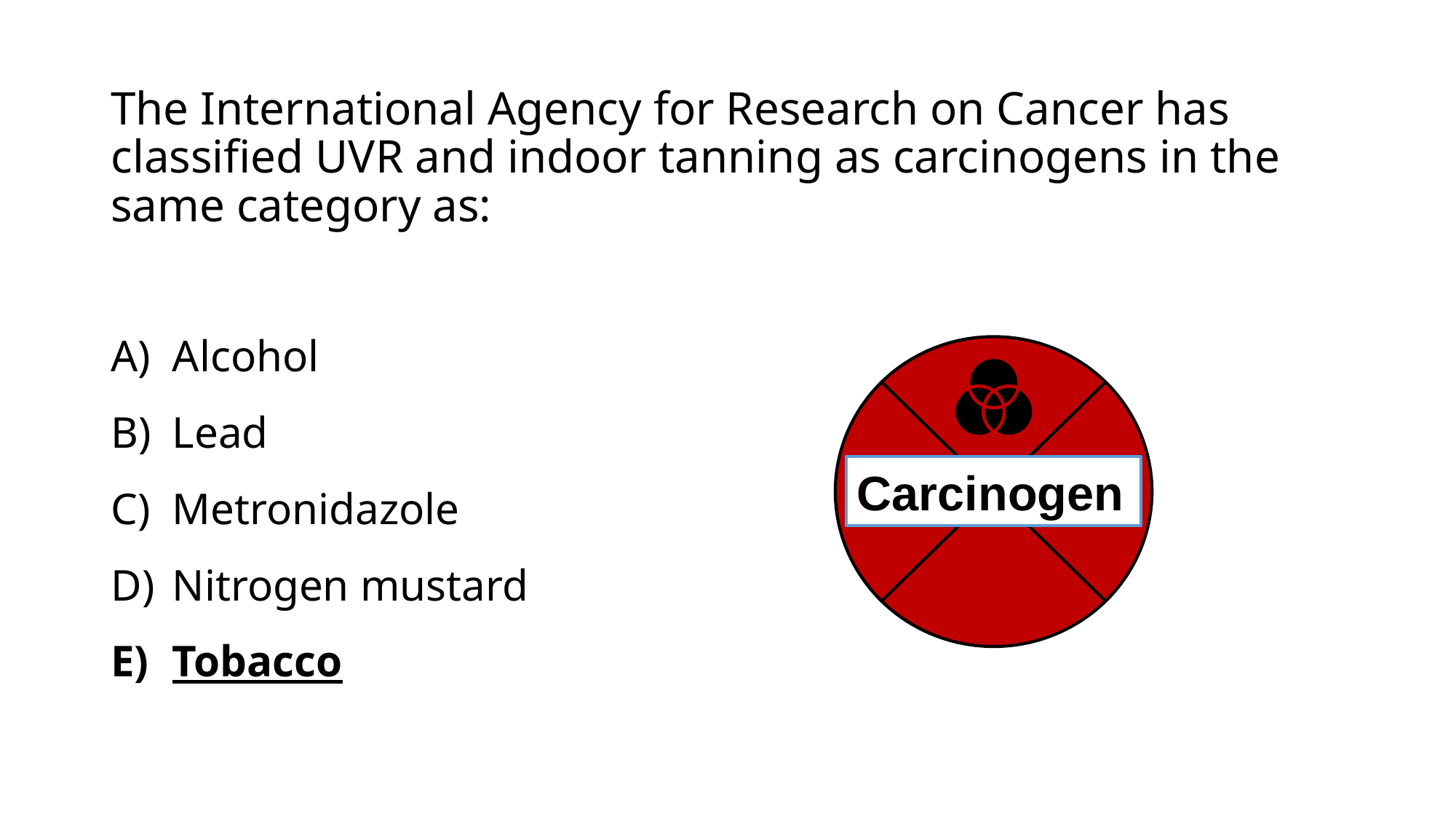

# The International Agency for Research on Cancer has classified UVR and indoor tanning as carcinogens in the same category as:
Alcohol
Lead
Metronidazole
Nitrogen mustard
Tobacco
Carcinogen

## Slide 40
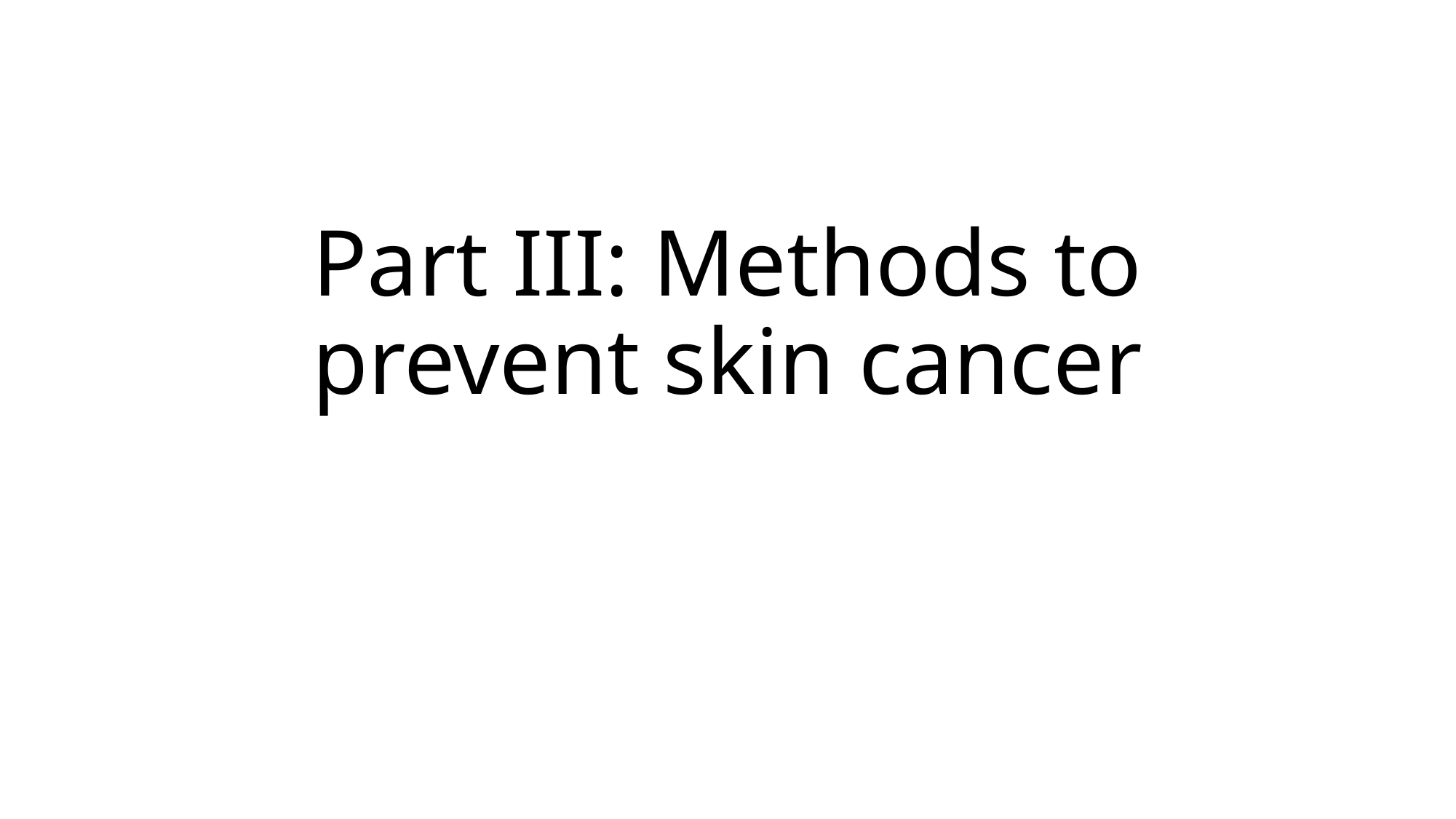

# Part III: Methods to prevent skin cancer

## Slide 41
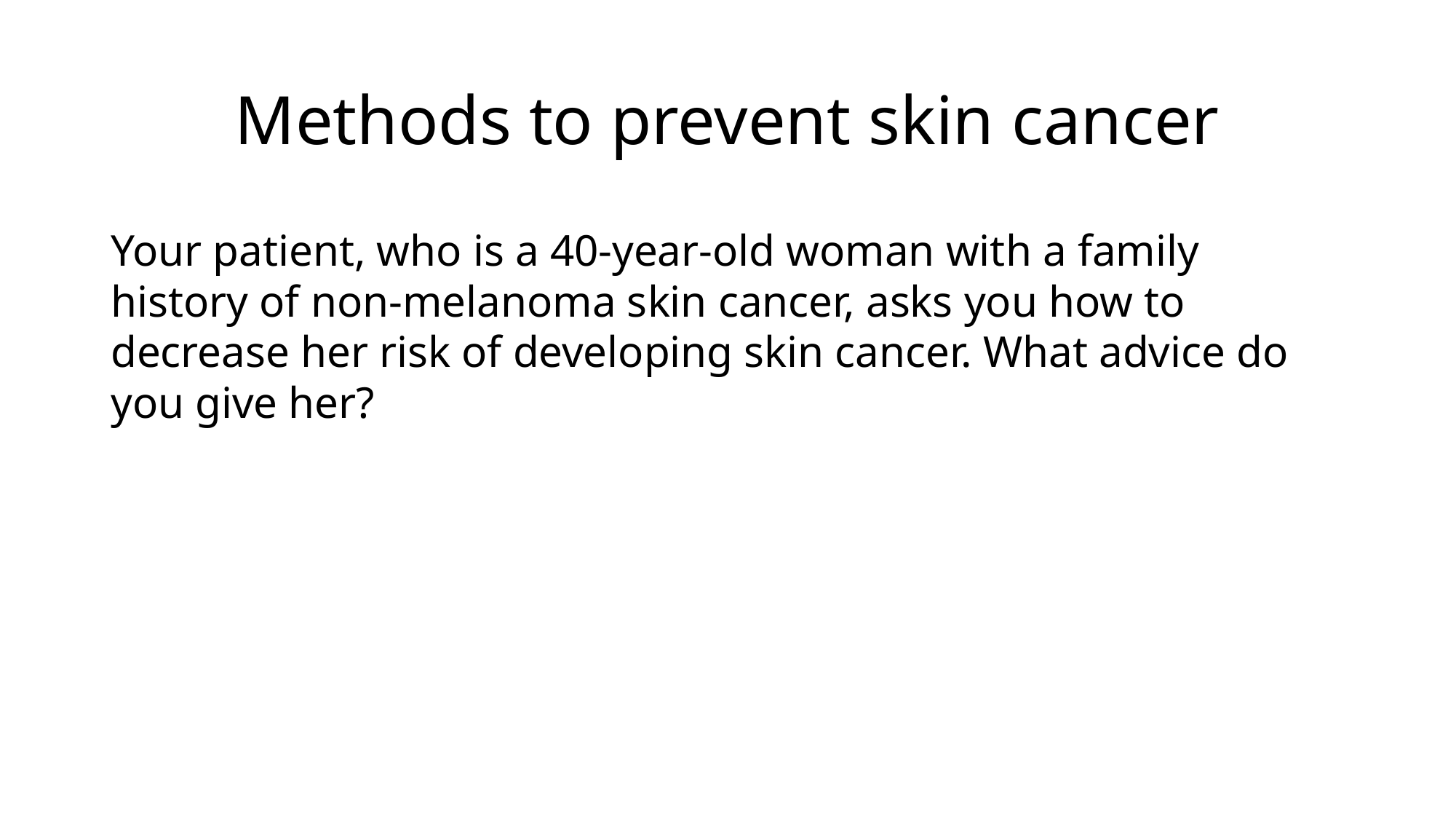

# Methods to prevent skin cancer
Your patient, who is a 40-year-old woman with a family history of non-melanoma skin cancer, asks you how to decrease her risk of developing skin cancer. What advice do you give her?

## Slide 42
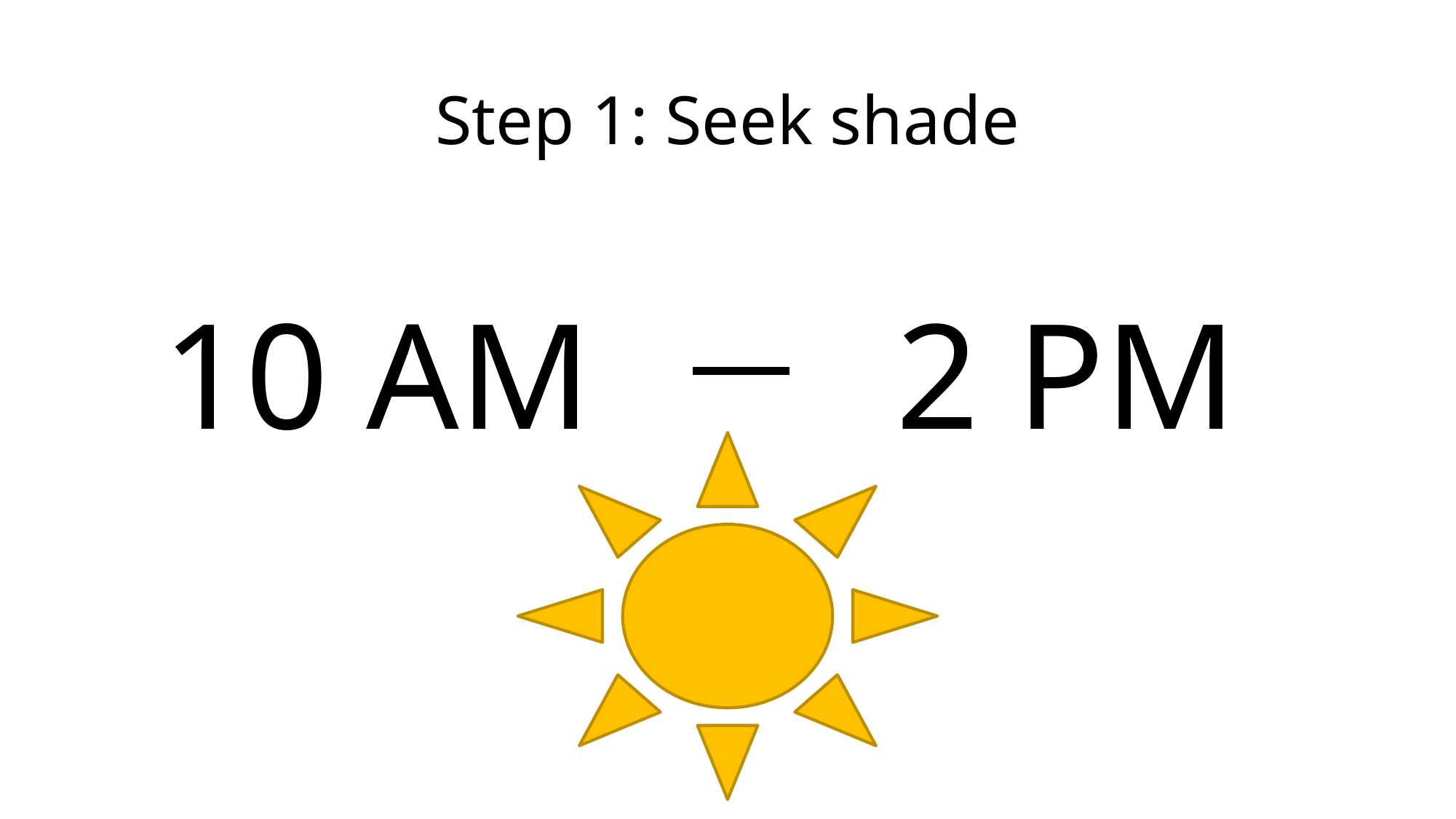

# Step 1: Seek shade
10 AM
2 PM

## Slide 43
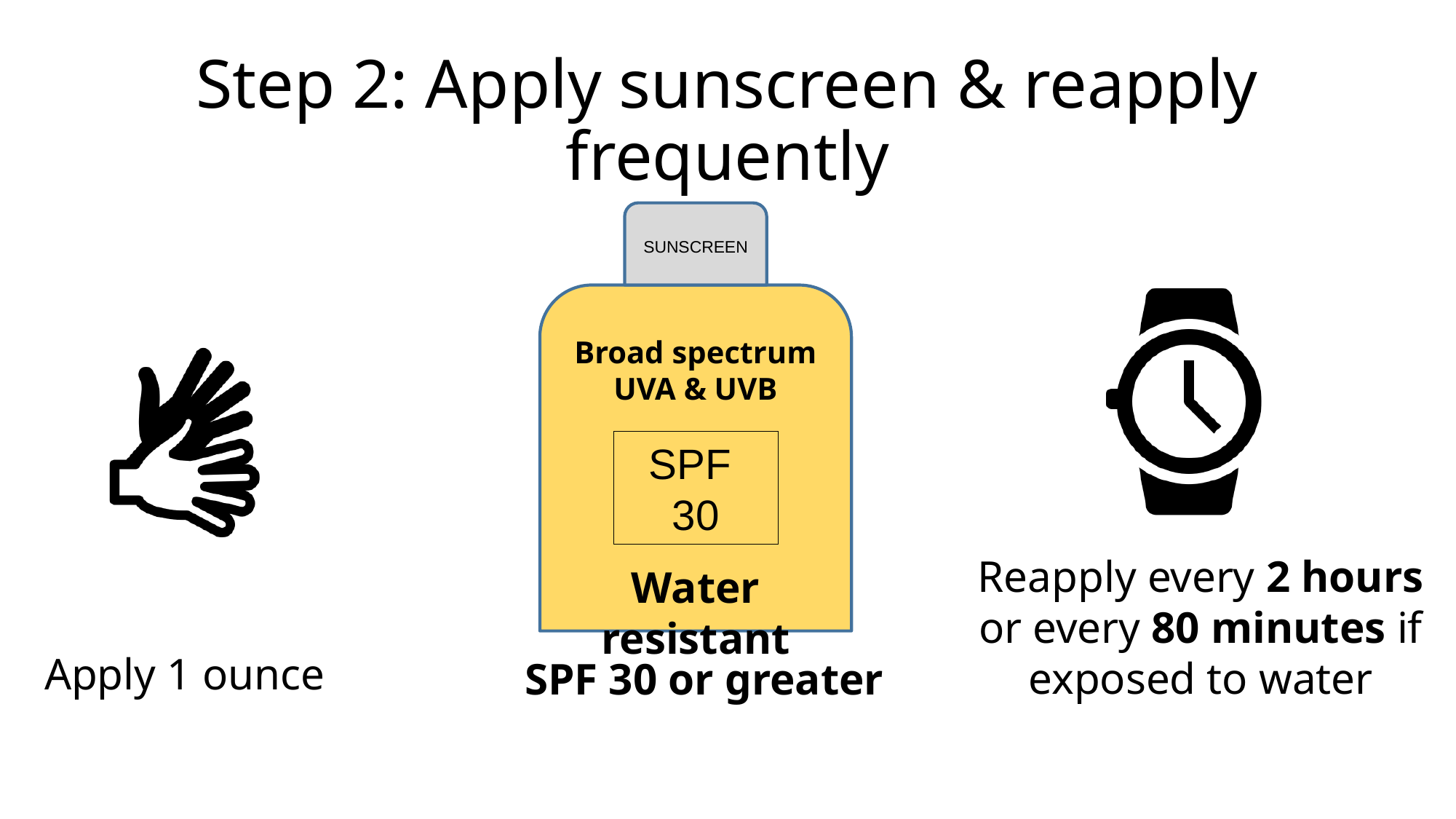

# Step 2: Apply sunscreen & reapply frequently
SUNSCREEN
Broad spectrum
UVA & UVB
SPF
30
Reapply every 2 hours
or every 80 minutes if exposed to water
Water resistant
Apply 1 ounce
SPF 30 or greater

## Slide 44
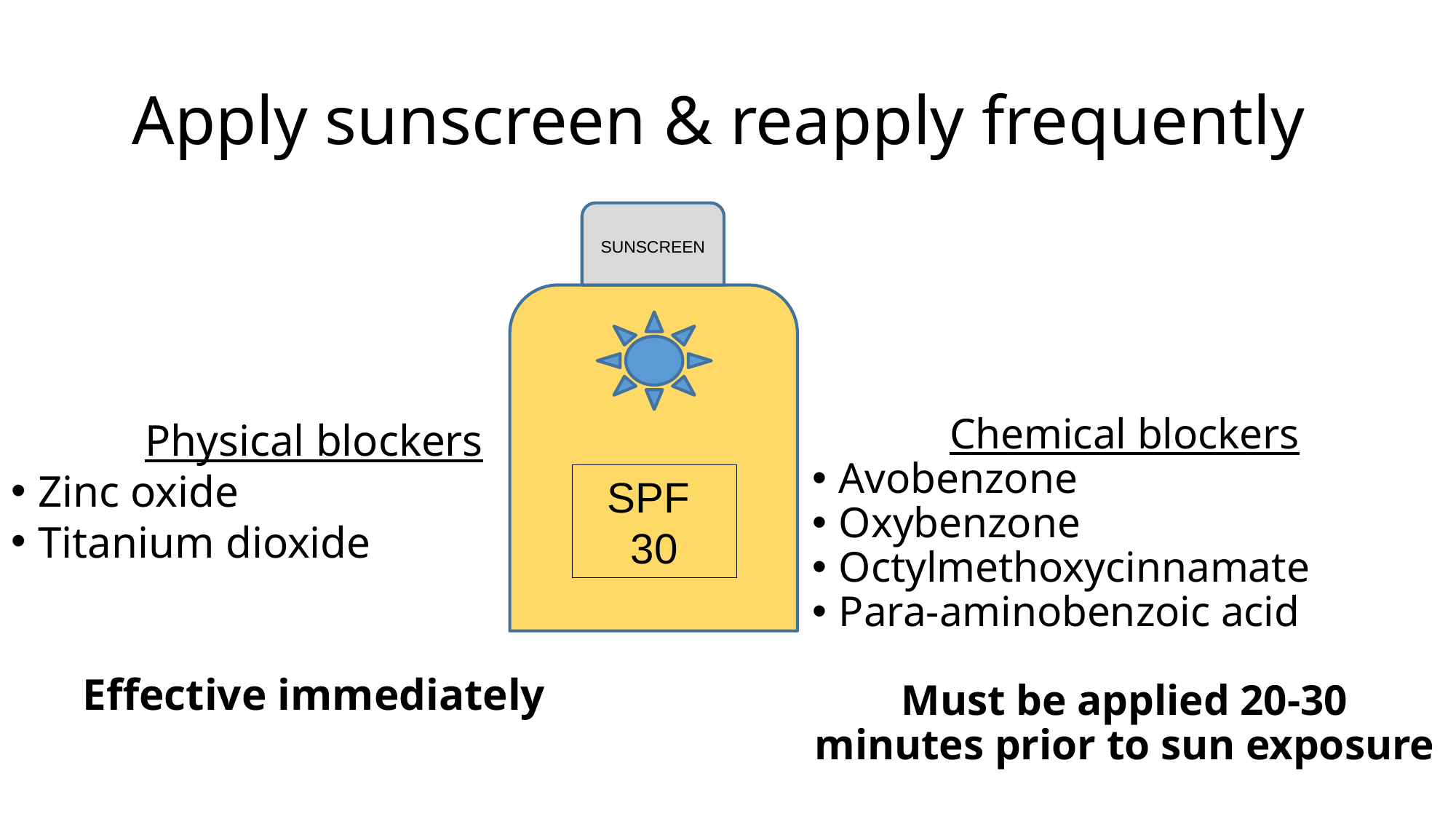

# Apply sunscreen & reapply frequently
SUNSCREEN
Physical blockers
Zinc oxide
Titanium dioxide
Effective immediately
Chemical blockers
Avobenzone
Oxybenzone
Octylmethoxycinnamate
Para-aminobenzoic acid
Must be applied 20-30 minutes prior to sun exposure
SPF
30

## Slide 45
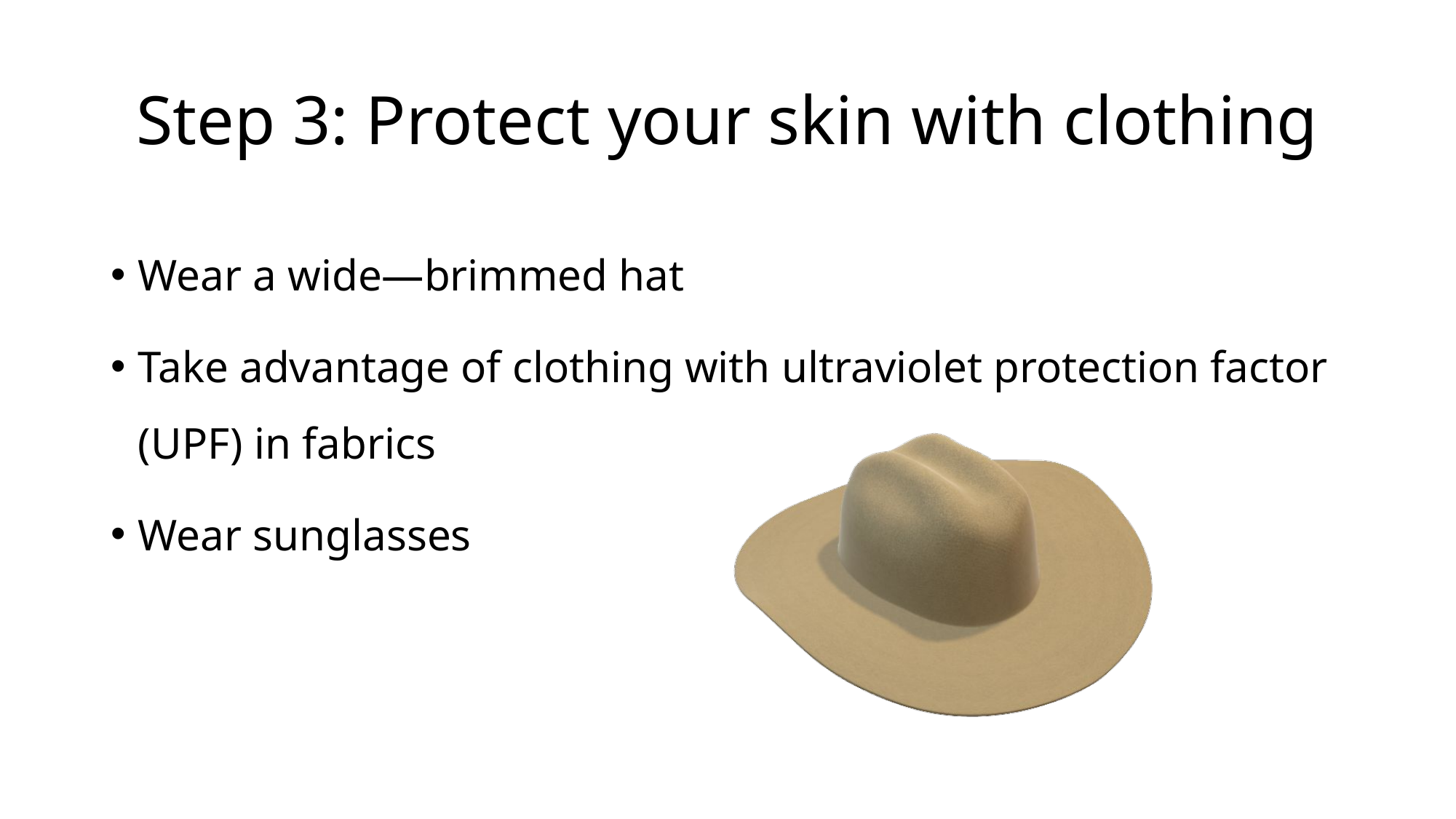

# Step 3: Protect your skin with clothing
Wear a wide—brimmed hat
Take advantage of clothing with ultraviolet protection factor (UPF) in fabrics
Wear sunglasses

## Slide 46
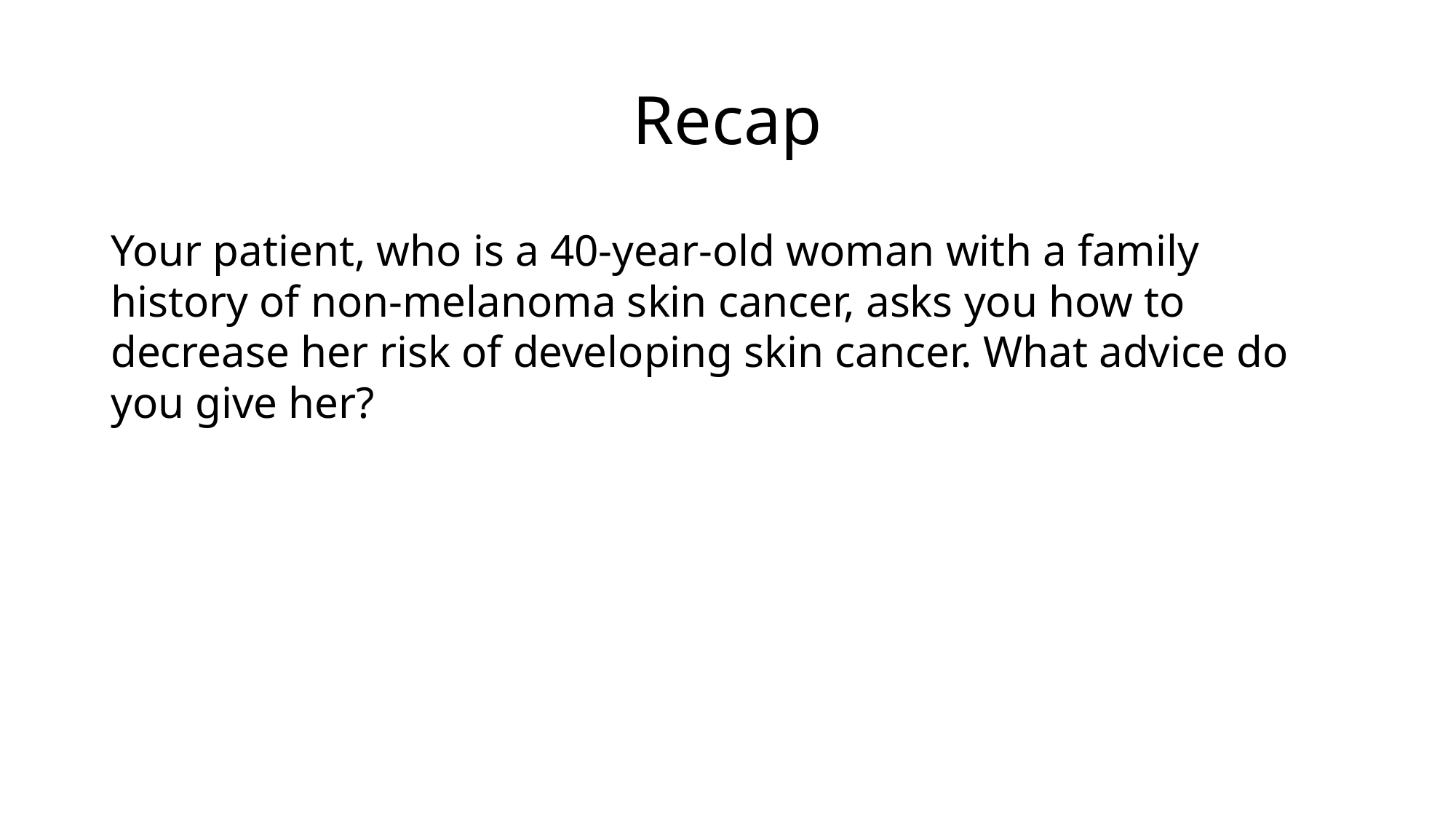

# Recap
Your patient, who is a 40-year-old woman with a family history of non-melanoma skin cancer, asks you how to decrease her risk of developing skin cancer. What advice do you give her?

## Slide 47
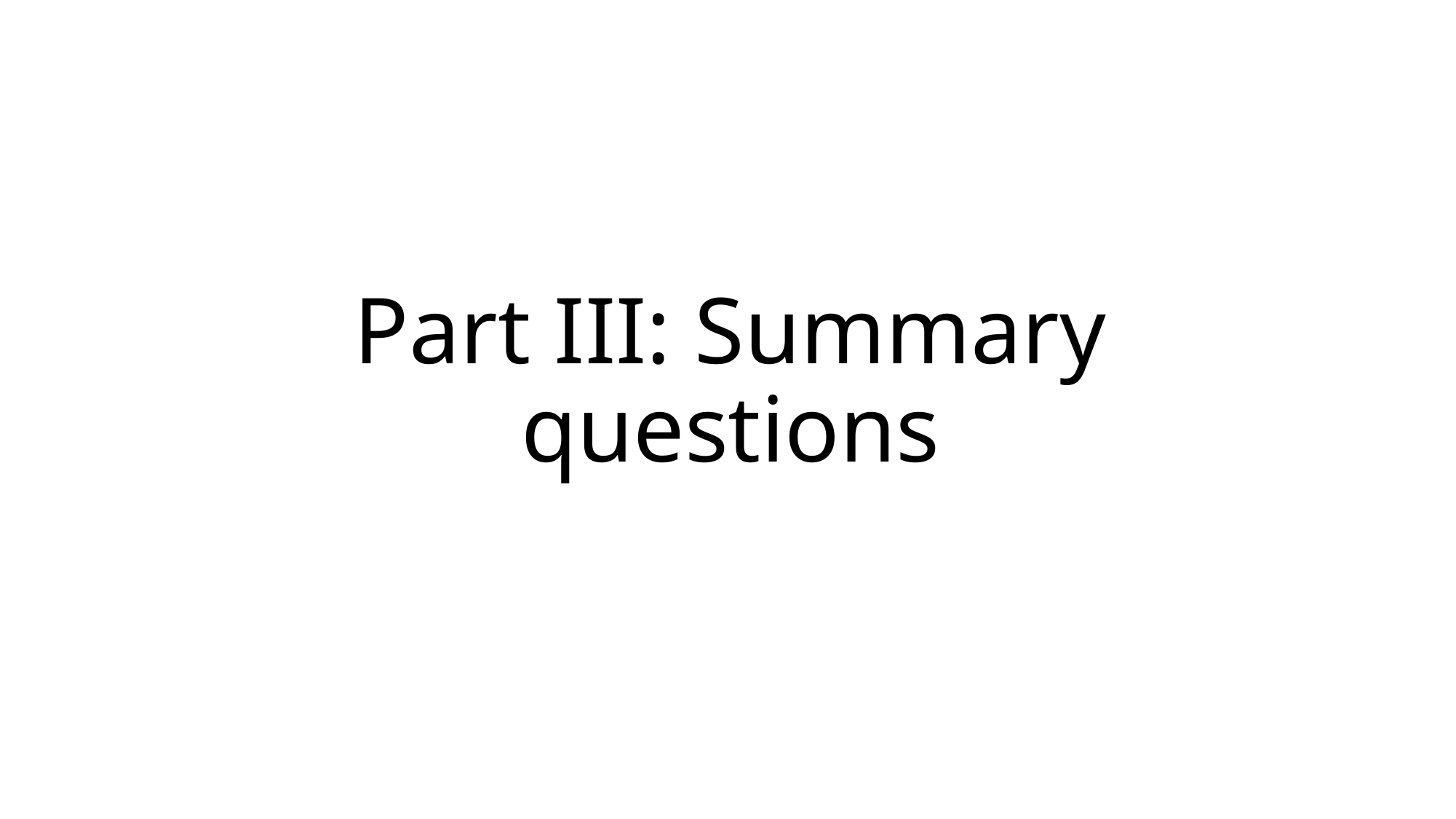

# Part III: Summary questions

## Slide 48
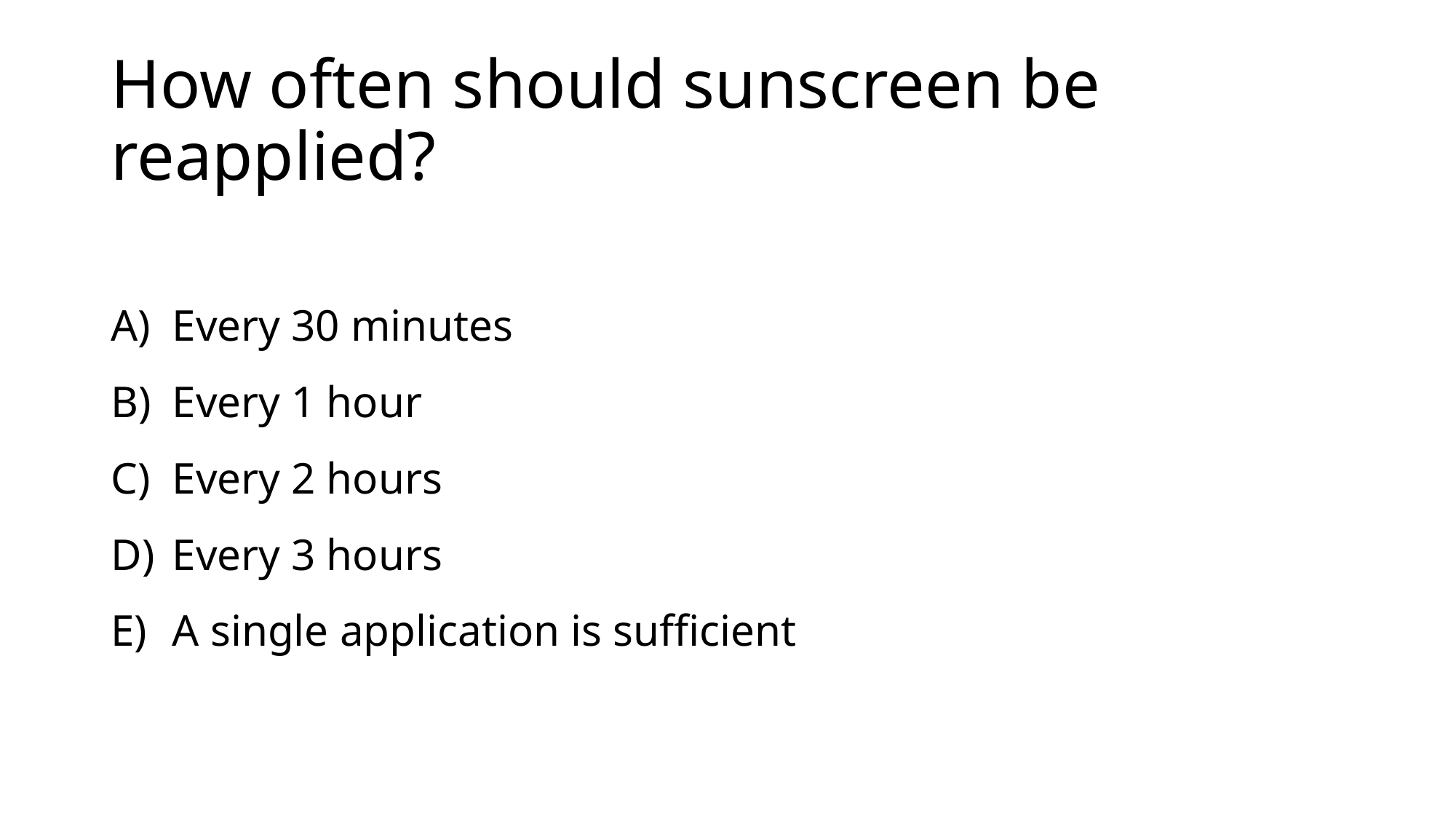

# How often should sunscreen be reapplied?
Every 30 minutes
Every 1 hour
Every 2 hours
Every 3 hours
A single application is sufficient

## Slide 49
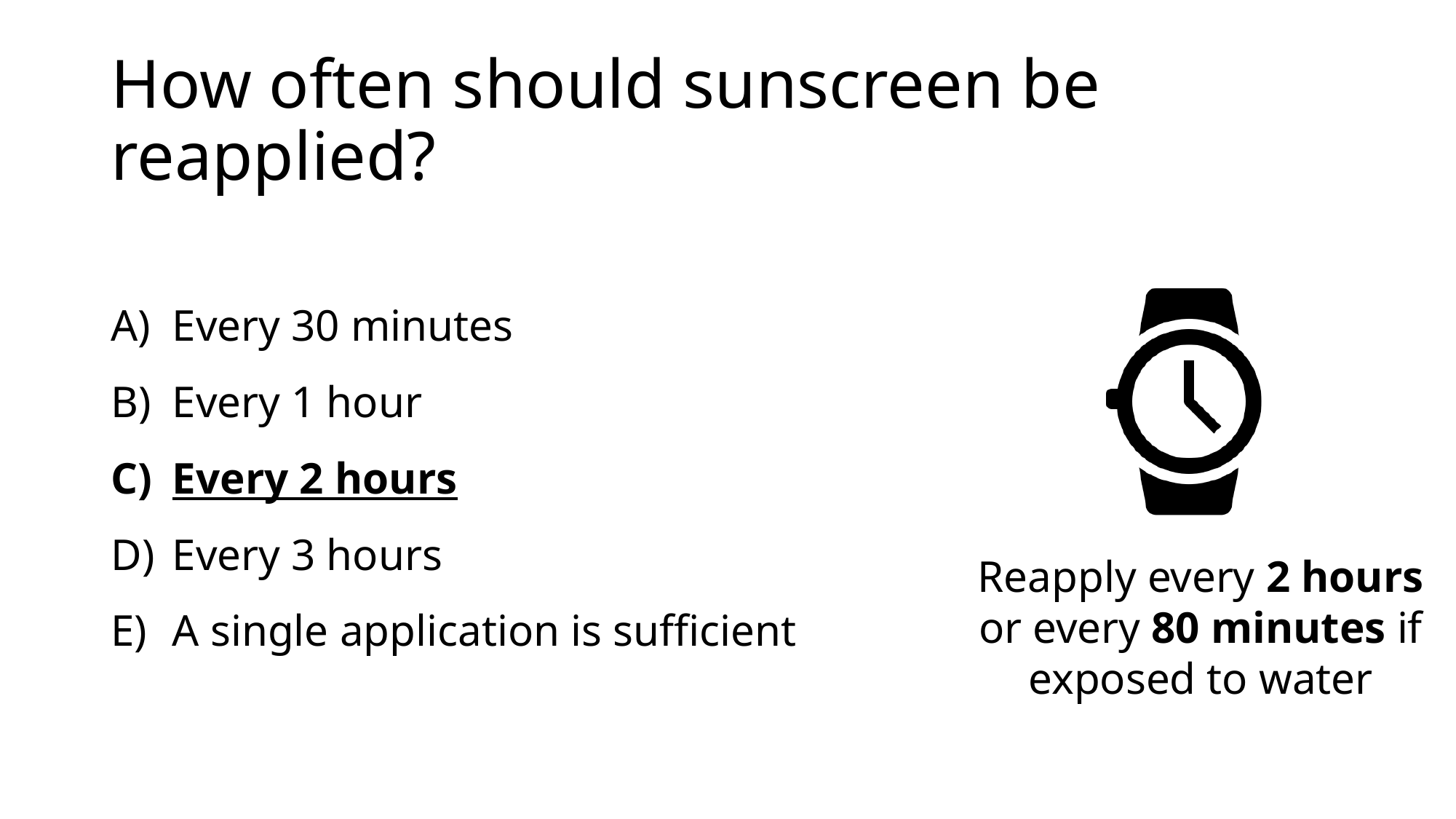

# How often should sunscreen be reapplied?
Every 30 minutes
Every 1 hour
Every 2 hours
Every 3 hours
A single application is sufficient
Reapply every 2 hours
or every 80 minutes if exposed to water

## Slide 50
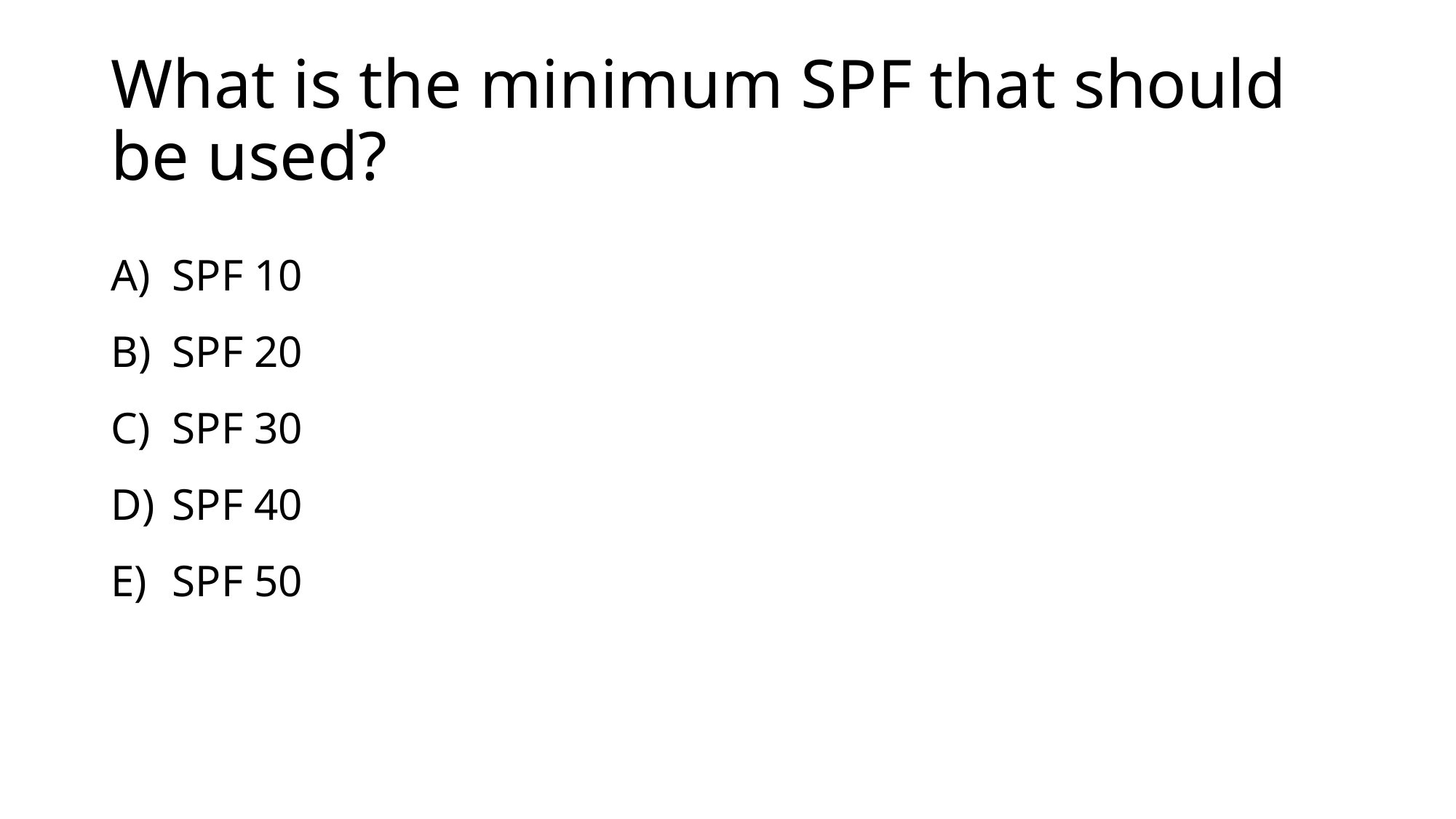

# What is the minimum SPF that should be used?
SPF 10
SPF 20
SPF 30
SPF 40
SPF 50

## Slide 51
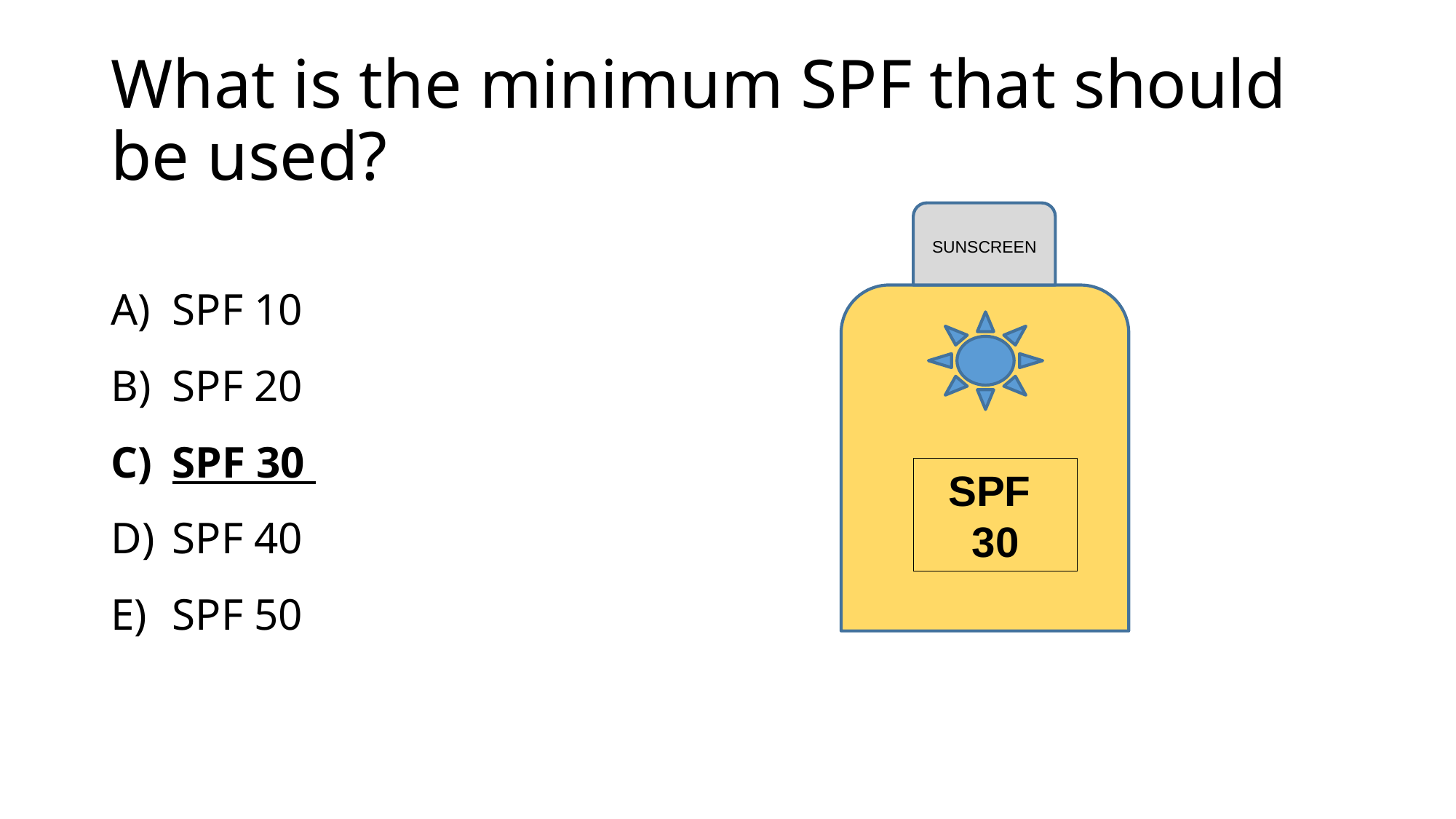

# What is the minimum SPF that should be used?
SUNSCREEN
SPF 10
SPF 20
SPF 30
SPF 40
SPF 50
SPF
30

## Slide 52
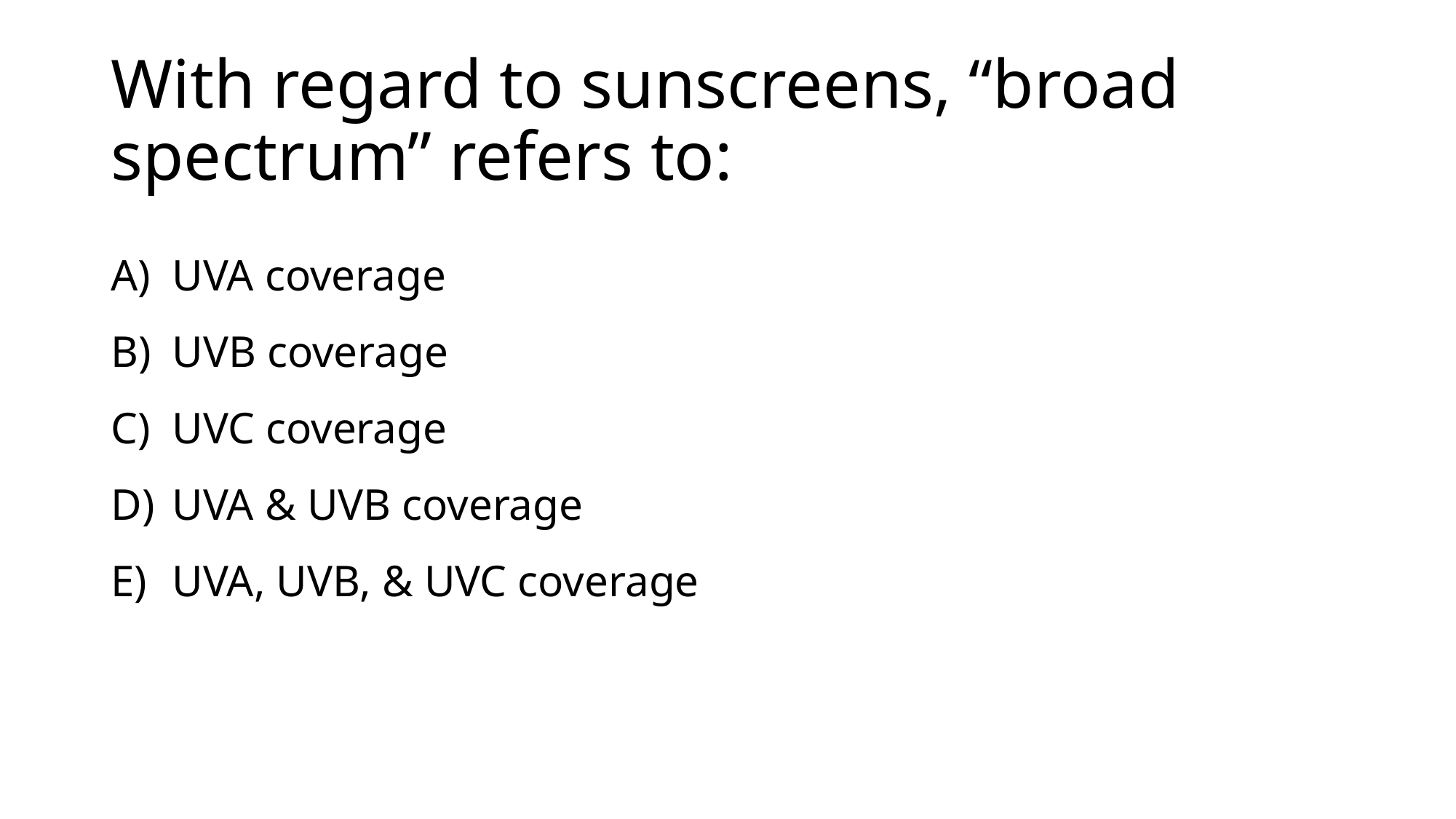

# With regard to sunscreens, “broad spectrum” refers to:
UVA coverage
UVB coverage
UVC coverage
UVA & UVB coverage
UVA, UVB, & UVC coverage

## Slide 53
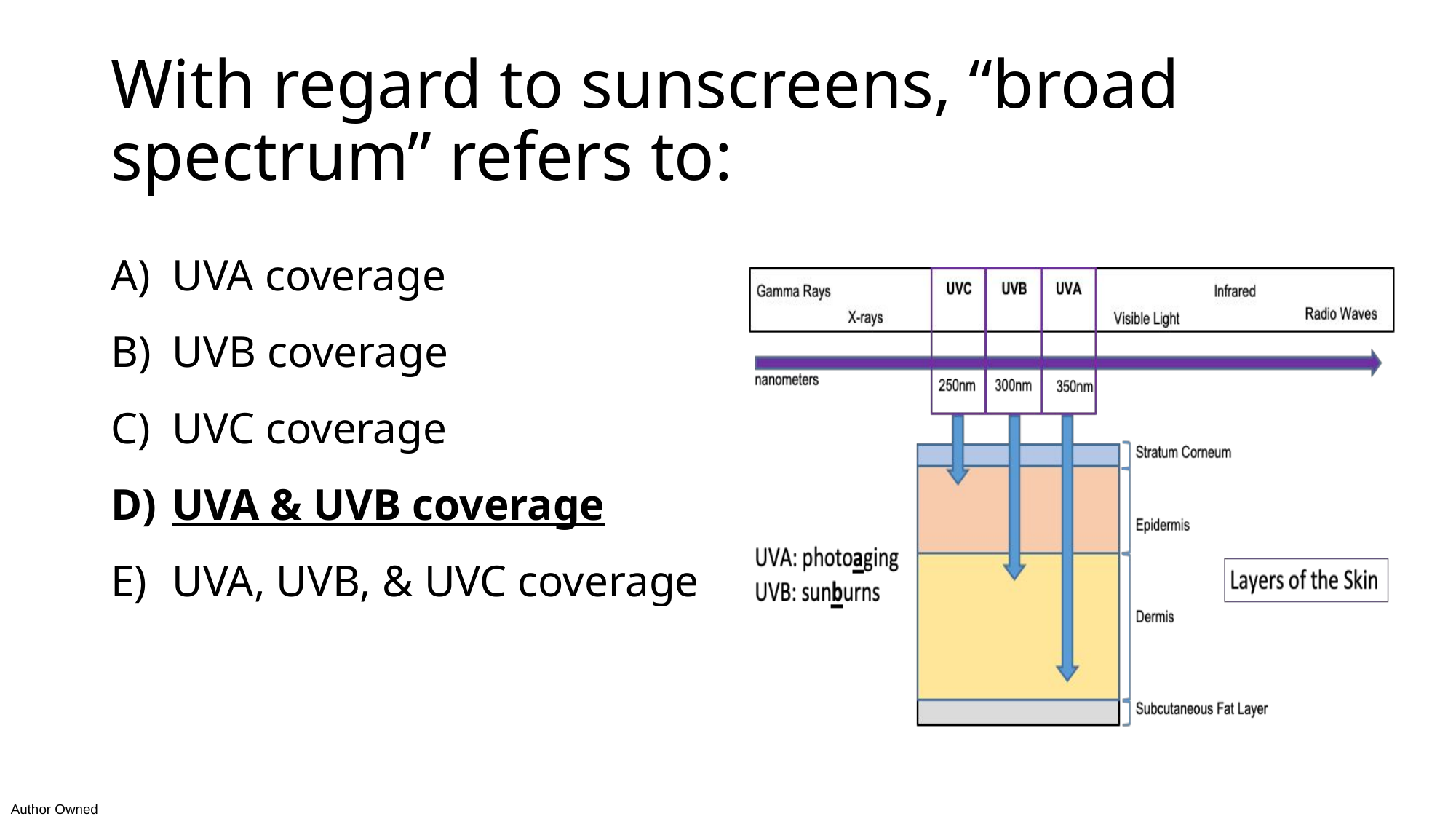

# With regard to sunscreens, “broad spectrum” refers to:
UVA coverage
UVB coverage
UVC coverage
UVA & UVB coverage
UVA, UVB, & UVC coverage
Author Owned

## Slide 54
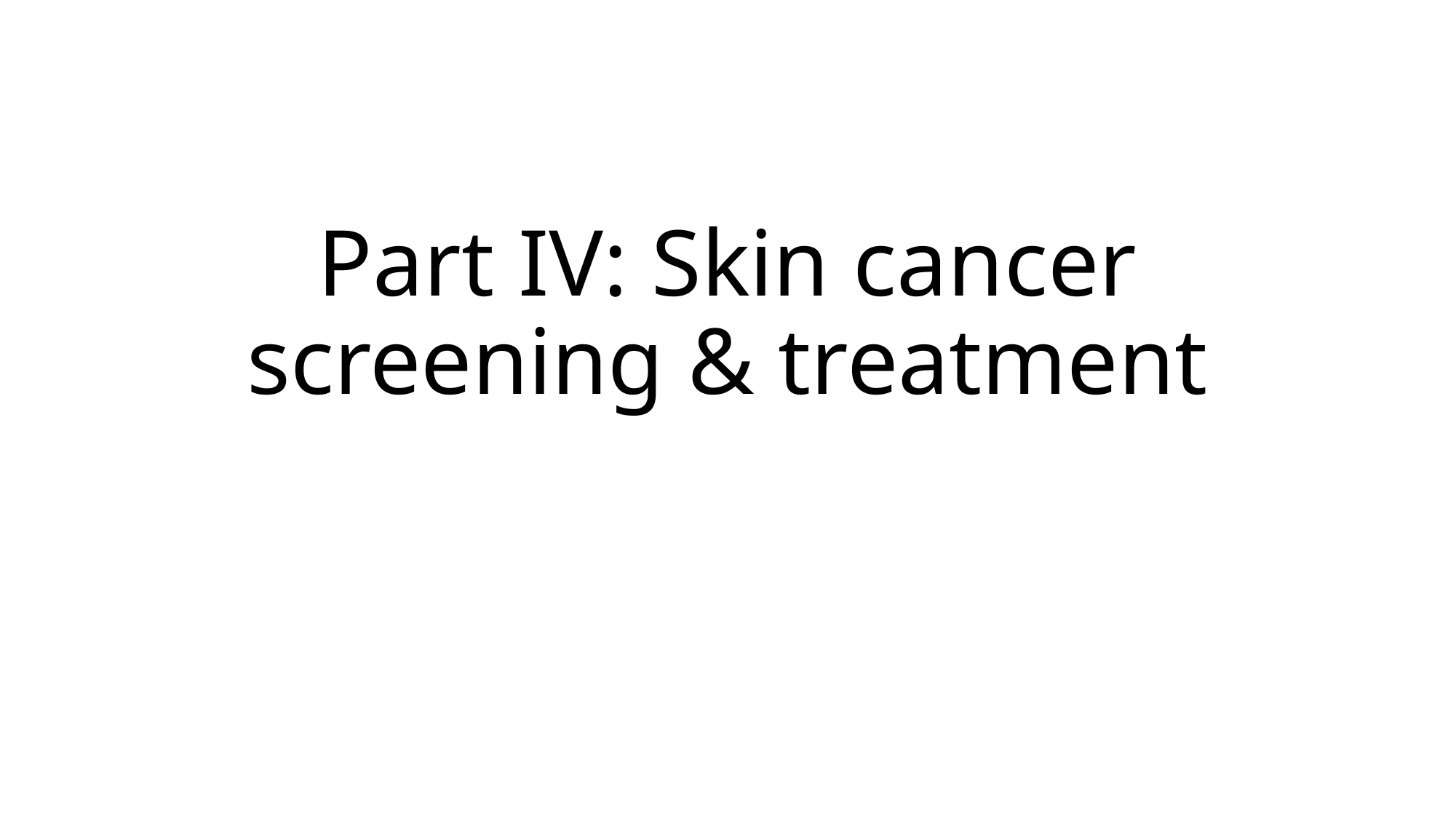

# Part IV: Skin cancer screening & treatment

## Slide 55
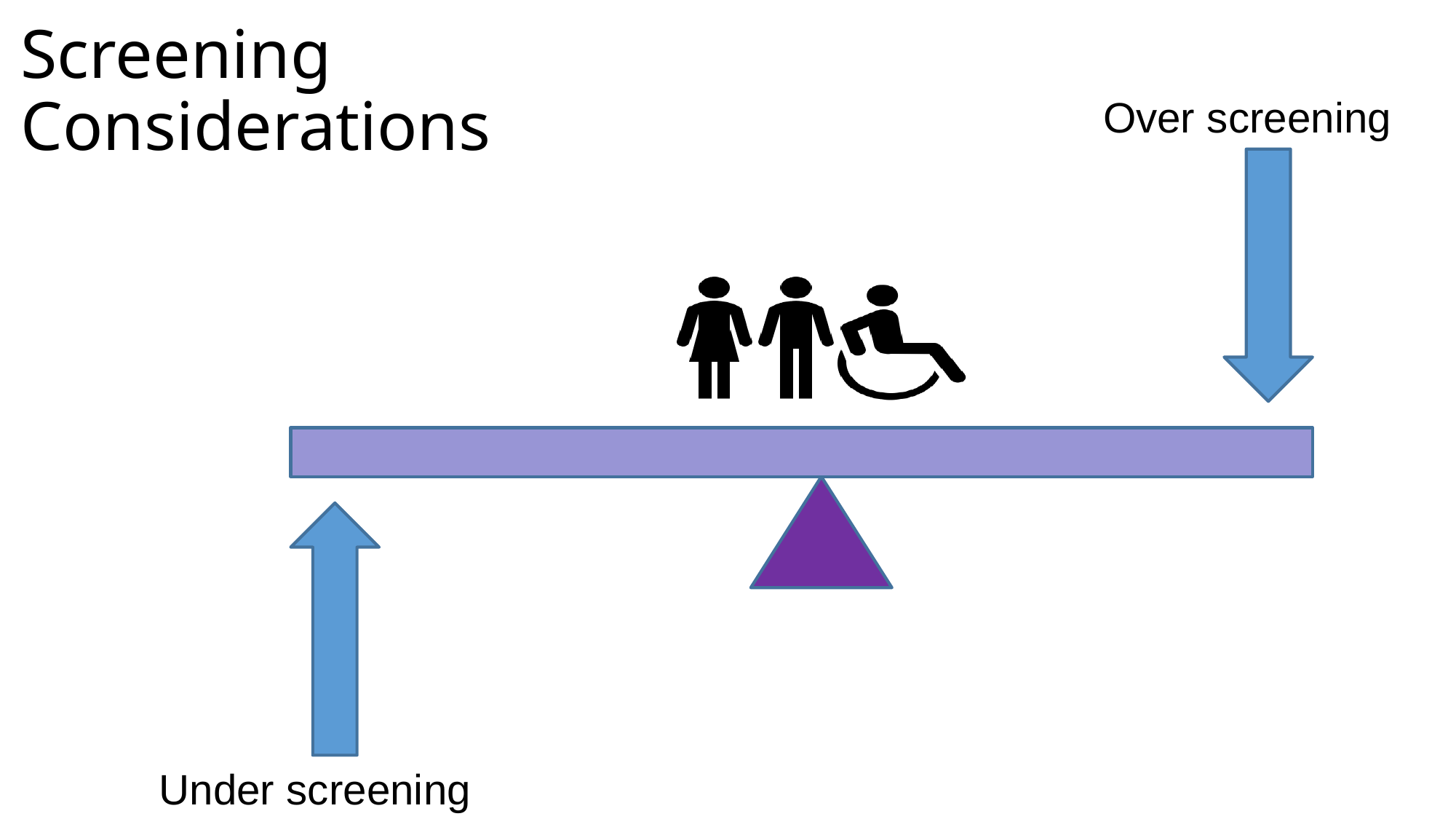

# Screening Considerations
Over screening
Under screening

## Slide 56
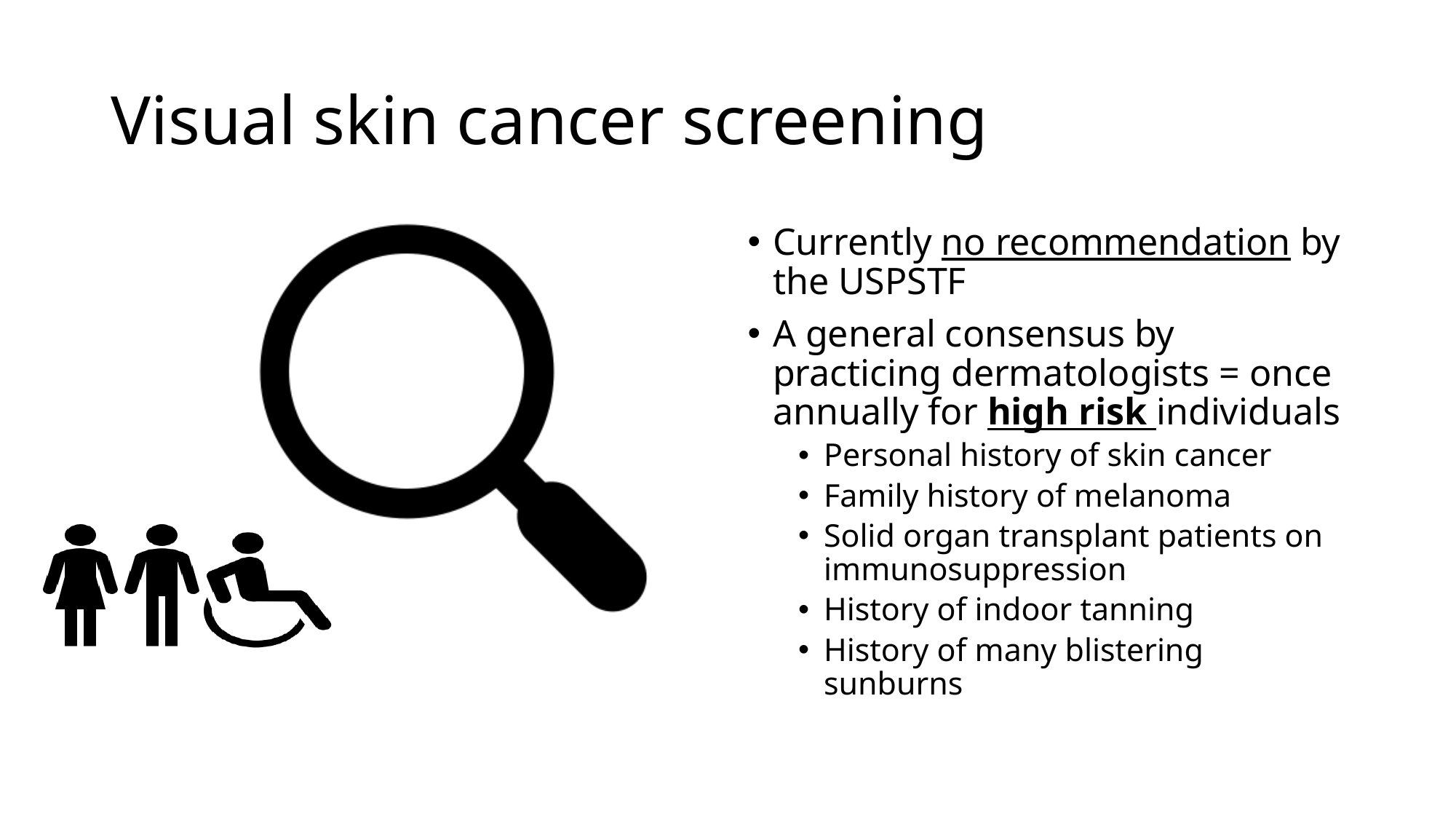

# Visual skin cancer screening
Currently no recommendation by the USPSTF
A general consensus by practicing dermatologists = once annually for high risk individuals
Personal history of skin cancer
Family history of melanoma
Solid organ transplant patients on immunosuppression
History of indoor tanning
History of many blistering sunburns

## Slide 57
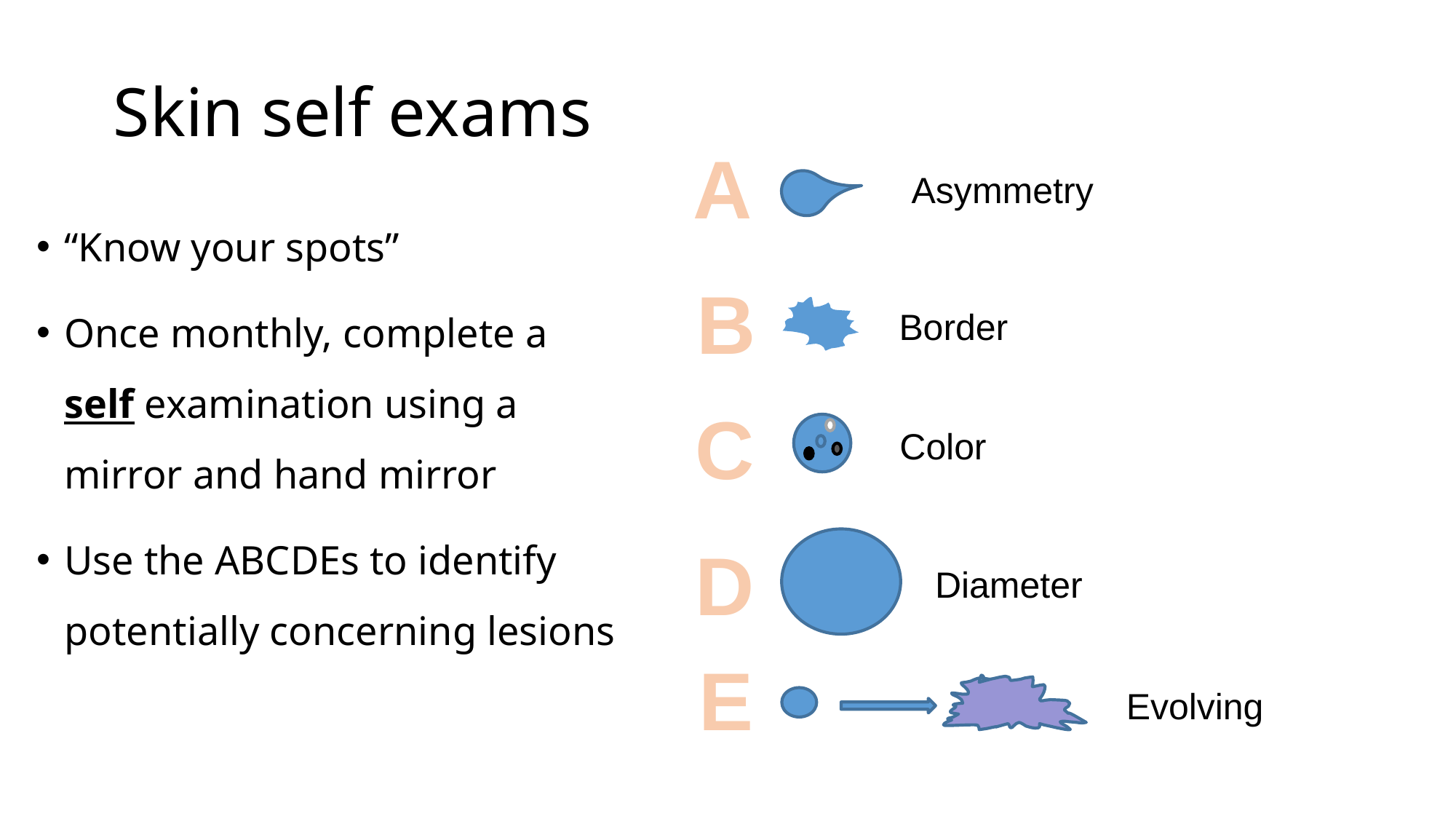

# Skin self exams
A
Asymmetry
“Know your spots”
Once monthly, complete a self examination using a mirror and hand mirror
Use the ABCDEs to identify potentially concerning lesions
B
Border
C
Color
D
Diameter
E
Evolving

## Slide 58
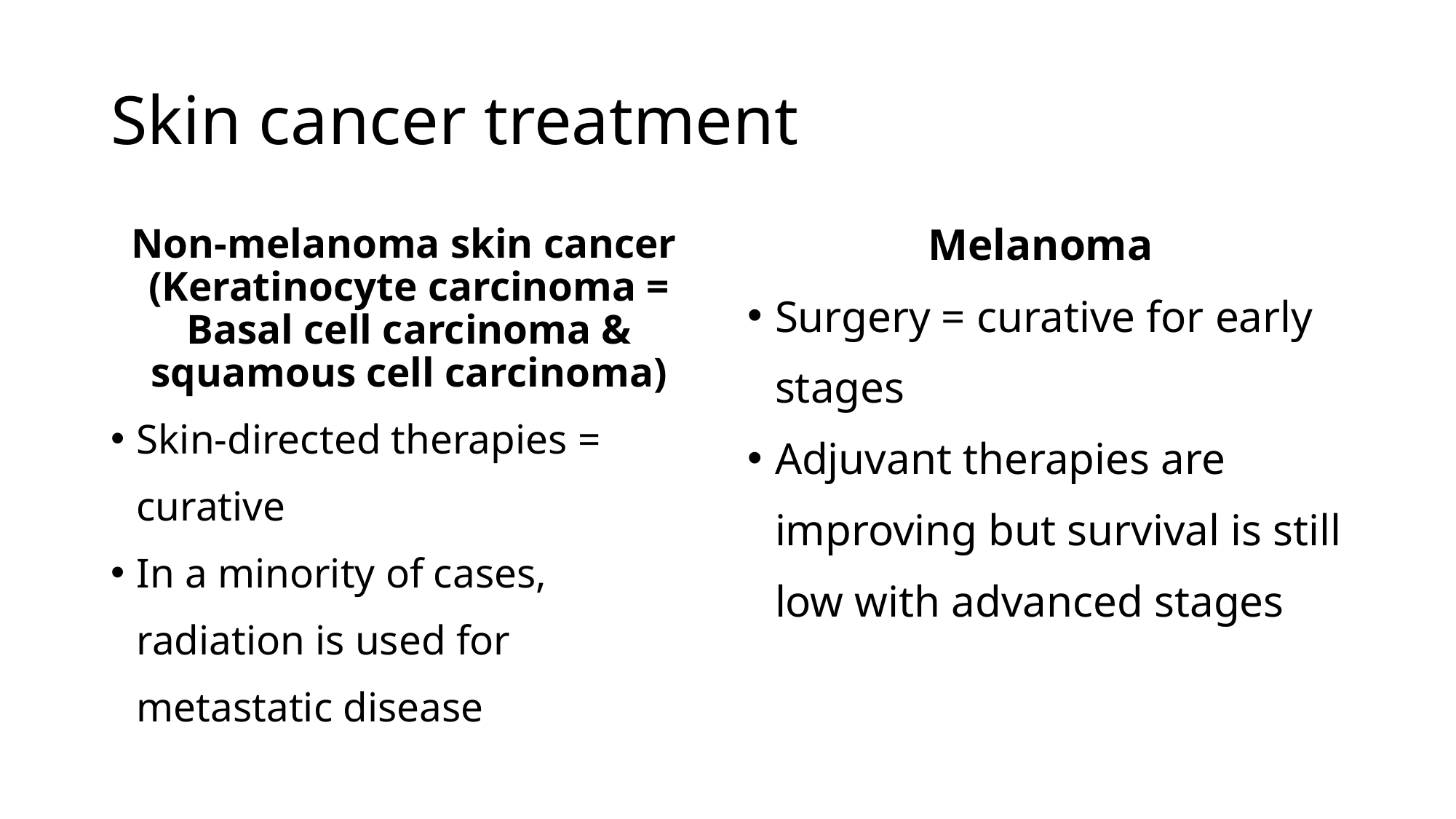

# Skin cancer treatment
Non-melanoma skin cancer
(Keratinocyte carcinoma = Basal cell carcinoma & squamous cell carcinoma)
Skin-directed therapies = curative
In a minority of cases, radiation is used for metastatic disease
Melanoma
Surgery = curative for early stages
Adjuvant therapies are improving but survival is still low with advanced stages

## Slide 59
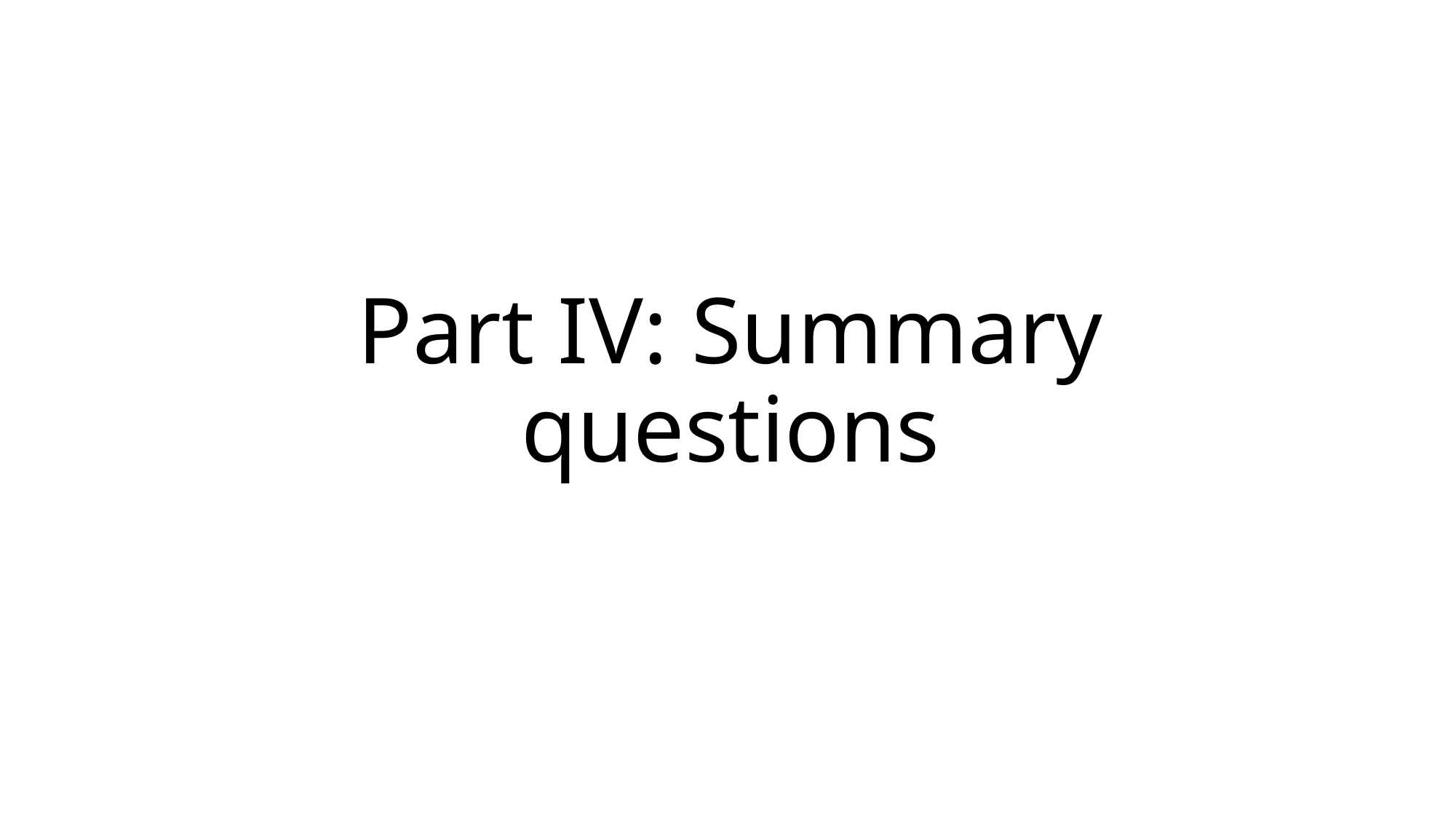

# Part IV: Summary questions

## Slide 60
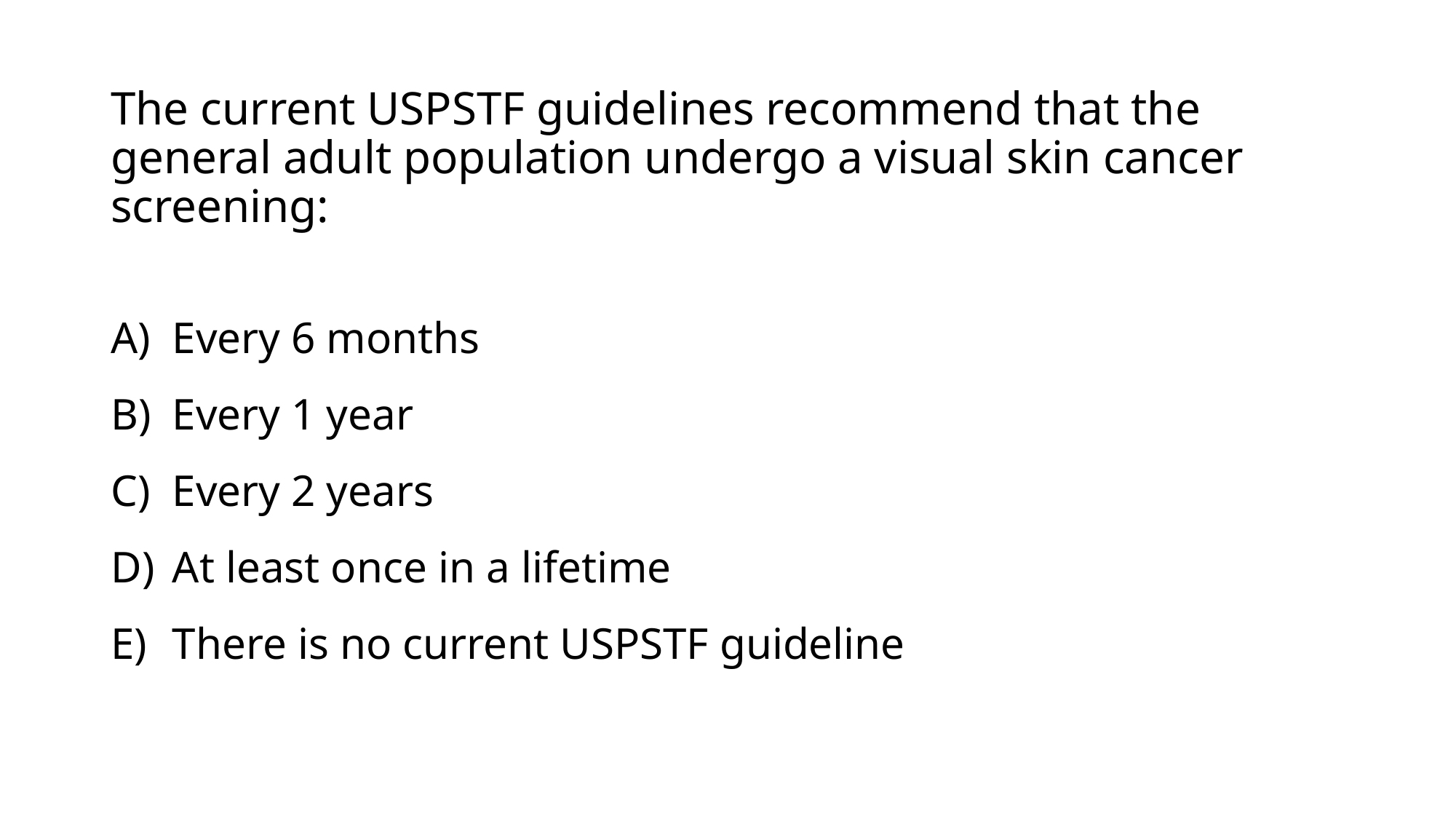

# The current USPSTF guidelines recommend that the general adult population undergo a visual skin cancer screening:
Every 6 months
Every 1 year
Every 2 years
At least once in a lifetime
There is no current USPSTF guideline

## Slide 61
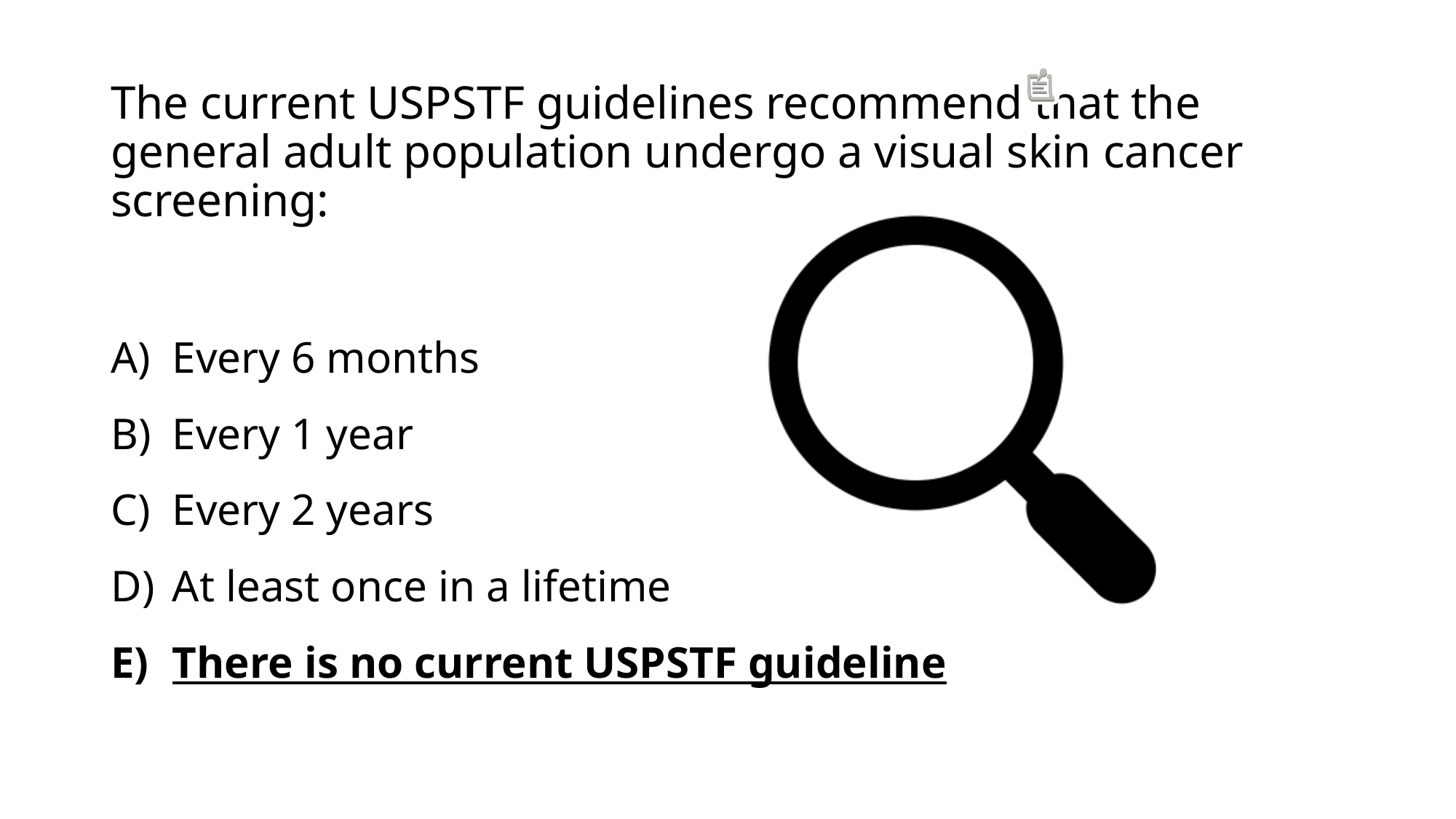

# The current USPSTF guidelines recommend that the general adult population undergo a visual skin cancer screening:
Every 6 months
Every 1 year
Every 2 years
At least once in a lifetime
There is no current USPSTF guideline

## Slide 62
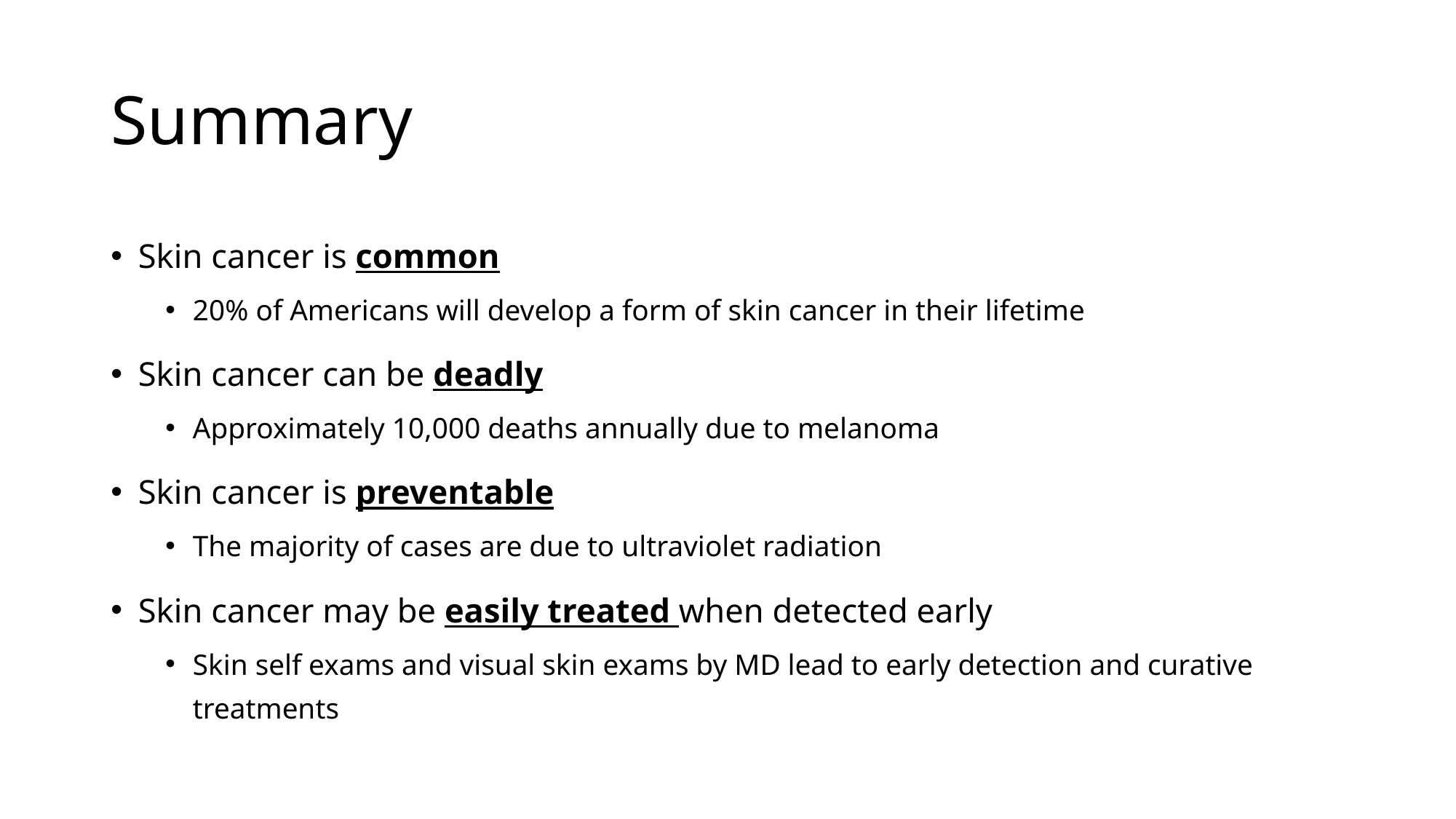

# Summary
Skin cancer is common
20% of Americans will develop a form of skin cancer in their lifetime
Skin cancer can be deadly
Approximately 10,000 deaths annually due to melanoma
Skin cancer is preventable
The majority of cases are due to ultraviolet radiation
Skin cancer may be easily treated when detected early
Skin self exams and visual skin exams by MD lead to early detection and curative treatments

## Slide 63
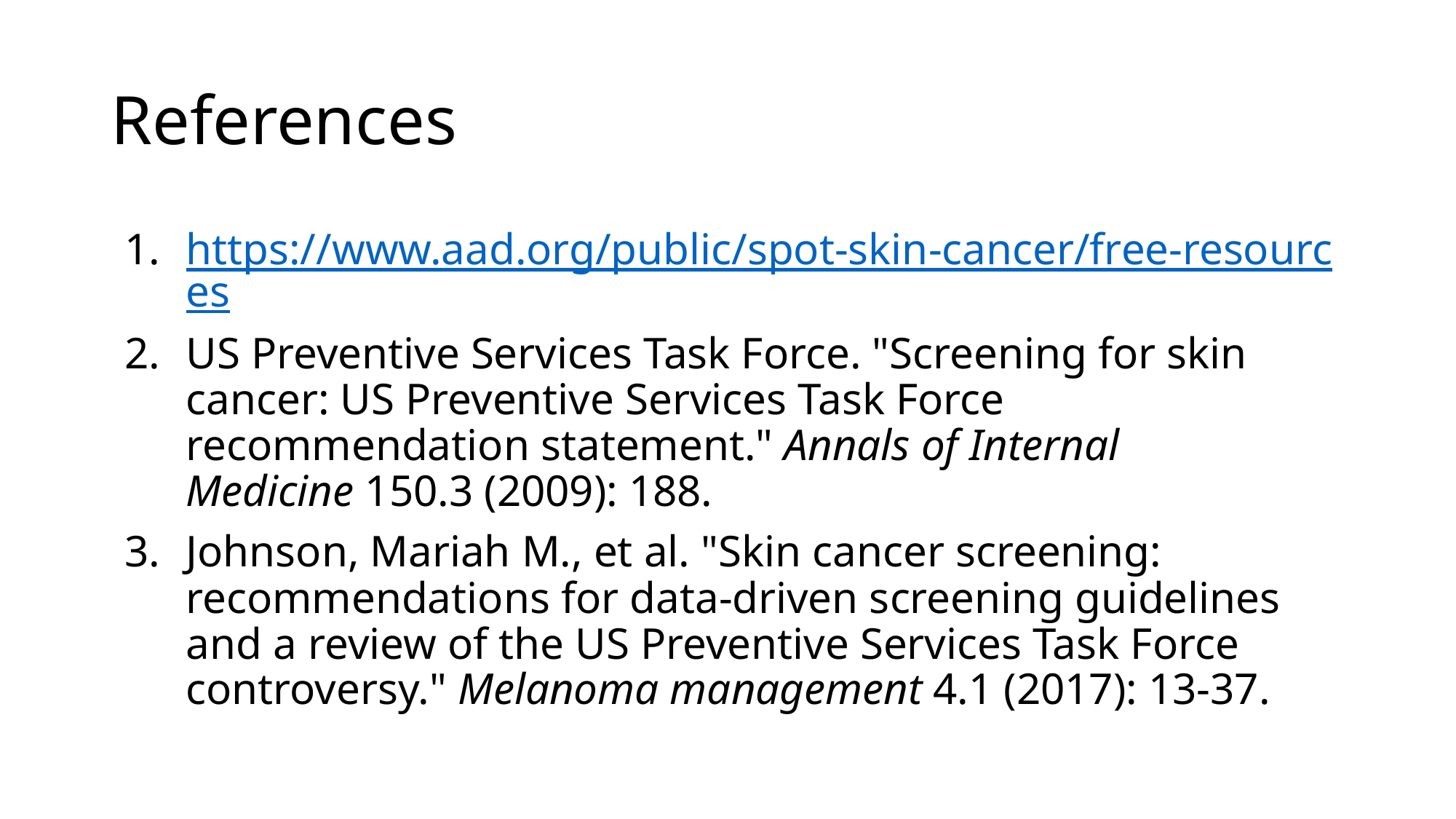

# References
https://www.aad.org/public/spot-skin-cancer/free-resources
US Preventive Services Task Force. "Screening for skin cancer: US Preventive Services Task Force recommendation statement." Annals of Internal Medicine 150.3 (2009): 188.
Johnson, Mariah M., et al. "Skin cancer screening: recommendations for data-driven screening guidelines and a review of the US Preventive Services Task Force controversy." Melanoma management 4.1 (2017): 13-37.
