# Supplementary material for: A Brief Dermatology Curriculum in Skin Cancer Detection and Prevention to Improve Medical Student Knowledge and Confidence
Source: MedEdPORTAL. 2020 Dec 29;16:11049. doi: 10.15766/mep_2374-8265.11049 (PMC7780741; doi:10.15766/mep_2374-8265.11049)
Supplement: Supplementary file 1 — Skin Cancer Prevention Didactic.pptxPretest Survey.docxImmediate Posttest Survey.docxSix-Month Possttest Survey.docxKnowledge Assessment Answer Key.docx [file mep_2374-8265.11049-s001.zip › C. Immediate Posttest Survey.docx]

The following is a short survey that will assess the impact of a didactic curriculum on medical students’ knowledge of sun-protective behaviors and skin cancer prevention.

**Post-test questionnaire: immediate follow up**

1. Which of the following is a pre-cancerous lesion?
2. Actinic keratosis
3. Dermatofibroma
4. Sebaceous hyperplasia
5. Seborrheic keratosis
6. Stucco keratosis
7. What is the most common type of cancer in the United States?
8. Basal cell carcinoma
9. Breast cancer
10. Colon cancer
11. Cutaneous squamous cell carcinoma
12. Melanoma
13. Which of the following skin lesions is associated with the highest risk of mortality if not detected at an early stage?
14. Actinic keratosis
15. Basal cell carcinoma
16. Melanoma
17. Sebaceous hyperplasia
18. Squamous cell carcinoma
19. Approximately ____ of Americans will develop a form of skin cancer in their lifetime?
20. 2%
21. 5%
22. 10%
23. 20%
24. 30%
25. The current guidelines by the U.S. Preventive Services Task Force (USPTF) recommend that the general adult population undergo visual skin cancer screening:
26. Every 6 months
27. Every 1 year
28. Every 2 years
29. At least once in a lifetime
30. There is no current recommendation by the USPTF
31. The International Agency for Research on Cancer, a division of the World Health Organization, classifies ultraviolet radiation and ultraviolet-emitting tanning devices as carcinogens in the same category as:
32. Alcohol
33. Lead
34. Metronidazole
35. Nitrogen mustard
36. Tobacco
37. Individuals should seek shade between the hours of ________ due to increased intensity of ultraviolet exposure at this time of day.
38. 8:00 am and 12:00 pm
39. 9:00 am and 3:00 pm
40. 10:00 am and 2:00 pm
41. 12:00 pm and 3:00 pm
42. 2:00 pm and 5:00 pm
43. The recommended frequency at which sunscreen should be reapplied is:
44. Every 1 hour
45. Every 2 hours
46. Every 3 hours
47. Every 4 hours
48. There is no need to reapply after the first application
49. SPF is an abbreviation for:
50. Solar protective factor
51. Sun protection factor
52. Sunscreen protection factor
53. Skin cancer prevention factor
54. Sunburn protection factor
55. The recommended minimum SPF for use when outdoors is:
56. 10
57. 20
58. 30
59. 40
60. 50
61. The term “broad spectrum” when used with regard to sunscreens indicates protection against while of the following ultraviolet (UV) rays?
62. UVA only
63. UVB only
64. UVA and UVB
65. UVB and UVC
66. UVA, UVB, and UVC
67. The recommended minimum amount of sunscreen for a single application to the entire body is:
68. 15 milliliters (0.5 fluid ounces or 1 tablespoon)
69. 30 milliliters (1 fluid ounce or 2 tablespoons)
70. 60 milliliters (2 fluid ounces or 4 tablespoons)
71. 90 milliliters (3 fluid ounces or 6 tablespoons)
72. 120 milliliters (4 fluid ounces or 8 tablespoons)
73. Which of the following is a physical (also known as mineral) sunscreen?
74. Avobenzone
75. Oxybenzone
76. Octylmethoxycinnamate
77. Para-aminobenzoic acid (PABA)
78. Titanium dioxide
79. If an individual is using a chemical sunscreen, it is recommended that she/she apply the sunscreen ______.
80. 2 hours before sun exposure
81. 1 hour before sun exposure
82. 30 minutes before sun exposure
83. Immediately at the time of initial sun exposure
84. Within 1 hour after initial sun exposure
85. The letter “E” in the ABCDEs mnemonic of melanoma detection stands for:
86. Ellipsoid
87. Equivalent
88. Erratic
89. Evenness
90. Evolution

Please rate the following on a scale of 1 to 5, where 1 means “not confident” and 5 means “extremely confident”

1. I am confident I can counsel my patients on the benefits of using sun-protective behaviors.

Not confident Somewhat confident Extremely confident

1 2 3 4 5

1. I am confident I can appropriately answer my patients’ questions on their risk of developing skin cancer.

Not confident Somewhat confident Extremely confident

1 2 3 4 5

1. I am confident that I can appropriately answer my patients’ questions on proper use of sunscreens.

Not confident Somewhat confident Extremely confident

1 2 3 4 5

1. I am confident I can appropriately refer patients for skin cancer screening.

Not confident Somewhat confident Extremely confident

1 2 3 4 5

Please rate the following on a scale of 1 to 5, where 1 means “not at all likely” and 5 means “extremely likely”

1. I am likely to ask my patients about their sun-protective practices during a routine social history.

Not at all likely Somewhat likely Extremely likely

1 2 3 4 5

1. I am likely to ask my patients about indoor tanning use during a routine social history.

Not at all likely Somewhat likely Extremely likely

1 2 3 4 5

1. I am likely to counsel my patients about recommended practices to prevent skin cancer.

Not at all likely Somewhat likely Extremely likely

1 2 3 4 5

1. I am likely to counsel my patients about the dangers of indoor tanning.

Not at all likely Somewhat likely Extremely likely

1 2 3 4 5

1. I am likely to recognize skin lesions that may be concerning for skin cancer during a routine physical examination.

Not at all likely Somewhat likely Extremely likely

1 2 3 4 5
