# Supplementary material for: A Brief Dermatology Curriculum in Skin Cancer Detection and Prevention to Improve Medical Student Knowledge and Confidence
Source: MedEdPORTAL. 2020 Dec 29;16:11049. doi: 10.15766/mep_2374-8265.11049 (PMC7780741; doi:10.15766/mep_2374-8265.11049)
Supplement: Supplementary file 1 — Skin Cancer Prevention Didactic.pptxPretest Survey.docxImmediate Posttest Survey.docxSix-Month Possttest Survey.docxKnowledge Assessment Answer Key.docx [file mep_2374-8265.11049-s001.zip › E. Knowledge Assessment Answer Key.docx]

**Knowledge Assessment Answer Key**

1. A (actinic keratosis)

2. A (basal cell carcinoma)

3. C (melanoma)

4. D (20%)

5. E (no current recommendation)

6. E (tobacco)

7. C (10 am - 2 pm)

8. B (every 2 hours)

9. B (sun protection factor)

10. C (30)

11. C (UVA & UVB)

12. B (30 mL)

13. E (titanium dioxide)

14. C (30 mins)

15. E (evolution)
